# Transcriptomic Analyses to Unravel *Cronobacter sakazakii* Resistance Pathways

**DOI:** 10.3390/foods13172786

**Published:** 2024-09-01

**Authors:** Shiyu Liu, Danliangmin Song, Biqi Liu, Kai Dong, Yujun Jiang, Chaoxin Man, Xinyan Yang, Feng Zhao

**Affiliations:** 1 Department of Food Science, Northeast Agricultural University, Harbin 150038, China; 18103646869@163.com (S.L.); 13161204248@163.com (D.S.); 13206658679@163.com (B.L.); dk283070004@126.com (K.D.); yujun_jiang@163.com (Y.J.); cxman@neau.edu.cn (C.M.); 15545116996@163.com (X.Y.); 2 Key Laboratory of Dairy Science, Ministry of Education, Harbin 150030, China

**Keywords:** acquired antibiotic resistance, *C. sakazakii*, mechanisms of resistance, transcriptomics

## Abstract

The proliferation of antibiotic usage has precipitated the emergence of drug-resistant variants of bacteria, thereby augmenting their capacity to withstand pharmaceutical interventions. Among these variants, *Cronobacter sakazakii* (*C. sakazakii*), prevalent in powdered infant formula (PIF), poses a grave threat to the well-being of infants. Presently, global contamination by *C. sakazakii* is being observed. Consequently, research endeavors have been initiated to explore the strain’s drug resistance capabilities, alterations in virulence levels, and resistance mechanisms. The primary objective of this study is to investigate the resistance mechanisms and virulence levels of *C. sakazakii* induced by five distinct antibiotics, while concurrently conducting transcriptomic analyses. Compared to the susceptible strains prior to induction, the drug-resistant strains exhibited differential gene expression, resulting in modifications in the activity of relevant enzymes and biofilm secretion. Transcriptomic studies have shown that the expression of glutathione S-transferase and other genes were significantly upregulated after induction, leading to a notable enhancement in biofilm formation ability, alongside the existence of antibiotic resistance mechanisms associated with efflux pumps, cationic antimicrobial peptides, and biofilm formation pathways. These alterations significantly influence the strain’s resistance profile.

## 1. Introduction

*Cronobacter sakazakii* (*C. sakazakii*) is classified as a foodborne pathogen and is considered to be the most pathogenic among the Cronobacter species [1]. These gram-negative organisms possess peripherally flagellated structures, enabling motility and the ability to infect individuals across all age groups. However, infections caused by *C. sakazakii* are particularly severe in low birth weight neonates and infants under six months of age. Clinical manifestations primarily include meningitis, necrotizing small bowel colitis [2], and respiratory infections, often leading to neurological complications and fatality rates ranging from 40–80% [3,4]. Furthermore, *C. sakazakii* can also impact immunocompromised adults, resulting in various symptoms such as bacteremia, appendicitis, sepsis, pneumonia, and wound infections [5,6]. Powdered infant formula (PIF) serves as the primary source of *C. sakazakii* infection in newborns [7]. Although breast milk is recognized as the optimal source of nutrition for newborns, parents may resort to feeding PIF due to inadequate breast milk supply or concerns about insufficient nourishment from breast milk. However, this choice increases the risk of newborns contracting diseases [8]. In recent years, reports of *C. sakazakii* contamination in powdered infant formula (PIF) production facilities have emerged globally. While efforts to mitigate *C. sakazakii* in the production environment have intensified, complete eradication of this contamination source remains unattained.

*C. sakazakii* exhibits remarkable resistance to environmental factors such as high temperature, heat, and ultraviolet light [9,10,11]. Consequently, it can be found on various food products and production equipment [12]. This includes powdered milk, cookies, wheat flour, corn flour, and various ready-to-eat foods [13,14]. *C. sakazakii* has also been identified on stainless steel and polyester plastics. To withstand environmental stresses, this pathogen can synthesize an anionic extracellular polysaccharide (ESP) that functions as a protective mechanism [15]. Bacterial adaptation to different environments can result in the formation of biofilms, which are dynamic ecosystems with spatial organization [15]. In the case of milk powder, biofilm formation is accelerated due to the presence of a surface layer in constant contact with nutrients [16].

In recent years, there has been growing concern regarding the antibiotic resistance of *C. sakazakii*. A survey by the Chinese Center for Disease Control and Prevention revealed a detection rate of 2.6% for *C. sakazakii* in 900 food samples collected from various factories in Jiangsu in 2017. The detection rates were higher in other regions, with 5.26% for samples from Guangxi and 6.3% for those from Changsha [17]. Further investigation revealed a close relationship between the tolerance of *C. sakazakii* and increased antibiotic resistance. A study on the detection of *C. sakazakii* in infant formula found resistance to amoxicillin, cefodoxime, streptomycin, methotrexate, and cefazolin in 77 infant formula samples and 75 dehydrated infant cereal samples [18]. Additionally, during antibiotic resistance determination of *C. sakazakii* collected from powdered infant formula, 83% of the strains were found to be resistant to more than one antibiotic [19]. Antibiotic resistance of *C. sakazakii* has also been investigated in China, where 78% of the strains tested showed resistance to vancomycin, lincomycin, cefazolin, amikacin, and neomycin. Among these, 54.6% were resistant to amikacin, and up to 68% were resistant to neomycin.

The historical use of antibiotics has previously been effective in reducing the morbidity and mortality caused by pathogenic bacteria. The introduction of kanamycin and streptomycin significantly reduced the incidence of tuberculosis. However, their widespread use has also contributed to the emergence of multidrug-resistant organisms, which substantially undermines antibiotic efficacy. Moreover, the overuse and misuse of antibiotics have facilitated the spread of antibiotic resistance genes across diverse environments, including soil, pastures, hospitals, and food sources [20]. The efficacy of single antibiotic treatments has diminished over time. Some studies have revealed that the resistance of *C. sakazakii* is associated with its gene regulation, and deleting specific genes in this bacterium generally reduces its antibiotic resistance. By evaluating the antibacterial activity of six antibiotics and examining the transcript levels of mutant and wild type strains, it was observed that the expression of genes related to drug resistance in the ABC transport system of *C. sakazakii* decreased by 1.5-fold [21]. Furthermore, investigations have indicated that *C. sakazakii* may enter a state of growth arrest when exposed to antibiotics, and during this stage, the expression of antibiotic resistance genes, particularly multidrug efflux pump genes, exhibits significant upregulation. Consequently, this leads to a strain with heightened antibiotic resistance [22]. Due to its exceptional resistance, *C. sakazakii* can be found in diverse environments. In the context of farm settings, antibiotic exposure may occur to prevent mastitis in dairy cows. Forsythe’s study demonstrated that elevated resistance has also been observed in clinically relevant environments [23].

Researchers in the field of antibiotic resistance have made significant progress in understanding the underlying mechanisms. Antibiotic resistance is influenced by multiple factors, including the efflux of antibiotics [20], mutations that disrupt antibiotic-target interactions or alter and shield antibiotics [24], and the presence of drug resistance traits within the genome [25]. Examples of such traits include increased expression of genes encoding efflux pumps [26], decreased outer membrane permeability [27], and enhanced biofilm production capacity [27]. Additionally, increased antibiotic tolerance is often accompanied by the production of virulence factors. For instance, Song et al. analyzed strains of *Cronobacter* obtained from infant formula and its processing environment and found that antibiotic tolerant strains expressed more virulence factors and proteins while maintaining their normal physiological functions [28]. The expression of efflux pump genes (AcrA, AcrB, TolC) increased, and the expression of the pore protein gene OmpC decreased in *Salmonella* Typhimurium when exposed to fluoroquinolones, leading to decreased expression of virulence related genes (stn and fimA) and increased expression of stress induced replacement factor σ rpoS. The drug-resistant strains exhibited better growth than nonresistant strains under high pressure conditions [29]. Similarly, Du et al. found that ciprofloxacin-resistant *Salmonella* exhibited decreased expression of invA and invE virulence genes, accompanied by reduced expression of the outer membrane protein OmpF [30]. Despite advancements in understanding antibiotic resistance, little is known about the mechanism of antibiotic resistance formation and the subsequent changes in virulence levels in *Cronobacter* strains. Transcriptomics is a valuable approach for investigating toxicological impacts, conducting risk assessment, exploring molecular pathways involved in environmental responses, and examining the impacts of environmental stressors or chemicals [30,31,32]. It aids in unraveling the molecular mechanisms of environmental responses and elucidating the modes of action of environmental stressors or chemicals [33]. By analyzing changes in gene expression levels, transcriptomics can help characterize alterations associated with antibiotic tolerance and identify changes in virulence levels, thus shedding light on the mechanisms of antibiotic resistance [34]. For example, Zhang et al. conducted comparative transcriptomic analysis and found that colistin and tobramycin caused dysregulation and expression changes in specific genes in *Pseudomonas aeruginosa*, respectively [27]. Similarly, transcriptomic studies have been conducted to investigate the response mechanism of *Pseudomonas aeruginosa* to cyanobacteria exposure, resulting in changes in intestinal flora and metabolism [35]. Furthermore, transcriptomics has been used to study the tolerance mechanism of microorganisms to lyophilized conditions under buffer salt stress, revealing the upregulation of specific genes related to fatty acid synthesis [36]. Although current literature mainly focuses on virulence factor changes, biofilm formation, and tolerance to environmental stress in *C. sakazakii*, there is limited research on drug resistance and its mechanisms. This study intends to employ a transcriptomics-based approach to analyze strains following the induction of antibiotic resistance and to explore the mechanisms underlying acquired drug resistance.

## 2. Materials and Methods

### 2.1. Materials

Tryptic Soy Broth (TSB) and Tryptic Soy Agar (TSA) media were purchased from Beijing Soleibo Bio-technology Co., Ltd. (Beijing, China). Absolute ethanol was purchased from China Zhiyuan Chemical Reagent Co., Ltd. (Tianjin, China). The Bacterial Genomic DNA Rapid Extraction Kit was purchased from BioTeKe Co., Ltd. (Beijing, China). All other solvents are analytical grade and purchased locally.

### 2.2. Stimulation of Antibiotic Tolerance in C. sakazakii

A series of tolerability experiments were conducted on 95 strains of wild *C. sakazakii* collected from production and processing environments in China (Table S1), utilizing a total of 24 antibiotics across 9 classes (Table S2). According to the American Society for Clinical and Laboratory Standards (CLSI) M100 antimicrobial susceptibility test implementation standard, the drug susceptibility results are interpreted standardly (Table S3). The XY001 strain, which exhibited sensitivity to five antibiotics—aminoglycosides, fluoroquinolones, penicillins, tetracyclines, and cephalosporins—was selected for the determination of the minimum inhibitory concentration (MIC) of these antibiotic classes using the microbroth dilution method recommended by the Clinical and Laboratory Standards Institute (CLSI). After the MIC of XY001 against the species antibiotic, the strain was induced to acquire resistance as follows: using TSB medium, activating XY001 at inoculation concentration of 2%, passage to a bacterial concentration of 10^8^ cfu/mL, inoculating the strain in TSB medium containing 1/4 MIC antibiotic concentration with 4% inoculation and incubating at 37 °C, 150 rpm. With 24 h as 1 passage, the antibiotic concentration in the medium is doubled after 3 passages until the strain is grown in medium at 4 MIC concentration, and the initial induction is considered successful. To assess the tolerance of the obtained strains, the induced strains were subcultured six times in tryptic soy broth (TSB) without antibiotics. The level of resistance to various antibiotics was then evaluated; a lack of significant difference in resistance levels indicated that the strains had acquired resistance.

### 2.3. Evaluation of the Test Tolerance of C. sakazakii after Induction

*C. sakazakii* XY001 after induction using different antibiotics was interpreted according to the CLS M100 drug susceptibility test implementation standard, and a total of 24 antibiotics in 9 classes were cross-resistance experiments.

### 2.4. Transcriptome Sequencing Analysis before and after Strain Induction

In order to explain the tolerance changes of *C. sakazakii* before and after the induction of different antibiotics from the molecular mechanism, transcriptome methods were used to study them. The induced strain was inoculated in TSB medium at 2% inoculation amount, cultured at 37 °C for 12 h, centrifuged at 4 °C, 8000 rpm for 20 min, the supernatant was removed, the lower cells were rinsed with sterile water 3 times, and quickly frozen in liquid nitrogen and stored at −80 °C. Sequencing and assembly of *C. sakazakii* RNA sequences using the Illumina MiSeq II sequencing platform (Shanghai OE 180 Biotech Co., Ltd. (Shanghai, China). The Gene Ontology (GO) and Kyoto Encyclopedia of Genes and Genomes (KEGG) databases were used to analyze *C. sakazakii* differentially expressed genes (DEGs). The double ended sequencing data of *C. sakazakii* XY001 induced using 5 antibiotics was obtained through the Illumina platform, and the data were preprocessed using FASTQ software (FastQC v0.11.9) [37]. This method is used to compile statistics on the number of reads throughout the entire quality control process. It is employed for quality inspection to determine the success of the transcriptome sequencing.

### 2.5. Validation of Gene Expression by Real-Time Quantitative Reverse Transcription–Polymerase Chain Reaction

To verify gene expression changes, total RNA was reverse-transcribed using Prime scriptTM RT Reagent Kit (Takara, Japan) and real-time quantitative PCR (qRT-PCR) using SYBRR qPCR Master Mix (Med-ChemExpress, Princeton, NJ, USA). The reaction was run in the Real-Time PCR System (Applied Biosystems 7500, Foster City, CA, USA). The relative expression rate (2^−ΔΔCt^) was calculated using 16S rDNA as internal reference and comparative period threshold method. All qRT-PCR assays were performed in 3 parallel experiments.

### 2.6. Data Analysis

All experiments were set up in three parallel sets, data using GraphPad Prism 8 (Prism 8.1.0) and Origin pro software (Origin pro9.5.1) to analyze the meaning of the data and plot the results. Transcriptome-related bioinformatics analysis was performed in https://cloud.oebiotech.com/task/ (accessed on 5 September 2023) using the OECloud tool. Volcano charts (or other graphs) were plotted based on R (https://www.r-project.org/, accessed on 5 September 2023) on the OECloud platform (https://cloud.oebiotech.com/task/, accessed on 5 September 2023).

## 3. Results and Discussion

### 3.1. Evaluation of Cross-Resistance in C. sakazakii after Antibiotic Induction

Table 1 presents the minimum inhibitory concentration (MIC) values of strain XY001 before and after antibiotic induction. The data clearly show that as the antibiotic concentration increases, the MIC of the strain also increases gradually. This indicates that antibiotics have the ability to induce the development of resistance in the strain, making it more robust. It is noteworthy that *C. sakazakii* exhibits stable growth across a wide range of antibiotic concentrations, with considerable variation in the MIC values. According to the antibiotic resistance MIC values provided by CLSI (Clinical and Laboratory Standards Institute), the induced XY001 strain has been determined to have reached resistance levels. To ensure the absence of contamination during the antibiotic induction process, DNA was extracted from the strains before and after induction. Gel electrophoresis and 16S rDNA sequencing were performed, confirming the absence of contamination. Moreover, resistance tests conducted on different antibiotics from the same class revealed that after the induced strain acquired resistance, resistance to other antibiotics within the same class also developed.

Table 2 shows that the resistance of antibiotic-sensitive strain XY001 to cephalosporins and penicillin antibiotics increases after induction. The aforementioned β-lactam antibiotics have the ability to modulate the permeability of cell membrane proteins in the strain, resulting in autolysis. In addition to these β-lactam antibiotics, the resistance to tetracyclines, aminoglycosides, peptides, and sulfonamides antibiotics has also significantly increased. Aminoglycoside antibiotics primarily function by forming kinetic complexes with the 70S ribosome and selectively binding to target proteins on the 30S subunit. This interaction obstructs the binding of stop codons to the ribosomal nucleoprotein and increases membrane permeability. Upon acquisition of resistance to aminoglycoside antibiotics, the susceptibility to penicillin, tetracycline, fluoroquinolone, and sulfonamide antibiotics in XY001 significantly decreased, while the resistance to other antibiotics remained relatively stable. Similarly, tetracycline resistant strains exhibited increased resistance to penicillins, cephalosporins, and sulfonamide antibiotics. Fluoroquinolone antibiotics impede DNA synthesis and replication by targeting the A subunit of bacterial DNA helicase, resulting in bacterial cell death. Strains that develop resistance to fluoroquinolones exhibit significant resistance to penicillin and macrolide antibiotics, with a moderate increase in resistance to other antibiotic classes. Generally, penicillin-induced strains and cephalosporin-induced strains exhibited increased resistance to other antibiotics after acquiring antibiotic resistance. The table demonstrates that penicillin-induced *C. sakazakii* strains exhibited higher resistance compared to cephalosporin antibiotics.

### 3.2. Illumina Sequencing and Quality Assessment

The double ended sequencing data of *C. sakazakii* XY001 induced using five antibiotics was obtained through the Illumina platform, and the data were preprocessed using FASTQ software [37]. Statistics summarize the number of reads throughout the quality control process, as shown in Table 3.

The original reads were filtered and quality checked (https://www.ncbi.nlm.nih.gov/assembly/GCF_003516125.1/, accessed on 24 September 2023). The M reads for XY001, XY001-amp, XY001-ami, XY001-tet, XY001-ofl, and XY001-ceo groups were observed to be 16.63, 16.15, 16.65, 15.96, 15.41, and 16.78, respectively. The proportion of effective bases in all groups exceeded 78.14%, while the proportion of bases with a Phred value greater than 30 exceeded 91.63%. Furthermore, the GC content proportion exceeded 54.82%. These findings indicate the successful transcriptome sequencing of the five major antibiotic-induced strains, thus providing a solid foundation for subsequent analysis of the sequencing results.

### 3.3. Comparative Analysis with Reference Genomes

Rockhopper software (Rockhopper version 2.0.3) was utilized to compare the clean reads against the designated reference genome, thereby enabling the retrieval of gene and reference gene positional information and facilitating the analysis of the sequence characteristics of the sequenced sample. As shown in Table 4. For the original strain and the five antibiotic induced strains (XY001, XY001-Amp, XY001-Ami, XY001-Tet, XY001-Ofl, and XY001-Ceo), the localization ratios to the reference genome were found to be 71.00%, 73.67%, 76.33%, 75.33%, 77.67%, and 63.33%, respectively. These results indicate that the localization ratios for all strains exceeded 70%, suggesting the suitability of the selected reference genome for sequence alignment and confirming that the strains were not contaminated. Furthermore, the corresponding read numbers were 2,346,514, 2,138,079, 1,955,717, 1,950,504, 1,830,473, and 2,964,588, accounting for 29.00%, 26.33%, 23.67%, 24.67%, 22.33%, and 36.67%, respectively. By comparison, it can be observed that, compared to XY001, the gene expression of strains induced by different types of antibiotics has shown certain changes, which are not completely identical to XY001. It can be inferred that antibiotic induction can affect the level of gene expression within the strains, providing support for subsequent analysis.

### 3.4. Analysis of C. sakazakii DEGs before and after Antibiotic Induction

**Figure 1:**
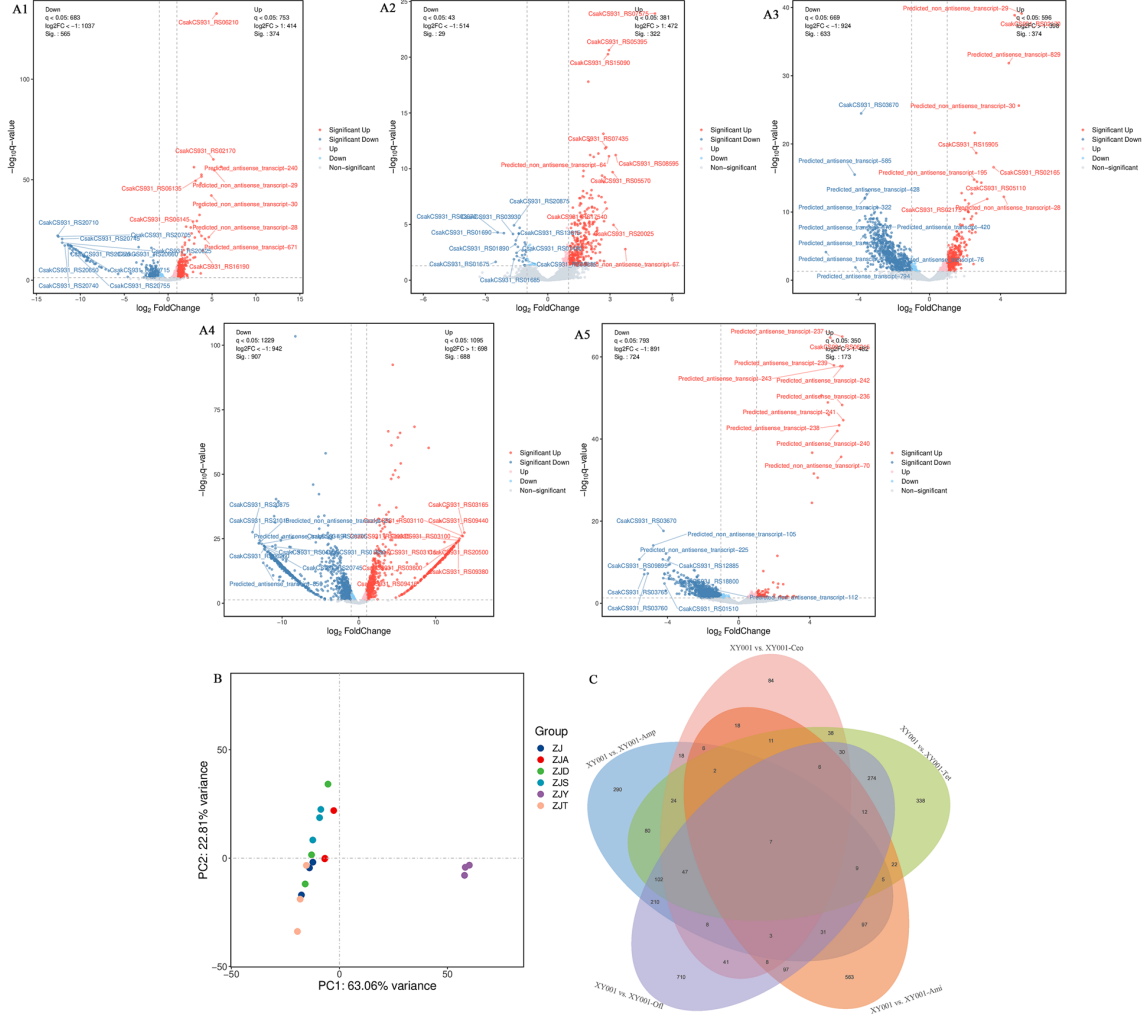
Gene expression of *C. sakazakii* induced by different classes of antibiotics. (**A1**) XY001 vs. XY001-Amp; (**A2**) XY001 vs. XY001-Ami; (**A3**) XY001 vs. XY001-Tet; (**A4**) XY001 vs. XY001-Ofl; (**A5**) XY001 vs. XY001-Ceo; (**B**) Results of principal component analysis between different groups.; (**C**) Differentially expressed genes in five antibiotic resistant *C. sakazakii* strain.

Gene expression profiles of *C. sakazakii* were analyzed using the Cuffdiff program to examine the effects of different antibiotics on its induction. The control group comprised of XY001 prior to induction and served as a reference for gene expression comparisons in the antibiotic-induced strains. The results illustrated in Figure 1 demonstrate significant changes in gene expression following induction with various antibiotics. Specifically, ampicillin induction led to significant changes in the expression of 957 genes, with 386 genes upregulated and 571 genes downregulated Figure 1(A1,B). Amikacin induction resulted in significant changes in 349 genes, with 322 genes upregulated and 27 genes downregulated Figure 1(A2,B). Similarly, the Tet group exhibited significant changes in 1027 genes, with 387 genes upregulated and 640 genes downregulated Figure 1(A3,B). Additionally, the Ofl group showed significant alterations in 1470 genes, with 784 genes upregulated and 956 genes downregulated Figure 1(A4,B). Following ceftriaxone induction, 927 genes exhibited significant changes, with 182 genes upregulated and 745 genes downregulated Figure 1(A5,B). These results indicate that the gene expression level of *C. sakazakii* is comparatively less affected by amikacin induction than by other antibiotics. Differences in gene expression also contribute to the variation in sample similarity, consistent with the findings in Figure 1C. It is evident that the use of different classes of antibiotics further modulates gene regulation in the induction strain.

### 3.5. GO Annotation of DEGs before and after Induction by C. sakazakii

The GO annotation method was used to determine the differentially expressed genes (DEGs) in *C. sakazakii* before and after induction, and to elucidate the related biological processes. The analysis revealed 64 subclasses, with a total of 3129 genes participating in these processes. Following the identification of DEGs, GO enrichment analysis was performed on these genes to visually demonstrate the expression of genes responsible for different subclasses in different processes. The GO analysis indicated that the dominant gene categories and subclasses induced by different antibiotics in *C. sakazakii* were generally consistent. Based on the gene changes in different biological processes, the main functions regulated by the major categories were in three parts: “biological process”, “cellular component”, and “molecular function”. The main biological processes in the subclasses included the regulation of cellular and metabolic processes. In terms of cellular components, the gene subclasses were mainly associated with cell parts and membrane parts. Regarding molecular function, the gene subclasses predominantly included binding-related genes and catalytic activity related genes.

**Figure 2:**
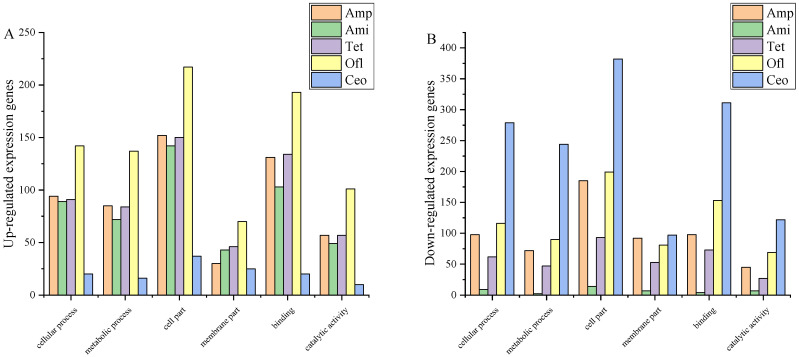
Histogram of gene expression changes of *C*. *sakazakii* DEGs after induction by five classes of antibiotics. (**A**) Upregulation of expression genes; (**B**) down-regulation of expressed genes.

As shown in Figure 2A,B, an analysis was conducted on the more prominently expressed subclasses of genes within different major categories. In the Amp, Ami, Tet, Ofl, and Ceo treatments, the genes responsible for regulating “cellular processes” were respectively upregulated (94, 89, 91, 142, 20) and downregulated (98, 9, 62, 116, 279); those responsible for regulating “metabolic processes” were respectively upregulated (85, 72, 84, 137, 16) and downregulated (72, 2, 47, 90, 244); those responsible for regulating “cellular components” were respectively upregulated (152, 142, 150, 217, 37) and downregulated (185, 14, 93, 199, 382); those responsible for regulating “membrane components” were respectively upregulated (30, 43, 46, 70, 25) and downregulated (92, 7, 53, 81, 97); those responsible for regulating “binding activities” were respectively upregulated (131, 103, 134, 193, 20) and downregulated (98, 4, 73, 153, 311); and those responsible for regulating “catalytic activities” were respectively upregulated (57, 49, 57, 101, 10) and downregulated (45, 7, 27, 69, 122).

The examination of gene distribution across subclasses revealed significant alterations in the cellular and membrane compositions of *C. sakazakii* following antibiotic induction. These modifications imply that the strain may have acquired antibiotic-resistant compounds during the induction process. In addition to producing antibiotic-resistant substances, the acquisition of Burkholderia cenocepacia resistance may be attributed to the production of metabolites capable of degrading antibiotics or the enhancement of biofilm formation. It is worth noting that the presence of virulence factor Omp on the biofilm contributes to changes in the virulence of Burkholderia cenocepacia [38,39]. Furthermore, changes in cell membrane permeability hinder the effective action of antibiotics within bacteria, thereby shortening the duration of antibiotics’ effectiveness against bacteria [40,41]. Moreover, the analysis also revealed significant changes in genes responsible for regulating “transport activities” in the strains induced by the five antibiotics. It can be speculated that this is due to the action of efflux pumps located on the bacterial cytoplasmic membrane. Efflux pumps primarily function by reducing the intracellular concentration of antibiotics, thus prolonging survival. Consequently, metabolites are rapidly secreted from the cell to the external environment, enhancing the transport capacity of substances within the cell and minimizing cell damage.

### 3.6. Functional Enrichment Analysis of DEGs

**Figure 3:**
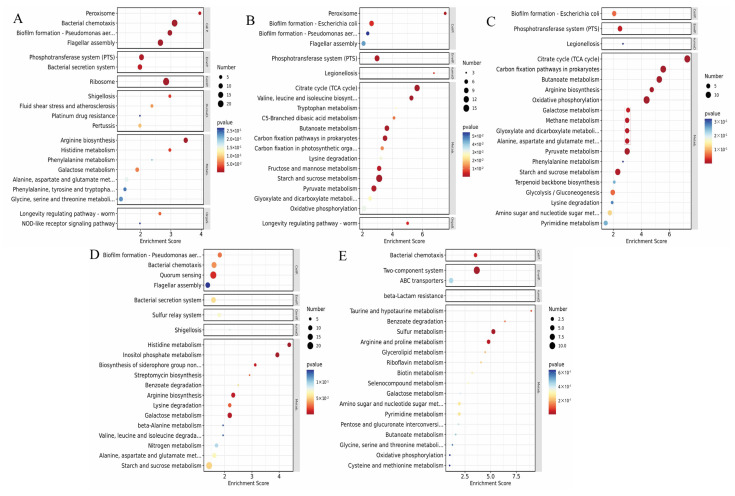
Scatter diagram of KEGG annotation enrichment pathways of DEGs in *C. sakazakii* strains induced with five antibiotics: (**A**) XY001-Amp; (**B**) XY001-Ami; (**C**) XY001-Tet; (**D**) XY001-Ofl; (**E**) XY001-Ceo.

The differential expression genes (DEGs) of the strains were located in the KEGG database to identify enriched pathways using transcriptome analysis. Diagrams illustrating the upregulated and downregulated genes in each antibiotic-induced strain for KEGG enrichment analysis are included in the Supplementary Materials. As shown in Figure 3, the Amp group had 349 genes mapped to 49 pathways, the Ami group had 19,212 genes mapped to 35 pathways, the Tet group had 279 genes mapped to 38 pathways, the Ofl group had 454 genes mapped to 51 pathways, and the Ceo group had 491 genes mapped to 42 pathways. Among them, the amino acid metabolism pathway (28.28%, 22.32%, 19.44%, 21.82%, 10.49%) and the membrane transport pathway (27.08%, 27.86%, 22.99%, 21.74%, 17.24%) had the highest proportions.

The enriched pathways of the five classes of antibiotic-induced strains are shown in Figure 3. The bubble chart of the top 20 KEGG enrichment analysis is shown. The horizontal axis indicates the enrichment score, while the bubble size is positively correlated with the number of differentially expressed protein-coding genes associated with each entry. Figure 3 shows that compared to the control group, *C. sakazakii* induced by ampicillin is mainly enriched in pathways such as “bacterial chemotaxis”, “biofilm formation”, and “flagellar assembly regulation” (Figure 3A). The expression of genes related to these three pathways all showed a certain decrease. This indicates that the addition of antibiotics induces strains to regulate bacterial cell activity through signal molecules, reducing the ability to form biofilms. Chemotaxis mainly controls cell movement. During the antibiotic induction process, cells sense changes in chemical concentrations around them and then move to more favorable conditions. In this process, receptors control the autophosphorylation of CheA group amino acid kinases, which leads to changes in bacterial behavior, such as flagellar rotation direction or speed. Significantly, the formation of biofilms is influenced by various signals and critical pathways, including the activity of the flagellar system. This explains why genes regulating bacterial chemotaxis and biofilm formation are downregulated together with genes regulating flagellar assembly. However, genes related to metabolism and amino acid metabolism showed increased expression, indicating that in the presence of antibiotics, bacteria increase their metabolism to reduce the accumulation of toxic substances inside the cell, thus developing resistance to ampicillin.

For the amikacin-induced resistant strains, DEGs are mainly concentrated in pathways such as “biofilm formation”, “phosphotransferase system”, and “citric acid cycle” (Figure 3B). The expression of genes in these three pathways showed a significant increase. This suggests that amikacin mainly induces bacterial cell activity, affecting the strain’s utilization of nutrients. The phosphotransferase system (PTS) is the main mechanism for bacteria to take up carbohydrates, mainly including sugar alcohols and disaccharides. In an antibiotic environment, bacteria accelerate the absorption of nutrients necessary for their own growth. Along with this process, the TCA cycle speeds up, accelerating the uptake of carbon source substances. While meeting their own growth needs, this also enhances the metabolic process of substances, thus increasing their own vitality and reducing cell density. Although the expression of biofilm formation also increased, the expression of flagellar assembly genes and bacterial chemotaxis genes associated with this activity decreased, indicating that amikacin reduces the motility of bacteria, while the increased metabolism of bacteria to some extent reduces its impact, leading to resistance.

In tetracycline-resistant strains, differential expression genes (DEGs) are mainly enriched in pathways such as the “citric acid cycle”, “oxidative phosphorylation”, and “butyrate metabolism”, with enhanced gene expression (Figure 3C). Conversely, gene expression is downregulated in pathways related to “bacterial chemotaxis” and “flagellar assembly regulation”, indicating that tetracycline inhibits the mobility of strains during growth. Oxidative phosphorylation, as an important pathway for cell response to produce ATP, can work in conjunction with the citric acid cycle to provide nutrients for the organism, playing a significant role in bacterial antibiotic resistance. Additionally, the bacterial metabolite butyrate can enhance bacterial resistance. Research has shown that butyrate can inhibit the enrichment of harmful tissues by downregulating the expression of adhesion-related outer membrane proteins (RadD, Foma, FadA), alleviating antibiotic resistance caused by chemical agents [42]. This indicates that tetracycline induces strains to secrete signaling molecules, influencing the expression of genes related to cellular activity, enhancing their metabolic capacity, and secreting substances that inhibit damage from antibiotics to improve bacterial resistance.

For ofloxacin-induced resistant strains, it can be observed that during antibiotic induction, ofloxacin reduces the gene expression related to cell regulation of the external environment, such as “biofilm formation”, “bacterial chemotaxis”, “flagellar assembly”, and “quorum sensing”. Only multiple amino acid metabolism and “galactose metabolism” related gene expressions are upregulated (Figure 3D). This may represent a novel mechanism to enhance resistance. Studies have shown that certain amino acids can increase bacterial resistance to antibiotics. For example, aspartic acid can enhance Pseudomonas aeruginosa resistance to neomycin sulfate [43]. Additionally, glycine, serine, and alanine can activate the TCA pathway, increasing bacterial metabolism, while glutamic acid can enhance the sensitivity of resistant bacteria to aminoglycoside drugs through the P cycle (pyruvate-acetyl-CoA-TCA cycle) by increasing NADH and PMF [44]. This indicates that ofloxacin-induced strains interact with cells, altering intracellular substance transport capabilities, promoting nucleic acid interactions between cells, and ultimately enhancing resistance by increasing amino acid metabolism. Notably, the MIC results presented in Table 1 demonstrate that the MIC for ofloxacin-induced strains is consistently lower than that of the other four groups. This suggests that the role of amino acid metabolism in countering antibiotic action remains limited, aligning with the aforementioned findings.

After acquiring resistance to ceftriaxone, DEGs are mainly upregulated in pathways such as the “two-component system”, “sulfur metabolism”, and amino acid metabolism pathways (Figure 3E). In addition to amino acid metabolism mentioned earlier, the two-component system allows bacteria to perceive, respond to, and adapt to changes in the environment or intracellular state. By jointly regulating gene expression through sensor proteins-histidine kinases (HK) and response regulator proteins, it impacts physiological changes in cells to respond to external stimuli. This indicates that ceftriaxone-resistant strains secrete antibiotic-resistant substances, accelerate the delivery of metabolites in the body, ultimately manifesting as antibiotic resistance.

The aforementioned result analysis reveals that the functional enrichment analysis of differentially expressed genes (DEGs) provides insights into the alterations of specific regulatory genes in *C. sakazakii* during the process of resistance to different antibiotics. The specific mechanisms underlying the acquisition of antibiotic resistance can be elucidated by examining the up-regulation or down-regulation of gene numbers within the relevant pathways.

### 3.7. Drug Resistance Genes and Resistance Mechanisms of C. sakazakii after Induction of Class 5 Antibiotics

The resistance mechanism of *C. sakazakii* to different classes of antibiotics was elucidated through RNA-Seq analysis following induction of class 5 antibiotics. The Comprehensive Database on Antibiotic Resistance (CARD) was employed as a reference for genetic information annotation.

In the XY001-Amp group, a total of 36 antibiotic resistance-related genes were identified, with 22 showing up-regulation and 14 down-regulation (Table S4). Using a differential factor log_2_Foldchange >1 criterion, it was observed that the gene expression of ampC increased by 5.51-fold after induction with penicillin antibiotics. This gene is responsible for regulating β-lactamase secretion and antibiotic drug metabolism. Additionally, the gene expressions of b1256 and Z2647 increased by 1.55-fold and 1.21-fold, respectively. These genes are responsible for regulating the transcriptional regulator of the LysR family and glutathione transferase GstA, which are involved in drug and glutathione metabolism, as well as drug resistance. These findings suggest that under the induction of penicillin antibiotics, the intracellular protein stability of the strain is enhanced, leading to improved drug metabolism. Moreover, upon acquisition of drug resistance, the expression of LysR family transcriptional regulators affects DNA binding, regulation of secondary metabolic biosynthesis processes, and NAD + ADP ribosyl transferase activity.

Under the influence of ampicillin, changes in osmotic pressure trigger the osmotic pressure sensor histamine kinase, resulting in up-regulation of the regulatory gene of outer membrane porin C. Consequently, the drug can enter from the extracellular membrane. In the oligopeptide transport system, a gene indirectly influences the β-lactamase-induced signal sensor and substrate binding protein, leading to the production of β-N-acetyl hexosamine berry through reaction with peptides containing DAP. Subsequently, LysR family transcriptional regulators inhibit DNA synthesis. Additionally, under the influence of fimbrial Z protein, outer membrane porin C synthesizes CMY-1, which is subsequently expelled into the environment via efflux pumps, thereby promoting the degradation of ampicillin.

**Figure 4:**
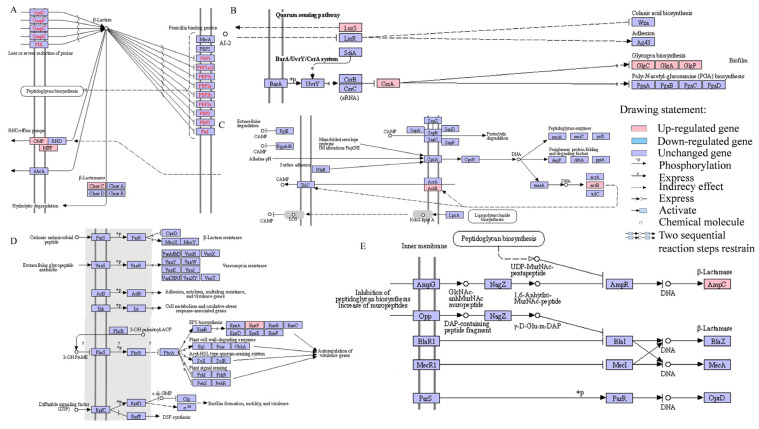
Major pathways of *C. sakazakii* resistance to different classes of antibiotics. (**A**) XY001-Amp; (**B**) XY001-Ami; (**C**) XY001-Tet; (**D**) XY001-Ofl; and (**E**) XY001-Ceo.

A total of 66 genes associated with biofilm formation were screened, with 33 genes being upregulated and 33 genes downregulated. Specifically, ESA-00581, Z4760, and Z4389 were upregulated by 1.13-fold, 1.28-fold, and 1.04-fold, respectively. These findings indicate an increased production of S-ribosylhomocysteine lyase and 3’,5’-cycloAMP phosphodiesterase, suggesting that the effect of antibiotics on bacteria enhances the metabolism of cysteine and methionine, and regulates the phosphorylation reaction regulator (RR) of histidine kinase (HK) to facilitate signal transduction and promote biofilm formation. Furthermore, this process accelerates purine metabolism and elevates the intracellular substance metabolism rate, thereby enhancing bacterial resistance to penicillin antibiotics (Figure 4A).

In the XY001-Ami group, a total of 81 genes related to antibiotic resistance were analyzed, with 54 showing up-regulation and 27 showing down-regulation (Table S5). Based on a log_2_Foldchange >1 criterion, the expression of ACIAD3023, a regulatory gene for resistance proteins, increased by 2.39-fold. Furthermore, the expression levels of glutathione transferase regulatory genes increased by 1.89-fold, the drug resistance protein MarB by 1.79-fold, and the biofilm peroxide resistance protein BsmA by 1.72-fold. The corresponding regulator of the two-component system, PhoP, also exhibited significant up-regulation (1.06-fold). Intracellular proteases, such as TolC, gstA, and STM3997 genes, were upregulated to a certain extent, resulting in the stable synthesis of related genes. On the other hand, the expression of B1113, B1846, and BSU00260 genes was downregulated (1.09, 1.07, and 1.09 times, respectively). Overall, the induction of antibiotic resistance in the strain improved its transport and efflux capabilities.

Upon entry of the drug into the strain’s outer membrane, the expression of amino kinase CpxA increased, leading to the formation of response regulator CpxR. This, in turn, increased the expression of peptidogly canase related aminoenzymes and transpeptidase YcfS. The response regulator CpxR also upregulated the expression of mar-sox-rob regulatory activators, promoting the up-regulation of multi drug efflux pumps and outer membrane proteins within the multi-drug efflux system. Serine protease and protein dithiol enhanced the levels of oxidoreductase and the expression of trans-isomerase (cyclophilin A), which contributed to the mis-folding of outer membrane proteins upon drug entry. Additionally, degradation factors were associated with this process.

A total of 67 genes related to biofilm formation were screened, with 43 showing up-regulation and 24 showing down-regulation. Specifically, c1327, ESA-00581, and PA0085 increased by 2.10-fold, 1.84-fold, and 2.31-fold, respectively. These findings indicated an increased secretion of biofilm formation regulators, such as BssS, S-ribosylhomocysteine lyase, and type VI secretion system tubular protein Hcp. Bacterial quorum sensing was also enhanced, leading to increased resistance against antibiotic substances and accelerated biofilm formation. This ultimately increased resistance to aminoglycoside antibiotics (Figure 4B).

In the XY001-Tet group, a total of 70 genes associated with antibiotic resistance were screened, with 48 showing increased expression and 22 showing decreased expression (Table S6). Notably, the ramA gene exhibited the most significant increase in expression, with a fold change of 4.79. This gene encodes the RamA family antibiotic efflux transcriptional regulator. The b1526 gene, responsible for the LysR family transcriptional regulator, showed a 1.33-fold increase in expression. Additionally, the expression levels of genes b4189 and SF1149, which regulate the biofilm antiperoxide protein BsmA and the two-component system response regulator PhoP, respectively, increased by 1.81 and 1.49 times. The upregulated genes and related pathways associated with tetracycline-induced drug resistance appear to be related to the resistance mechanisms of aminoglycosides, CAMP resistance, and two-component systems. The tetracycline resistance acquired by the strain is shown in Figure 4C.

As the drug concentration increases, the expression of EIIA component related genes on the outer membrane of the glucose PTS system is regulated, leading to increased production of adenylyl cyclase. These enzymes enhance the expression of AMP receptor proteins, which accumulate during the attachment phase of biofilm formation. The AMP receptor protein also contributes to biofilm formation through a population effect. The two-component system regulates the repressor protein RsmA, and the release of RsmA can enhance the toxicity of the strain.

A total of 67 genes related to biofilm formation were screened, with 42 showing up-regulation and 25 showing down-regulation. Notably, PA0085 was upregulated by 2.59 times, c1327 was upregulated by 1.62 times, and STY3620 was upregulated by 1.24 times. These findings suggest an increased expression of class I adenylyl cyclase, type VI secretion system tubular protein Hcp, and biofilm formation regulator BssS. This results in augmented purine metabolism and enhanced capacity for biofilm formation in bacteria. Consequently, the impact of tetracycline on the strain is blocked, resulting in resistance.

A total of 83 genes associated with drug resistance were investigated in the XY001-Ofl group. Among them, 52 genes showed up-regulation, while 31 genes showed down-regulation (Table S7). Notably, the expression of the DUF2724 domain containing protein was increased by a fold change of 10.33. Additionally, the expression of the T1E_0241 gene, encoding the outer membrane subunit of the efflux transporter, increased by 1.54 times. Meanwhile, the expression of the protein encoded by the B1243 gene increased by 7.65 times, and the expression of the B1243 gene itself increased by 1.46 times. Among the downregulated genes, the expression of the Arnit_2199 gene, associated with the biofilm formation pathway, decreased by 10.73 times. The ECP_0940 gene, responsible for the regulation of outer membrane protein F, showed a decrease in expression by 8.27 times. Furthermore, the expression of the ML0773 gene decreased by 11.12 times. These findings suggest that the acquisition of fluoroquinolone resistance may also increase the strain’s resistance to β-lactamase antibiotics.

The mechanism of resistance to ofloxacin in the strain is illustrated in Figure 4D. *C. sakazakii* reduces the environmental concentration of ofloxacin through amino acid starvation, indirectly leading to an increase in the expression of sigma factors via guanosine 3’-diphosphate 5’-triphosphate. Consequently, this elevates the expression of outer membrane proteins, including OprD and OmpC. Concurrently, the secretion of bifunctional diguanylate cyclase/phosphodiesterase, undecenyl photransferase, and MerR family transcriptional regulators is significantly increased, collectively promoting biofilm formation and improving the elimination of toxic substances to enhance resistance to ofloxacin.

A total of 40 genes associated with drug resistance were examined in the XY001-ceo group. Among these, 17 genes were found to be upregulated, whereas 23 genes exhibited down-regulation (Table S8). Notably, the ampC gene, associated with class C β-lactamase CSA-1, demonstrated an up-regulation with a fold change of 4.66. On the other hand, the expressions of ESA_00711, b2435, SL1344_2237, and STM3472 were downregulated by 2.48, 2.42, 2.50, and 2.12 times, respectively. It is worth mentioning that most of the upregulated genes had a log_2_Foldchange level smaller than one, suggesting that the acquisition of drug resistance in this group primarily occurs through enhanced expression of class C β-lactamase, which enables significant binding and degradation of antibiotics.

The development of resistance in this group of strains is primarily associated with the two-component system and the CAMP resistance pathway, as illustrated in Figure 4E. Similar to the Ami group, the action of ceftriaxone induces misfolding of intracellular membrane proteins in *C. sakazakii*. Simultaneously, a large quantity of C-β lactamases is produced and transported outside the cell through the efflux pump to bind with antibiotics.

### 3.8. Virulence Genes of C. sakazakii after Induction by Five Classes of Antibiotics

The RNA-Seq analysis of strains exposed to five different classes of antibiotics was conducted to assess and analyze changes in virulence factors. The genetic information was annotated using the virulence factor database (VFDB) as a reference for analysis.

Among the strains resistant to ampicillin, a total of five virulence related genes were identified (Table S4). Two of these genes were upregulated, while three were downregulated. Further analysis was carried out using a threshold of log_2_Foldchange >1. In the case of ampicillin, amikacin, and tetracycline-induced resistant strains (Tables S5 and S6), the virulence factors SfrB and VirK were upregulated by 0.44 and 0.94 times, respectively, while the nontoxic site protein ImpE and virulence factor BrkB family protein were downregulated by 0.79, 0.52, 0.89, and 0.75 times, respectively. For strains resistant to ofloxacin (Table S7), five virulence related genes were identified, with three being upregulated and two being downregulated. Specifically, the b0877 gene showed a 0.10-fold upregulation, while ESA_04062 exhibited a 0.76-fold increase, indicating an increase in the expression of virulence factor VirK and BrkV family proteins. In contrast to the earlier strains, the PA0086 gene exhibited a 1.25-fold downregulation, indicating a reduction in the secretion of the non-toxic protein ImpE. In the case of ceftriaxone resistant strains, four virulence-related factors were upregulated, while one related gene was downregulated (Table S8). The expression of b0877 was downregulated by 1.52 times, and the virulence factors BrkB, SfrB, VirK, and non-toxic proteins were also downregulated.

Based on the above analysis, it can be deduced that in terms of virulence, the impact of the five classes of antibiotics on strains primarily affects the expression of b0877, PA0086, and ESA_08062 genes. These changes in gene expression influence the secretion of proteins and virulence factors, thereby altering the resistance of bacteria.

### 3.9. Validate DEGs Using qRT-PCR

To validate the findings obtained from the transcriptome analysis, quantitative real-time PCR (qRT-PCR) was performed on XY001-Amp, Ami, Tet, Ofl, and Ceo strains induced by the five antibiotics. A total of 11 genes associated with antibiotic resistance and virulence were selected for verification, and the gene names and sequences can be found in Table S9.

**Figure 5:**
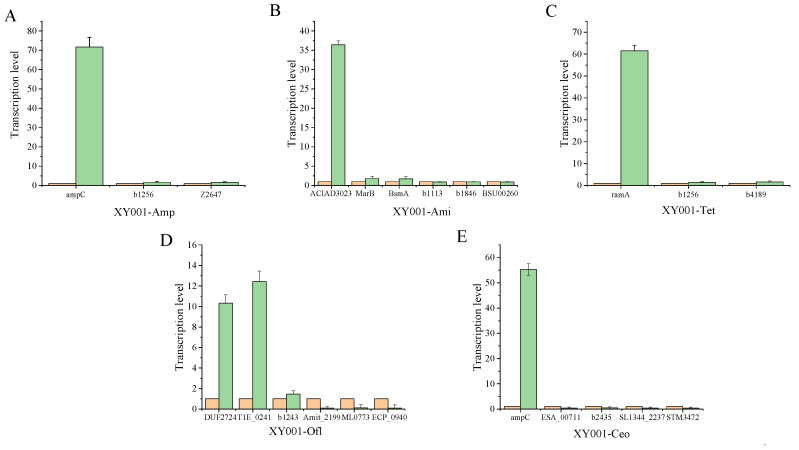
Verification of DEGs by qRT-PCR of *C. sakazakii* resistance to different classes of antibiotics. (**A**) XY001-Amp; (**B**) XY001-Ami; (**C**) XY001-Tet; (**D**) XY001-Ofl; and (**E**) XY001-Ceo.

The qRT-PCR results confirmed that the significantly upregulated genes and downregulated genes were in agreement with the previous analysis results (2^−ΔΔCt^ ≤ 0.5, *p* < 0.05), providing evidence for the correctness of the analysis. These results were consistent with the findings from the transcriptome analysis (Figure 5).

## 4. Conclusions

In this study, a strain of *C. sakazakii* susceptible to antibiotics was isolated from the environment of a Chinese infant formula processing plant. Induction of antibiotic resistance was performed using five different classes of antibiotics, including penicillins, aminoglycosides, tetracyclines, quinolones, and cephalosporins. Subsequently, the extent of cross-drug resistance and its related indicators were evaluated and analyzed.

The results revealed significant changes in the toxic factors of *C. sakazakii* induced by the aforementioned antibiotics, with quinolone antibiotics inducing strong virulence factors. Transcriptome sequencing of *C. sakazakii* induced by different classes of antibiotics was conducted using the Illumina HiSeq platform. The read rates for the XY001-Amp, XY001-Ami, XY001-Tet, XY001-Ofl, and XY001-CEO groups were 71%, 73.67%, 76.33%, 75.33%, 63.33%, and 77.67%, respectively.

Analysis of gene expression across the various groups revealed that the primary pathways implicated in the acquisition of antibiotic resistance included the β-lactamase pathway, CAMP resistance pathway, two-component system pathway, and biofilm formation pathway. This study integrated antibiotic induction, changes in resistance, virulence factors, and biofilm formation in *C. sakazakii*. It aimed to elucidate the antimicrobial resistance and degree of harm caused by *C. sakazakii* in powdered infant formula (PIF) and processing environments, thereby providing valuable support for targeted control of *C. sakazakii* drug resistance.

## Figures and Tables

**Table 1:** MIC before and after antibiotic induction of XY001.

Strain Number	Resistance Induction	Antibiotic Name				
		Amp (μg/mL)	Ami (μg/mL)	Tet (μg/mL)	Ofl (μg/mL)	Ceo (μg/mL)
XY001	Before MIC	1	8	4	0.06125	1
	After Mic	64	32	32	16	64

Note: Ami: Amikacin; Amp: ampicillin; Tet: Tetracycline; Ofl: ofloxacin; Ceo: Ceftriaxone.

**Table 2:** Antibiotic cross-resistance evaluation of XY001 after antibiotic induction.

Strain Number	Class and Name of Antibiotics											
	Penicillin			Cephalosporin					Aminoglycoside			
	Pip	Amp	Car	Cel	Cex	Ced	Ceo	Cep	Ami	Gen	Kan	Neo
XY001	S	S	S	S	S	S	S	S	S	S	S	S
XY001-Amp	R	R	R	R	R	R	I	R	R	S	S	I
XY001-Ami	I	S	S	I	I	I	I	I	R	R	R	R
XY001-Tet	R	R	R	R	R	R	I	R	R	S	S	I
XY001-Ofl	I	R	R	S	S	I	I	S	I	I	I	S
XY001-Ceo	R	R	R	R	R	R	R	R	S	S	S	S
**Strain Number**	**Tetracycline**		**Macrolide**		**Fluoroquinolone**				**Polypeptide**		**Sulfonamide**	**Lincomycin**
	**Tet**	**Dox**	**Ery**	**Mid**	**Nor**	**Ofl**	**Cip**	**Chl**	**PolB**	**Van**	**Sul**	**Lin**
XY001	S	S	R	R	S	S	S	S	S	R	S	R
XY001-Amp	I	I	R	R	S	S	S	S	I	R	I	R
XY001-Ami	S	S	R	R	S	S	S	S	S	R	R	R
XY001-Tet	R	R	R	R	S	S	S	I	S	R	R	R
XY001-Ofl	I	S	R	R	R	R	R	R	S	R	S	R
XY001-Ceo	S	S	R	R	S	S	S	S	S	R	S	R

Notes: Ami, amikacin; Amp, ampicillin; Car, carbenicillin; Cel, cefazolin; Cex, cefuroxime; Ced, ceftazidime; Ceo, ceftriaxone; Cep, cefoperazone; Chl, chloramphenicol; Cip, ciprofloxacin; Dox, doxycycline; Ery, erythromycin; Gen, gentamicin; Kan, kanamycin; Lin, lincomycin; Mid, midecamycin; Neo, neomycin; Nor, norfloxacin; Ofl, ofloxacin; Pip, piperacillin; PolB, polymyxin B; Sul, sulfamethoxazole/trimethoprim; Tet, tetracycline; Van, vancomycin; R, resistant; I, intermediate; S, susceptible.

**Table 3:** Summary of reads in *C. sakazakii* from transcriptome sequencing.

Sample	Raw Reads (M)	Raw Bases (G)	Trimmed Reads (M)	Trimmed Bases (G)	Valid Bases (%)	Q30 (%)	GC (%)
XY001	20.26 ± 2.91	3.04 ± 0.44	16.63 ± 0.76	2.46 ± 0.11	81.06 ± 7.30	92.16 ± 0.38	55.46 ± 0.74
XY001-Amp	20.47 ± 0.42	3.07 ± 0.07	16.15 ± 0.34	2.40 ± 0.05	78.14 ± 0.91	92.08 ± 0.14	55.08 ± 0.34
XY001-Ami	19.53 ± 1.37	2.93 ± 0.20	16.65 ± 0.62	2.47 ± 0.09	84.41 ± 8.38	92.10 ± 0.03	54.97 ± 0.70
XY001-Tet	19.49 ± 0.16	2.92 ± 0.03	15.96 ± 0.16	2.37 ± 0.03	81.03 ± 1.19	91.89 ± 0.15	54.84 ± 0.22
XY001-Ofl	15.62 ± 0.89	2.34 ± 0.13	15.41 ± 0.78	2.27 ± 0.13	96.71 ± 0.44	91.63 ± 0.19	54.82 ± 2.42
XY001-Ceo	18.51 ± 1.26	2.78 ± 0.19	16.78 ± 0.45	2.49 ± 0.07	89.76 ± 5.51	91.92 ± 0.23	55.71 ± 0.12

**Table 4:** Statistical results for mapping the trimmed reads with the reference genome.

Map to Genome	Total Reads	Mapped Reads	Unmapped Reads
XY001	8,188,871 (100%)	5,842,357 (71.00%)	2,346,514 (29.00%)
XY001-Amp	8,158,695 (100%)	6,020,616 (73.67%)	2,138,079 (26.33%)
XY001-Ami	8,211,781 (100%)	6,256,064 (76.33%)	1,955,717 (23.67%)
XY001-Tet	7,909,379 (100%)	5,958,875 (75.33%)	1,950,504 (24.67%)
XY001-Ofl	8,158,197 (100%)	6,327,724 (77.67%)	1,830,473 (22.33%)
XY001-Ceo	8,047,984 (100%)	5,083,396 (63.33%)	2,964,588 (36.67%)

## Data Availability

The original contributions presented in the study are included in the article/Supplementary Material, further inquiries can be directed to the corresponding author.

## Supplementary Materials

The following supporting information can be downloaded at: https://www.mdpi.com/article/10.3390/foods13172786/s1, Figure S1: Scatter diagram of KEGG annotation enrichment pathways of DEGs in *C. sakazakii* strains induced with five antibiotics; Figure S2: Histogram of GO-annotated enrichment terms of *C. sakazakii* DEGs after the induction with ampicillin; Figure S3: Histogram of GO-annotated enrichment terms of *C. sakazakii* DEGs after the induction with amikacin; Figure S4: Histogram of GO-annotated enrichment terms of *C. sakazakii* DEGs after the induction with tetracycline; Figure S5: Histogram of GO-annotated enrichment terms of *C. sakazakii* DEGs after the induction with ofloxacin; Figure S6: Histogram of GO-annotated enrichment terms of *C. sakazakii* DEGs after the induction with ceftriaxone; Table S1: The detailed information of strains; Table S2: Antibiotics for drug sensitivity determination and judgment criteria; Table S3: Antibiotic resistance results of 95 *Cronobacter* strains; Table S4: Genes related to antibiotic resistance and virulence in XY001-Amp group; Table S5: Genes related to antibiotic resistance and virulence in XY001-Ami group; Table S6: Genes related to antibiotic resistance and virulence in XY001-Tet group; Table S7: Genes related to antibiotic resistance and virulence in XY001-Ofl group; Table S8: Genes related to antibiotic resistance and virulence in XY001-Ceo group; Table S9: Gene names and information used for qRT-PCR detection.
