# Supplementary material for: Transcriptomic Analyses to Unravel Cronobacter sakazakii Resistance Pathways
Source: Foods. 2024 Sep 1;13(17):2786. doi: 10.3390/foods13172786 (PMC11394748; doi:10.3390/foods13172786)
Supplement: Supplementary file 1 [file foods-13-02786-s001.zip › foods-3123998-supplementary.pdf]

Table S1. The detailed information of strains.

Strains	Bacterial species	Area	Source
CS1	*C. sakazakii*	Northeast	PIF
CS2	*C. sakazakii*	Southern	PIF
CS4	*C. sakazakii*	Northeast	PIF
CS5	*C. sakazakii*	Northeast	PIF
CS6	*C. sakazakii*	Northeast	PIF
CS7	*C. sakazakii*	Northeast	Product of workshop
CS8	*C. sakazakii*	Northeast	PIF
CS9	*C. sakazakii*	Northeast	PIF
CS10	*C. sakazakii*	Northern	PIF
CS11	*C. sakazakii*	Northeast	PIF
CS12	*C. sakazakii*	Northern	PIF
CS13	*C. sakazakii*	Northern	PIF
CS14	*C. sakazakii*	Southern	PIF
CS15	*C. sakazakii*	Northeast	Whey powder
CS17	*C. sakazakii*	Northeast	Milk powder raw materials
CS18	*C. sakazakii*	Northeast	PIF
CS19	*C. sakazakii*	Northeast	PIF
CS20	*C. sakazakii*	Northern	PIF
CS21	*C. sakazakii*	Northern	PIF
CS25	*C. sakazakii*	Northeast	PIF
CS26	*C. sakazakii*	Northeast	PIF
CS27	*C. sakazakii*	Northeast	Milk powder raw materials
CS30	*C. sakazakii*	Northern	PIF
CS32	*C. sakazakii*	Northwest	PIF
CS33	*C. sakazakii*	Southern	Milk powder additive

CS34	*C. sakazakii*	Northeast	Milk powder additive
CS35	*C. sakazakii*	Northeast	PIF
CS37	*C. sakazakii*	Northeast	PIF
CS39	*C. sakazakii*	Northeast	PIF
CS40	*C. sakazakii*	Northern	PIF
CS41	*C. sakazakii*	Northeast	PIF
CS42	*C. sakazakii*	Northeast	PIF
CS43	*C. sakazakii*	Eastern	PIF
CS44	*C. sakazakii*	Eastern	PIF
CS46	*C. sakazakii*	Northern	PIF
CS47	*C. sakazakii*	Northern	PIF
CS48	*C. sakazakii*	Northern	PIF
CS49	*C. sakazakii*	Northern	PIF
CS50	*C. sakazakii*	Northern	PIF
CS51	*C. sakazakii*	Northern	PIF
CS52	*C. sakazakii*	Northern	PIF
CS54	*C. sakazakii*	Northern	PIF
CS55	*C. sakazakii*	Northern	PIF
CS56	*C. sakazakii*	Northeast	PIF
CS57	*C. sakazakii*	Northeast	PIF
CS59	*C. sakazakii*	Northeast	PIF
CS60	*C. sakazakii*	Northeast	PIF
CS62	*C. sakazakii*	Northeast	Product of workshop
CS64	*C. sakazakii*	Northeast	Product of workshop
CS65	*C. sakazakii*	Northeast	Milk powder additive
CS66	*C. sakazakii*	Northeast	PIF
CS68	*C. sakazakii*	Northeast	Fluidized bed powder block
XY001	*C. sakazakii*	Northeast	PIF

CS70	*C. sakazakii*	Northeast	PIF
CS71	*C. sakazakii*	Northeast	PIF
CS72	*C. sakazakii*	Northeast	PIF
CS73	*C. sakazakii*	Northeast	PIF
CM73	*C. sakazakii*	Northeast	PIF
CS74	*C. sakazakii*	Northeast	PIF
CS75	*C. sakazakii*	Northeast	PIF
CS77	*C. sakazakii*	Northeast	PIF
CS78	*C. sakazakii*	Northeast	PIF
CS80	*C. sakazakii*	Northeast	PIF
CS81	*C. sakazakii*	Northeast	PIF
CS82	*C. sakazakii*	Northeast	PIF
CS83	*C. sakazakii*	Northeast	Milk powder additive
CS85	*C. sakazakii*	Northeast	U-type valve
CS86	*C. sakazakii*	Northeast	Product of workshop
CS87	*C. sakazakii*	Northeast	Fluidized bed powder block
CS88	*C. sakazakii*	Northeast	Spray dried powder
CS89	*C. sakazakii*	Northeast	Product of workshop
CS90	*C. sakazakii*	Northeast	Fixed bed
CS91	*C. sakazakii*	Northeast	Fixed bed
CS93	*C. sakazakii*	Northeast	Fluidized bed powder block
CS94	*C. sakazakii*	Northeast	PIF
CS95	*C. sakazakii*	Northeast	Product of workshop
CS96	*C. sakazakii*	Northeast	Product of workshop
CS97	*C. sakazakii*	Northeast	Product of workshop
CS99	*C. sakazakii*	Northeast	Fluidized bed powder block

CS100	*C. sakazakii*	Northeast	Product of workshop
CS101	*C. sakazakii*	Northeast	Product of workshop
CS102	*C. sakazakii*	Northeast	Product of workshop
CS104	*C. sakazakii*	Northeast	Fluidized bed powder block
FP-40	*C. sakazakii*	Northeast	PIF

Table S2. Antibiotics for drug sensitivity determination and judgment criteria.

**Antimicrobial Group**	**Antibiotics**	**Content (μg/tablet)**	**Diameter of Inhibition Zone (mm)**		
			**Resistance**	**Intermediary**	**Sensitivity**
**Penicillin**					
	Ampicillin	100	≤17	18-20	≥21
	Piperacillin	10	≤13	14-16	≥17
	Carbenicillin	100	≤19	20-22	≥23
**Cephalosporin**					
	Cefazolin	30	≤14	15-17	≥18
	Cefuroxime	30	≤14	15-17	≥18
	Ceftazidime	30	≤17	18-20	≥21
	Ceftriaxone	30	≤19	20-22	≥23
	Cefoperazone	75	≤15	16-20	≥21
**Aminoglycoside**					
	Amikacin	30	≤14	15-16	≥17
	Gentamicin	10	≤12	13-14	≥15
	Kanamycin	30	≤13	14-17	≥18
	Neomycin	30	≤12	13-16	≥17
**Tetracycline**					
	Tetracycline	30	≤11	12-14	≥15
	Doxycycline	30	≤10	11-13	≥14
**Macrolide**					
	Erythromycin	15	≤13	14-22	≥23
	Midecamycin	15	≤13	14-22	≥23
**Fluoroquinolone**					
	Norfloxacin	10	≤12	13-16	≥17
	Ofloxacin	5	≤12	13-15	≥16
	Ciprofloxacin	5	≤15	16-20	≥21
	Chloramphenicol	30	≤12	13-17	≥18
**Polypeptide**					
	Polymyxin B	300	≤8	8-11	≥12
	Vancomycin	30	≤14	15-16	≥17
**Sulfonamide**					

	Sulfamethoxazole/ Trimethoprim	23.75/1.25	≤10	11-15	≥16
**Lincomycin**					
	Lincomycin	30	≤12	13-17	≥18

Table S3. Antibiotic resistance results of 95 *Cronobacter* strains.

Strain Number	Class and Name of Antibiotics																						
	**Penicillin**				**Cephalosporin**				**Aminoglycoside**				**Tetracycline**		**Macrolide**		**Fluoroquinolone**				**Polypeptide**		**Sulfonamide**	**Lincomycin**
	Pip	Amp	Car	Cel	Cex	Ced	Ceo	Cep	Ami	Gen	Kan	Neo	Tet	Dox	Ery	Mid	Nor	Ofl	Cip	Chl	PolB	Van	Sul	Lin
CS1	R	S	R	S	S	S	S	S	R	S	R	R	S	R	R	R	S	S	S	S	S	R	I	R
CS2	S	S	R	I	S	S	S	S	S	R	S	I	S	R	R	R	S	S	S	S	S	R	I	R
CS3	S	R	S	S	S	S	S	R	S	S	S	R	S	S	R	R	S	S	S	S	S	R	I	R
CS4	S	R	I	R	S	I	I	I	S	S	I	I	S	S	R	R	S	S	S	S	I	R	I	R
CS5	S	I	I	I	R	S	I	I	S	S	S	I	R	R	R	R	I	S	S	I	R	R	R	R
CS7	S	S	I	S	S	S	S	S	R	S	I	S	S	S	R	R	S	S	S	S	S	R	S	R
CS8	S	S	I	R	S	S	I	I	S	S	I	I	S	S	R	R	S	S	S	S	S	R	I	R
CS9	S	I	I	S	S	S	S	S	S	S	S	I	S	S	R	R	S	S	S	S	S	R	S	R
CS10	S	S	I	I	S	S	S	S	S	S	R	R	S	S	R	R	S	S	S	R	R	R	R	R
CS11	S	S	R	S	S	S	S	I	S	S	S	I	S	S	R	R	S	S	S	I	S	R	S	R
CS12	S	S	I	S	S	S	S	S	S	R	R	I	S	S	R	R	S	S	S	S	S	R	I	R
CS13	S	S	S	R	S	S	I	I	S	S	S	I	S	S	R	R	S	S	S	S	I	R	I	R
CS14	R	R	R	R	R	R	R	R	R	R	R	R	S	S	R	R	S	S	S	S	R	R	R	R
CS15	S	S	I	I	S	S	S	S	S	I	I	R	S	S	R	R	S	S	S	S	S	R	I	R
CS16	S	S	I	R	S	S	S	S	S	S	S	I	S	S	R	R	S	S	S	S	S	R	I	R
CS17	R	R	R	R	R	R	R	R	S	S	S	S	S	S	R	R	S	S	S	S	R	R	R	R
CS18	S	S	I	S	S	S	S	S	I	S	I	I	I	R	R	R	S	S	S	S	S	R	I	R
CS19	S	S	I	R	S	S	S	S	I	I	I	I	S	S	R	R	S	S	S	S	I	R	I	R
CS20	S	S	I	S	S	I	R	I	R	I	R	R	S	S	R	R	S	S	S	S	S	R	I	R
CS21	S	S	I	I	S	S	S	S	I	S	R	I	S	S	R	R	S	S	S	S	S	R	I	R
CS22	I	S	I	I	S	S	S	I	R	I	R	I	S	S	R	R	S	S	S	S	I	R	R	R

CS23	S	S	I	S	S	R	R	S	R	I	I	R	S	S	R	R	R	I	S	I	S	R	I	R
CS24	S	S	S	R	R	R	R	R	R	R	R	R	S	S	R	R	S	S	S	S	I	R	R	R
CS25	S	S	R	I	S	R	I	I	I	R	R	R	S	S	R	R	R	I	S	I	S	R	S	R
CS26	S	S	I	S	S	S	S	I	S	S	S	I	S	S	R	R	S	S	S	S	S	R	S	R
CS27	I	R	R	S	R	R	R	I	S	S	I	I	I	S	R	R	S	S	S	S	R	R	R	R
CS28	S	S	I	S	S	S	S	I	I	S	I	R	R	S	R	R	I	S	S	S	I	R	R	R
CS29	I	R	I	I	R	R	R	I	I	S	I	R	S	S	R	R	S	S	S	S	I	R	I	R
CS30	S	S	I	R	S	S	S	I	R	S	R	I	S	S	R	R	R	S	S	S	S	R	I	R
CS31	S	S	I	R	S	S	S	S	R	S	R	R	S	S	R	R	S	S	S	S	S	R	I	R
CS32	S	S	I	S	I	S	R	S	R	S	I	I	S	S	R	R	I	S	S	S	S	R	I	R
CS33	S	S	S	I	S	R	R	I	I	S	I	I	S	S	R	R	I	S	S	S	S	R	I	R
CS34	S	I	S	I	S	S	S	R	R	R	I	R	S	S	R	R	I	S	S	S	S	R	I	R
CS35	S	I	S	S	S	S	S	I	I	I	I	R	S	S	R	R	S	S	S	S	R	R	I	R
CS36	R	I	S	S	S	S	S	R	R	R	I	R	S	S	R	R	S	S	I	S	I	R	I	R
CS37	S	S	S	R	R	S	S	I	R	I	I	R	S	S	R	R	S	S	S	S	I	R	I	R
CS37	S	I	R	I	S	S	S	I	R	I	I	R	R	S	R	R	S	S	S	S	S	R	I	R
CS38	S	S	S	I	S	S	S	I	R	R	R	R	I	S	R	R	S	S	S	S	I	R	I	R
CS39	I	I	S	R	S	S	S	I	R	I	R	R	S	S	R	R	S	S	I	S	I	R	I	R
CS40	I	I	S	I	S	S	S	I	R	R	R	R	S	S	R	R	S	S	I	S	S	R	S	R
CS41	S	I	S	S	S	S	S	I	R	R	I	R	S	S	R	R	S	S	S	S	I	R	S	R
CS42	R	I	S	R	S	S	S	I	R	R	R	R	S	S	R	R	S	S	I	S	S	R	I	R
CS43	I	I	S	S	S	S	S	I	R	R	R	R	S	S	R	R	S	S	I	S	I	R	I	R
CS44	S	I	R	S	S	S	S	I	R	I	I	R	S	S	R	R	S	S	I	S	S	R	I	R
CS46	I	R	S	R	I	S	S	R	R	I	I	R	S	S	R	R	S	S	S	S	I	R	I	R
CS47	I	I	S	R	I	S	S	I	R	I	R	R	S	S	R	R	S	S	S	S	I	R	R	R
CS48	I	R	S	R	S	S	S	I	R	R	I	R	S	S	R	R	I	S	S	S	R	R	I	R

CS49	I	I	S	R	R	I	S	I	R	R	R	R	S	S	R	R	S	S	S	S	I	R	I	R
CS50	I	I	R	S	S	S	S	I	R	S	I	R	S	S	R	R	S	S	S	S	I	R	S	R
CS51	I	R	S	S	I	S	S	S	R	I	R	R	S	S	R	R	S	S	S	S	I	R	S	R
CS52	R	I	S	R	S	S	S	I	R	I	R	R	S	S	R	R	S	S	I	S	S	R	I	R
CS54	I	R	S	R	I	S	S	I	R	I	I	R	S	S	R	R	S	S	S	S	S	R	S	R
CS55	R	I	I	R	I	S	S	R	R	R	I	R	S	S	R	R	S	S	S	S	I	R	I	R
CS56	R	I	S	S	S	S	S	I	R	I	R	R	S	S	R	R	S	S	S	S	I	R	I	R
CS57	I	I	R	S	S	S	S	I	R	R	I	R	S	I	R	R	S	S	S	S	S	R	I	R
CS59	I	I	S	R	S	S	S	I	I	I	I	I	S	S	R	R	S	S	S	S	I	R	I	R
CS60	I	I	S	R	S	S	S	I	R	I	I	R	S	S	R	R	S	S	S	S	I	R	S	R
CS62	S	S	S	S	S	S	S	S	R	R	R	R	S	S	R	R	S	S	S	S	S	R	S	R
CS64	R	R	S	R	S	S	S	I	I	I	I	R	S	S	R	R	S	S	S	S	I	R	I	R
CS65	I	I	S	R	S	S	S	S	R	R	I	R	S	S	R	R	S	S	S	S	I	R	S	R
CS66	I	R	I	S	S	S	S	S	I	I	I	I	S	S	R	R	S	S	S	S	I	R	I	R
CS67	R	R	R	S	S	S	S	S	S	S	S	S	S	S	R	R	S	S	S	S	S	R	I	R
CS68	I	I	S	S	S	S	S	S	I	I	I	R	S	S	R	R	S	S	S	S	S	R	S	R
XY001	S	S	S	S	S	S	S	S	S	S	S	S	S	S	R	R	S	S	S	S	S	R	S	R
CS70	I	S	S	I	S	S	S	S	I	S	S	R	S	S	R	R	I	S	I	S	I	R	I	R
CS71	I	I	R	I	I	S	S	I	S	I	I	R	S	S	R	R	S	S	S	S	S	R	I	R
CS72	I	S	R	I	S	S	S	S	I	I	I	R	S	S	R	R	S	S	S	S	S	R	I	R
CS73	I	S	I	I	S	S	S	S	I	I	I	R	S	S	R	R	S	S	S	S	S	R	I	R
CS73	I	I	S	S	S	S	S	S	R	I	I	R	S	S	R	R	S	S	S	S	I	R	I	R
CS74	I	R	I	S	I	S	S	S	I	R	I	R	S	S	R	R	S	S	I	S	S	R	S	R
CS75	S	R	I	I	I	S	S	I	R	I	I	R	S	S	R	R	S	S	S	S	S	R	R	R
CS77	I	R	S	R	S	S	S	I	R	I	I	I	S	S	R	R	S	S	S	S	S	R	I	R
CS78	S	R	S	S	S	S	S	I	I	I	I	R	S	S	R	R	S	S	S	S	I	R	I	R

CS80	S	R	R	R	S	S	S	I	I	I	I	R	S	S	R	R	I	S	S	S	I	R	I	R
CS81	I	I	S	I	S	S	S	I	R	S	I	I	S	S	R	R	S	S	S	S	S	R	S	R
CS82	I	R	I	I	S	S	S	S	I	I	I	R	S	S	R	R	S	S	S	S	S	R	I	R
CS83	S	S	S	I	S	S	S	I	R	I	I	R	S	S	R	R	S	S	S	S	S	R	I	R
CS85	S	R	R	R	I	S	S	I	I	I	I	R	S	S	R	R	S	S	S	S	S	R	S	R
CS86	I	I	R	R	S	S	S	I	R	I	I	R	S	S	R	R	S	S	S	S	S	R	S	R
CS87	I	R	I	S	S	S	S	S	R	I	R	R	S	S	R	R	S	S	S	S	I	R	I	R
CS88	I	R	I	I	S	S	S	I	I	I	I	R	S	S	R	R	S	S	S	S	S	R	I	R
CS89	S	I	S	S	S	S	S	S	R	S	I	R	S	S	R	R	S	S	S	S	S	R	S	R
CS90	S	I	S	S	I	S	S	S	R	I	R	R	S	S	R	R	S	S	S	S	S	R	S	R
CS91	S	I	I	R	I	S	S	I	R	I	I	R	S	S	R	R	S	S	S	S	I	R	S	R
CS93	I	R	S	S	S	S	S	S	R	S	I	R	S	S	R	R	S	S	S	S	I	R	S	R
CS94	S	S	I	I	S	S	S	S	R	I	I	R	S	S	R	R	S	S	S	S	S	R	S	R
CS95	S	R	S	R	S	S	S	I	R	R	I	R	S	S	R	R	S	S	S	S	S	R	S	R
CS96	S	I	S	I	S	S	S	I	R	I	I	I	S	S	R	R	S	S	I	S	I	R	S	R
CS97	S	S	I	R	S	S	S	S	R	S	I	R	S	S	R	R	S	S	S	S	S	R	I	R
CS99	I	R	S	R	S	S	S	S	R	I	I	R	S	S	R	R	S	S	I	S	S	R	I	R
CS100	S	S	S	S	S	S	S	S	R	R	R	R	S	S	R	R	S	S	S	S	S	R	S	R
CS101	S	S	S	S	S	S	S	I	R	I	S	I	S	S	R	R	S	S	S	S	S	R	S	R
CS102	S	S	S	R	S	S	S	S	R	I	I	R	S	S	R	R	R	S	S	S	S	R	I	R
CS104	S	S	S	S	S	S	S	I	I	S	I	I	S	S	R	R	S	S	S	S	S	R	I	R
FP-40	R	R	I	R	S	S	S	S	I	I	R	R	S	S	R	R	S	S	S	S	S	R	S	R

Pip: Piperacillin; Amp: Ampicillin; Car: Carbenicillin; Cel: Cefazolin; Cex: Cefuroxime; Ced: Ceftazidime; Ceo: Ceftriaxone; Cep: Cefoperazone; Ami: Amikacin; Gen: Gentamicin; Kan: Kanamycin; Neo: Neomycin; Tet: Tetracycline; Dox: Doxycycline; Ery: Erythromycin; Mid: Midecamycin; Nor: Norfloxacin; Ofl: Ofloxacin; Cip: Ciprofloxacin; Chl: Chloramphenicol; PolB: Polymyxin B; Van: Vancomycin; Sul: Sulfamethoxazole/Trimethoprim; Lin: Lincomycin. **R**: resistant; **I**: intermediate; **S**: susceptible.

Table S4. Genes related to antibiotic resistance and virulence in XY001-Amp group.

	Gene id	Gene	p-Value	q-Value	log_2Foldchange	Description	Pathway Description
Genes associated with antibiotic resistance	CsakCS931_RS00535	SM_b21465	0.012025	0.04473	-0.92	MULTISPECIES: HlyD family efflux transporter periplasmic adaptor subunit	/
	CsakCS931_RS02150	b2301	0.721905	0.802467	0.11	glutathione transferase	Glutathione metabolism ; Metabolism of xenobiotics by cytochrome P450 ; Drug metabolism - cytochrome P450 ; Drug metabolism - other enzymes ; Platinum drug resistance ; Longevity regulating pathway - worm ; Pathways in cancer ; Chemical carcinogenesis ; Hepatocellular carcinoma ; Fluid shear stress and atherosclerosis
	CsakCS931_RS02170	ramA	3.27E-64	8.01E-61	5.16	MULTISPECIES: RamA family antibiotic efflux transcriptional regulator	/
	CsakCS931_RS02425	SL1344_2237	0.00617	0.028924	0.74	MULTISPECIES: porin OmpC	beta-Lactam resistance ; Two-component system
	CsakCS931_RS03415	b0208	0.100354	0.189454	0.39	LysR family transcriptional regulator	/

CsakCS931_RS05485	T1E_0241	0.072669	0.148769	0.52	efflux transporter outer membrane subunit	beta-Lactam resistance ; Quorum sensing
CsakCS931_RS05905	Z2170	0.595203	0.697505	-0.16	MULTISPECIES: MDR efflux pump AcrAB transcriptional activator MarA	Cationic antimicrobial peptide (CAMP) resistance
CsakCS931_RS05935	b1526	5.52E-15	4.23E-13	1.55	LysR family transcriptional regulator	/
CsakCS931_RS06210	ampC	######	######	5.51	class C beta-lactamase CSA-1	beta-Lactam resistance ; Two-component system
CsakCS931_RS02545	b2180	0.055557	0.122436	-0.58	microcin C ABC transporter ATP-binding protein YejF	ABC transporters
CsakCS931_RS06735	Z2647	1.04E-05	0.000164	1.57	MULTISPECIES: glutathione transferase GstA	Glutathione metabolism ; Metabolism of xenobiotics by cytochrome P450 ; Drug metabolism - cytochrome P450 ; Drug metabolism - other enzymes ; Platinum drug resistance ; Longevity regulating pathway - worm ; Pathways in cancer ; Chemical carcinogenesis ; Hepatocellular carcinoma ; Fluid shear stress and atherosclerosis

CsakCS931_RS06780	b1644	0.432915	0.553892	0.23	HlyD family secretion protein	/
CsakCS931_RS11615	HI_1364	0.015546	0.051613	-1.52	LysR family transcriptional regulator	/
CsakCS931_RS08350	ESA_02283	1.93E-05	0.000282	0.98	MULTISPECIES: multidrug efflux MFS transporter MdtH	/
CsakCS931_RS08895	SCH_0941	0.174604	0.285414	0.45	MULTISPECIES: lipid A ABC transporter ATP-binding protein/permease MsbA	ABC transporters
CsakCS931_RS08990	b0887	0.82477	0.882794	-0.06	transport ATP-binding protein CydD	ABC transporters
CsakCS931_RS08995	b0886	0.345783	0.468834	-0.30	cysteine/glutathione ABC transporter ATP-binding protein/permease CydC	ABC transporters
CsakCS931_RS09230	c0923	9.61E-11	4.13E-09	1.21	MULTISPECIES: glutathione S-transferase family protein	Glutathione metabolism ; Metabolism of xenobiotics by cytochrome P450 ; Drug metabolism - cytochrome P450 ; Drug metabolism - other enzymes ; Platinum drug resistance ; Longevity regulating pathway - worm ; Pathways in cancer ; Chemical carcinogenesis ;

CsakCS931_RS10760	STM0498	0.001596	0.011372	0.55	copper-exporting P-type ATPase CopA	Hepatocellular carcinoma ; Fluid shear stress and atherosclerosis Platinum drug resistance ; MAPK signaling pathway - plant
CsakCS931_RS12020	b0208	0.024829	0.070636	0.44	LysR family transcriptional regulator	/
CsakCS931_RS12245	Z0173	0.436928	0.556897	0.22	MULTISPECIES: serine endoprotease DegP	Cationic antimicrobial peptide (CAMP) resistance ; Two-component system
CsakCS931_RS12845	ltrA	0.644457	0.737318	-0.24	LysR family transcriptional regulator	/
CsakCS931_RS06615	PA2258	0.156902	0.263512	0.29	LysR family transcriptional regulator	/
CsakCS931_RS13350	b3597	0.030501	0.082057	-1.17	MULTISPECIES: HlyD family secretion protein	/
CsakCS931_RS02980	ESA_01152	0.578521	0.683098	-0.19	MULTISPECIES: uridine kinase	Pyrimidine metabolism ; Drug metabolism - other enzymes
CsakCS931_RS01095	ESA_00755	0.009913	0.039357	-0.69	MULTISPECIES: glutamine-hydrolyzing GMP synthase	Purine metabolism ; Drug metabolism - other enzymes
CsakCS931_RS00030	ESA_00523	0.814837	0.876395	0.08	MULTISPECIES: phosphopyruvate hydratase	Glycolysis / Gluconeogenesis ; Methane metabolism ; RNA degradation ; HIF-1 signaling pathway

CsakCS931_RS14335	c3998	0.068643	0.142613	0.40	MULTISPECIES: HTH-type transcriptional activator AaeR	/
CsakCS931_RS15870	b3673	0.151345	0.256642	-0.40	multidrug resistance protein D	/
CsakCS931_RS16085	SBO_3657	0.132356	0.23434	0.35	MULTISPECIES: phosphate ABC transporter ATP-binding protein PstB	ABC transporters
CsakCS931_RS16295	STM3997	0.07241	0.148485	0.44	MULTISPECIES: thiol:disulfide interchange protein DsbA	Cationic antimicrobial peptide (CAMP) resistance
CsakCS931_RS16685	Z5457	0.480489	0.596683	0.18	MULTISPECIES: envelope stress response regulator transcription factor CpxR	Cationic antimicrobial peptide (CAMP) resistance ; Two-component system
CsakCS931_RS17040	b3540	0.019028	0.059043	-0.91	MULTISPECIES: dipeptide ABC transporter ATP binding subunit DppF	ABC transporters
CsakCS931_RS17130	b3521	0.378433	0.500795	-0.30	MULTISPECIES: LysR family transcriptional regulator	/
CsakCS931_RS18435	gstA	1.10E-06	2.18E-05	0.82	glutathione S-transferase	Glutathione metabolism ; Metabolism of xenobiotics by cytochrome P450 ; Drug

							metabolism - cytochrome P450 ; Drug metabolism - other enzymes ; Platinum drug resistance ; Longevity regulating pathway - worm ; Pathways in cancer ; Chemical carcinogenesis ; Hepatocellular carcinoma ; Fluid shear stress and atherosclerosis
	CsakCS931_RS19100	b0900	0.799313	0.86385	-0.15	LysR family transcriptional regulator	/
Genes associated with bacterial virulence	CsakCS931_RS06360	/	0.042162	0.104012	0.44	virulence factor SrfB	/
	CsakCS931_RS06365	/	0.120112	0.221352	-0.80	virulence factor	/
	CsakCS931_RS07610	b0877	0.010296	0.041232	0.94	Virulence factor VirK	/
	CsakCS931_RS15765	PA0086	0.017409	0.057218	-1.07	protein of avirulence locus ImpE	/
	CsakCS931_RS16410	ESA_04062	0.203138	0.323326	-0.38	virulence factor BrkB family protein	/
Genes associated with Biofilms	CsakCS931_RS00150	STM2924	0.003128	0.018888	-0.92	MULTISPECIES: RNA polymerase sigma factor RpoS	Biofilm formation - Escherichia coli; Biofilm formation - Vibrio cholerae
	CsakCS931_RS00250	ECDH10B_2864	0.021737	0.066169	-0.87	MULTISPECIES: carbon storage regulator CsrA	Two-component system; Biofilm formation -

						Pseudomonas aeruginosa; Biofilm formation - Escherichia coli; Biofilm formation - Vibrio cholerae
CsakCS931_RS00300	ESA_00581	1.09E-05	0.000181	-1.13	MULTISPECIES: S-ribosylhomocysteine lyase	Cysteine and methionine metabolism; Quorum sensing; Biofilm formation - Escherichia coli; Biofilm formation - Vibrio cholerae
CsakCS931_RS01175	b2479	2.73E-07	6.43E-06	-1.00	MULTISPECIES: glycine cleavage system transcriptional repressor	Biofilm formation - Escherichia coli
CsakCS931_RS01455	STM2433	0.185695	0.302917	-0.50	MULTISPECIES: PTS glucose transporter subunit IIA	Glycolysis / Gluconeogenesis; Starch and sucrose metabolism; Amino sugar and nucleotide sugar metabolism; Biofilm formation - Escherichia coli; Phosphotransferase system (PTS); Biofilm formation - Vibrio cholerae
CsakCS931_RS02410	STY2496	0.338171	0.465931	-0.18	Sensor kinase protein RcsC	Two-component system; Biofilm formation - Escherichia coli

CsakCS931_RS02415	SF2301	0.038721	0.098357	-0.62	MULTISPECIES: transcriptional regulator RcsB	Two-component system; Biofilm formation - Escherichia coli
CsakCS931_RS02420	b2216	0.010398	0.041413	-0.74	Sensor-like histidine kinase RcsD	Two-component system; Biofilm formation - Escherichia coli
CsakCS931_RS02805	b2127	0.966887	0.980881	0.01	MULTISPECIES: MerR family transcriptional regulator	Biofilm formation - Escherichia coli
CsakCS931_RS02975	b2067	2.68E-05	0.000388	-0.73	diguanylate cyclase	Biofilm formation - Escherichia coli
CsakCS931_RS03000	STM2118	0.002482	0.016046	1.15	MULTISPECIES: polysaccharide export protein	Two-component system; Biofilm formation - Escherichia coli
CsakCS931_RS03075	b2047	0.62491	0.724833	0.37	MULTISPECIES: undecaprenyl-phosphate glucose phosphotransferase	Biofilm formation - Vibrio cholerae
CsakCS931_RS03470	SSON_2013	0.005797	0.028395	-0.58	cellulose biosynthesis regulator YedQ	Biofilm formation - Escherichia coli
CsakCS931_RS03490	rcsA	0.990138	0.996205	0.00	MULTISPECIES: transcriptional regulator RcsA	Two-component system; Quorum sensing; Biofilm formation - Escherichia coli
CsakCS931_RS03700	STM1956	0.220682	0.34199	0.30	MULTISPECIES: RNA polymerase sigma factor FliA	Two-component system; Biofilm formation - Pseudomonas aeruginosa;

						Biofilm formation - Escherichia coli; Flagellar assembly; Biofilm formation - Vibrio cholerae
CsakCS931_RS03705	STY2163	0.945731	0.966283	0.03	MULTISPECIES: flagella biosynthesis regulatory protein FliZ	Biofilm formation - Escherichia coli
CsakCS931_RS03730	b1916	0.495067	0.610801	-0.25	MULTISPECIES: transcriptional regulator SdiA	Two-component system; Quorum sensing; Biofilm formation - Escherichia coli
CsakCS931_RS03740	SF1957	0.010207	0.041052	-0.91	MULTISPECIES: UvrY/SirA/GacA family response regulator transcription factor	Two-component system; Biofilm formation - Pseudomonas aeruginosa; Biofilm formation - Escherichia coli; Biofilm formation - Vibrio cholerae
CsakCS931_RS03870	ESA_01337	0.000538	0.005023	-1.00	Flagellar transcriptional activator FlhD	Two-component system; Quorum sensing; Biofilm formation - Escherichia coli; Flagellar assembly
CsakCS931_RS03875	STM1924	0.0052	0.026259	-0.85	MULTISPECIES: flagellar transcriptional regulator FlhC	Two-component system; Quorum sensing; Biofilm formation - Escherichia coli; Flagellar assembly
CsakCS931_RS04310	b1815	0.772225	0.84456	0.06	EAL domain-containing protein	Biofilm formation - Escherichia coli

CsakCS931_RS04570	STM1798	0.002145	0.01449	-0.59	MULTISPECIES: flagellar brake protein YcgR	Biofilm formation - Escherichia coli
CsakCS931_RS05000	STM1723	0.64046	0.737043	-0.17	anthranilate synthase subunit I	Phenylalanine, tyrosine and tryptophan biosynthesis; Phenazine biosynthesis; Quorum sensing; Biofilm formation - Pseudomonas aeruginosa
CsakCS931_RS05130	b1285	0.000929	0.007773	-0.74	cyclic di-GMP phosphodiesterase	Quorum sensing; Biofilm formation - Escherichia coli
CsakCS931_RS05965	c0492	0.750367	0.827199	0.09	sensor domain-containing diguanylate cyclase	Biofilm formation - Escherichia coli
CsakCS931_RS06940	PA0083	0.000168	0.001883	2.06	MULTISPECIES: type VI secretion system contractile sheath small subunit	Biofilm formation - Pseudomonas aeruginosa
CsakCS931_RS08160	b1101	0.272633	0.399845	0.40	MULTISPECIES: PTS glucose transporter subunit IIBC	Glycolysis / Gluconeogenesis; Amino sugar and nucleotide sugar metabolism; Phosphotransferase system (PTS); Biofilm formation - Vibrio cholerae
CsakCS931_RS08320	STM1172	0.158884	0.270394	0.46	MULTISPECIES: anti-sigma-28 factor FlgM	Two-component system; Biofilm formation - Pseudomonas aeruginosa;

						Biofilm formation - Escherichia coli; Flagellar assembly
CsakCS931_RS11590	VC_0137	0.171671	0.286795	0.26	EAL domain-containing protein	Biofilm formation - Vibrio cholerae
CsakCS931_RS12325	SF0137	0.162215	0.274509	-0.47	DnaK suppressor protein	Biofilm formation - Escherichia coli
CsakCS931_RS13000	Z6004	0.034329	0.090613	-0.64	MULTISPECIES: two-component system response regulator ArcA	Two-component system; Biofilm formation - Escherichia coli
CsakCS931_RS14160	ntrA	0.016215	0.054447	-0.63	MULTISPECIES: RNA polymerase factor sigma-54	Two-component system; Biofilm formation - Vibrio cholerae
CsakCS931_RS14195	SF3250	0.142516	0.251266	-0.26	aerobic respiration two-component sensor histidine kinase ArcB	Two-component system; Biofilm formation - Escherichia coli
CsakCS931_RS14435	b3261	0.002853	0.01787	1.39	MULTISPECIES: DNA-binding transcriptional regulator Fis	Biofilm formation - Vibrio cholerae
CsakCS931_RS14925	STY3620	0.004366	0.023452	-0.89	class I adenylate cyclase	Purine metabolism; Biofilm formation - Escherichia coli; Biofilm formation - Vibrio cholerae
CsakCS931_RS15010	b3787	0.141018	0.249971	0.33	MULTISPECIES: UDP-N-acetyl-D-mannosamine dehydrogenase	Amino sugar and nucleotide sugar metabolism; Two-

						component system; Biofilm formation - Vibrio cholerae
CsakCS931_RS15015	b3786	0.586902	0.692209	-0.17	MULTISPECIES: UDP-N-acetylglucosamine 2-epimerase (non-hydrolyzing)	Amino sugar and nucleotide sugar metabolism; Two-component system; Biofilm formation - Vibrio cholerae
CsakCS931_RS15180	b3961	0.01444	0.050876	-0.84	MULTISPECIES: DNA-binding transcriptional regulator OxyR	Biofilm formation - Escherichia coli
CsakCS931_RS15740	PA0090	0.00523	0.026352	1.13	type VI secretion system ATPase TssH	Biofilm formation - Pseudomonas aeruginosa; Bacterial secretion system
CsakCS931_RS15750		0.004092	0.022699	1.13	MULTISPECIES: type VI secretion system baseplate subunit TssG	Biofilm formation - Pseudomonas aeruginosa
CsakCS931_RS15795	PA0085	0.0046	0.024047	1.08	MULTISPECIES: type VI secretion system tube protein Hcp	Biofilm formation - Pseudomonas aeruginosa; Bacterial secretion system
CsakCS931_RS15815	PA0084	0.00084	0.007198	0.95	MULTISPECIES: type VI secretion system contractile sheath large subunit	Biofilm formation - Pseudomonas aeruginosa
CsakCS931_RS15825	PA0082	0.0351	0.0921	0.57	type VI secretion system protein TssA	Biofilm formation - Pseudomonas aeruginosa

CsakCS931_RS15830		0.002744	0.017412	1.21	MULTISPECIES: type VI secretion system-associated protein TagF	Biofilm formation - Pseudomonas aeruginosa
CsakCS931_RS15835	PA0077	0.000184	0.002031	1.04	MULTISPECIES: type VI secretion system	Biofilm formation - Pseudomonas aeruginosa; Bacterial secretion system
CsakCS931_RS15845	PA0079	0.000102	0.001239	1.49	MULTISPECIES: type VI secretion system baseplate subunit TssK	Biofilm formation - Pseudomonas aeruginosa
CsakCS931_RS16670	STM3699	0.900113	0.938275	0.04	serine acetyltransferase	Cysteine and methionine metabolism; Sulfur metabolism; Biofilm formation - Vibrio cholerae
CsakCS931_RS16805	PA5267	0.263496	0.390095	1.30	type VI secretion system tube protein Hcp	Biofilm formation - Pseudomonas aeruginosa; Bacterial secretion system
CsakCS931_RS17070	b3533	0.164451	0.277285	0.35	UDP-forming cellulose synthase catalytic subunit	Starch and sucrose metabolism; Biofilm formation - Escherichia coli
CsakCS931_RS17110	b3525	0.035434	0.092655	0.95	cyclic-guanylate-specific phosphodiesterase	Biofilm formation - Escherichia coli
CsakCS931_RS17460	ESA_04311	0.062872	0.136356	-0.48	MULTISPECIES: glucose-1-phosphate adenylyltransferase	Starch and sucrose metabolism; Amino sugar and nucleotide sugar metabolism; Biofilm formation - Escherichia coli

CsakCS931_RS17465	ESA_04312	0.357936	0.485682	-0.28	MULTISPECIES: glycogen synthase GlgA	Starch and sucrose metabolism; Biofilm formation - Escherichia coli
CsakCS931_RS17470	SF3451	0.358419	0.486066	-0.23	MULTISPECIES: glycogen phosphorylase	Starch and sucrose metabolism; Biofilm formation - Escherichia coli; Necroptosis; Insulin signaling pathway; Glucagon signaling pathway; Insulin resistance
CsakCS931_RS17510	b3417	0.245483	0.370106	0.25	maltodextrin phosphorylase	Starch and sucrose metabolism; Biofilm formation - Escherichia coli; Necroptosis; Insulin signaling pathway; Glucagon signaling pathway; Insulin resistance
CsakCS931_RS17570	Z4760	2.81E-09	1.03E-07	-1.28	hypothetical protein ESA_04334	Two-component system; Biofilm formation - Escherichia coli
CsakCS931_RS17575	b3404	0.037745	0.096659	-0.45	MULTISPECIES: two-component system sensor histidine kinase EnvZ	Two-component system; Biofilm formation - Escherichia coli
CsakCS931_RS17760	cap	0.003963	0.022181	-0.89	cAMP-activated global transcriptional regulator CRP	Two-component system; Quorum sensing; Biofilm formation - Pseudomonas aeruginosa; Biofilm formation

						- Escherichia coli; Biofilm formation - Vibrio cholerae
CsakCS931_RS18715	SNSL254_A4722	0.021603	0.06594	-0.69	RNA chaperone Hfq	Quorum sensing; RNA degradation; Biofilm formation - Vibrio cholerae
CsakCS931_RS19565	Z4389	0.002273	0.015017	-1.04	MULTISPECIES: 3',5'-cyclic-AMP phosphodiesterase	Purine metabolism; Biofilm formation - Pseudomonas aeruginosa
CsakCS931_RS20140	b2808	0.053084	0.120708	-0.68	MULTISPECIES: glycine cleavage system transcriptional regulator GcvA	Biofilm formation - Escherichia coli
CsakCS931_RS20685	Arnit_2199	1.48E-14	1.08E-12	9.64	metallophosphoesterase	Purine metabolism; Biofilm formation - Pseudomonas aeruginosa
CsakCS931_RS20785	PA0085	0.009504	0.039281	1.30	type VI secretion system tube protein Hcp	Biofilm formation - Pseudomonas aeruginosa; Bacterial secretion system
CsakCS931_RS20830	PA0083	9.60E-05	0.00118	2.15	MULTISPECIES: type VI secretion system contractile sheath small subunit	Biofilm formation - Pseudomonas aeruginosa
CsakCS931_RS20835	PA0084	0.002816	0.017735	1.80	type VI secretion system contractile sheath large subunit	Biofilm formation - Pseudomonas aeruginosa

CsakCS931_RS20845	/	0.582237	0.68925	0.27	type VI secretion protein ImpG	Biofilm formation - Pseudomonas aeruginosa; Bacterial secretion system
CsakCS931_RS20855	/	0.528059	0.641905	0.24	type VI secretion system baseplate subunit TssG	Biofilm formation - Pseudomonas aeruginosa

Table S5. Genes related to antibiotic resistance and virulence in XY001-Ami group.

	Gene id	Gene	p-value	q-value	log_2Foldchange	Description	Pathway Description
Genes associated with antibiotic resistance	CsakCS931_RS05905	Z2170	0.030897	0.207308	0.90	MULTISPECIES: MDR efflux pump AcrAB transcriptional activator MarA	Cationic antimicrobial peptide (CAMP) resistance
	CsakCS931_RS19550	b3035	0.138913	0.472499	0.52	MULTISPECIES: outer membrane channel protein TolC	beta-Lactam resistance; Cationic antimicrobial peptide (CAMP) resistance; Two-component system; Bacterial secretion system; Plant-pathogen interaction; Pertussis
	CsakCS931_RS16085	SBO_3657	0.000795	0.013176	0.81	MULTISPECIES: phosphate ABC transporter ATP-binding protein PstB	ABC transporters
	CsakCS931_RS10895	b0462	0.005067	0.056533	0.84	MULTISPECIES: multidrug efflux RND transporter permease subunit	beta-Lactam resistance; Cationic antimicrobial

						peptide (CAMP) resistance
CsakCS931_RS05165	STY1368	0.036558	0.231943	0.77	MULTISPECIES: peptide ABC transporter permease SapB	Cationic antimicrobial peptide (CAMP) resistance; ABC transporters
CsakCS931_RS05170	STM1692	0.355027	0.6326	0.36	MULTISPECIES: peptide ABC transporter substrate-binding protein SapA	Cationic antimicrobial peptide (CAMP) resistance; ABC transporters
CsakCS931_RS08100	b1113	0.004044	0.04761	-1.09	MULTISPECIES: L,D-transpeptidase family protein	Cationic antimicrobial peptide (CAMP) resistance
CsakCS931_RS06735	Z2647	0.000123	0.002902	1.89	MULTISPECIES: glutathione transferase GstA	Glutathione metabolism; Metabolism of xenobiotics by cytochrome P450; Drug metabolism -

						cytochrome P450; Drug metabolism - other enzymes; Platinum drug resistance; Longevity regulating pathway - worm; Pathways in cancer; Chemical carcinogenesis; Hepatocellular carcinoma; Fluid shear stress and atherosclerosis
CsakCS931_RS09805	b0763	0.032861	0.216738	0.88	molybdate ABC transporter substrate-binding protein	ABC transporters
CsakCS931_RS18435	gstA	0.000715	0.012071	0.72	glutathione S-transferase	Glutathione metabolism; Metabolism of xenobiotics by cytochrome P450; Drug metabolism - cytochrome P450; Drug metabolism - other enzymes;

						Platinum drug resistance; Longevity regulating pathway - worm; Pathways in cancer; Chemical carcinogenesis; Hepatocellular carcinoma; Fluid shear stress and atherosclerosis
CsakCS931_RS16295	STM3997	0.810971	0.913343	0.08	MULTISPECIES: thiol:disulfide interchange protein DsbA	Cationic antimicrobial peptide (CAMP) resistance
CsakCS931_RS01400	b2435	0.383886	0.650691	-0.45	MULTISPECIES: N-acetylmuramoyl-L-alanine amidase AmiA	Cationic antimicrobial peptide (CAMP) resistance
CsakCS931_RS12245	Z0173	0.503735	0.728675	0.23	MULTISPECIES: serine endoprotease DegP	Cationic antimicrobial peptide (CAMP) resistance; Two-component system

CsakCS931_RS14055	W5S_4173	0.162153	0.502522	0.53	two-component system response regulator PmrA	Cationic antimicrobial peptide (CAMP) resistance; Two-component system
CsakCS931_RS12015	Ent638_0743	0.043034	0.253649	0.80	MULTISPECIES: endonuclease/exonuclease/phosphatase family protein	/
CsakCS931_RS17730	STM3472	0.254757	0.577685	-0.55	MULTISPECIES: peptidylprolyl isomerase A	Cationic antimicrobial peptide (CAMP) resistance; Necroptosis
CsakCS931_RS15870	b3673	0.095545	0.405529	0.65	multidrug resistance protein D	
CsakCS931_RS05485	T1E_0241	0.595859	0.79157	0.22	efflux transporter outer membrane subunit	beta-Lactam resistance; Quorum sensing
CsakCS931_RS02545	b2180	0.319764	0.613693	-0.34	microcin C ABC transporter ATP-binding protein YejF	ABC transporters
CsakCS931_RS12590	b0086	0.198085	0.541721	0.47	UDP-N-acetylmuramoyl-tripeptide--D-alanyl-D-alanine ligase	Lysine biosynthesis; Peptidoglycan biosynthesis; Vancomycin resistance

CsakCS931_RS12585	ESA_03251	0.261202	0.578087	0.34	MULTISPECIES: phospho-N-acetylmuramoyl-pentapeptide-transferase	Peptidoglycan biosynthesis; Vancomycin resistance
CsakCS931_RS12570	ESA_03248	0.771044	0.890071	-0.05	MULTISPECIES: undecaprenyldiphospho-muramoylpentapeptide beta-N-acetylglucosaminyltransferase	Peptidoglycan biosynthesis; Vancomycin resistance; Cell cycle - Caulobacter
CsakCS931_RS12560	b0092	0.124633	0.453531	0.46	D-alanine--D-alanine ligase	D-Alanine metabolism; Peptidoglycan biosynthesis; Vancomycin resistance
CsakCS931_RS04145	b1846	0.019133	0.151091	-1.07	tellurite resistance TerB family protein	/
CsakCS931_RS05510	PA1129	0.062383	0.323014	0.74	FosA family fosfomycin resistance glutathione transferase	/
CsakCS931_RS05615	b1430	0.071616	0.3473	-0.64	tellurite resistance methyltransferase TehB	/
CsakCS931_RS05900		0.002618	0.034236	1.79	MULTISPECIES: multiple antibiotic resistance protein MarB	/
CsakCS931_RS06505	b0543	0.121919	0.450345	0.83	Ethidium bromide-methyl viologen resistance protein EmrE	/

CsakCS931_RS12435	traT	0.157029	0.496094	-0.93	MULTISPECIES: complement resistance protein TraT	/
CsakCS931_RS13270	ACIAD3023	0.300676	0.599076	-0.48	MULTISPECIES: organic hydroperoxide resistance protein	/
CsakCS931_RS15350	b3910	0.566724	0.775367	-0.16	6-N-hydroxylaminopurine resistance protein	/
CsakCS931_RS16990	ACIAD3023	6.24E-07	3.29E-05	2.39	organic hydroperoxide resistance protein	/
CsakCS931_RS17805	BSU00260	0.004159	0.048621	-1.09	toxic anion resistance protein	/
CsakCS931_RS18790	b4189	8.20E-06	0.000298	1.72	biofilm peroxide resistance protein BsmA	/
CsakCS931_RS00895	ESA_00711	0.498462	0.725227	0.29	hypothetical protein ESA_00711	Glycine, serine and threonine metabolism; Cyanoamino acid metabolism; Glyoxylate and dicarboxylate metabolism; One carbon pool by folate; Methane metabolism; Antifolate resistance
CsakCS931_RS02150	b2301	0.812877	0.9139	-0.09	glutathione transferase	Glutathione metabolism;

						Metabolism of xenobiotics by cytochrome P450; Drug metabolism - cytochrome P450; Drug metabolism - other enzymes; Platinum drug resistance; Longevity regulating pathway - worm; Pathways in cancer; Chemical carcinogenesis; Hepatocellular carcinoma; Fluid shear stress and atherosclerosis
CsakCS931_RS02425	SL1344_2237	0.024098	0.174279	0.84	MULTISPECIES: porin OmpC	beta-Lactam resistance; Two-component system
CsakCS931_RS04545	Z1953	0.409514	0.666157	0.25	MULTISPECIES: catabolic alanine racemase DadX	D-Alanine metabolism; Vancomycin resistance

CsakCS931_RS04885	STM1746	0.004295	0.049735	0.72	MULTISPECIES: oligopeptide ABC transporter substrate-binding protein OppA	beta-Lactam resistance; ABC transporters; Quorum sensing
CsakCS931_RS04890	Z2020	0.490919	0.719917	0.17	MULTISPECIES: oligopeptide ABC transporter permease OppB	beta-Lactam resistance; ABC transporters; Quorum sensing
CsakCS931_RS04895	Z2021	0.731729	0.86864	0.11	MULTISPECIES: oligopeptide ABC transporter permease OppC	beta-Lactam resistance; ABC transporters; Quorum sensing
CsakCS931_RS04900	b1246	0.049048	0.275393	0.58	MULTISPECIES: ABC transporter ATP-binding protein	beta-Lactam resistance; ABC transporters; Quorum sensing
CsakCS931_RS04905	STM1742	0.483318	0.716457	-0.23	MULTISPECIES: ABC transporter ATP-binding protein	beta-Lactam resistance; ABC transporters; Quorum sensing
CsakCS931_RS05150	STM1696	0.130184	0.463553	0.57	MULTISPECIES: peptide ABC transporter ATP-binding protein SapF	Cationic antimicrobial peptide (CAMP) resistance; ABC transporters

CsakCS931_RS05155	STM1695	0.242996	0.569766	0.35	MULTISPECIES: peptide ABC transporter ATP-binding protein SapD	Cationic antimicrobial peptide (CAMP) resistance; ABC transporters
CsakCS931_RS05160	SF1297	0.77495	0.892705	-0.11	peptide ABC transporter permease SapC	Cationic antimicrobial peptide (CAMP) resistance; ABC transporters
CsakCS931_RS05315	b1329	0.338429	0.628248	0.44	peptide ABC transporter substrate-binding protein	beta-Lactam resistance; ABC transporters; Quorum sensing
CsakCS931_RS06210	ampC	0.468551	0.703977	-0.16	class C beta-lactamase CSA-1	beta-Lactam resistance; Two-component system
CsakCS931_RS06805	STM4293	0.022712	0.169259	0.90	phosphoethanolamine transferase EptA	Lipopolysaccharide biosynthesis; Cationic antimicrobial peptide (CAMP) resistance
CsakCS931_RS08030	SF1149	0.033107	0.217314	1.06	MULTISPECIES: two-component system response regulator PhoP	Cationic antimicrobial peptide (CAMP)

						resistance; Two-component system
CsakCS931_RS08035	STY1270	0.259047	0.578087	0.47	two-component system sensor histidine kinase PhoQ	Cationic antimicrobial peptide (CAMP) resistance; Two-component system
CsakCS931_RS08130	ESA_02237	0.018546	0.148421	0.91	beta-N-acetylhexosaminidase	Amino sugar and nucleotide sugar metabolism; Glycosaminoglycan degradation; beta-Lactam resistance
CsakCS931_RS08610		0.065817	0.329289	-1.10	HARLDQ motif MBL-fold protein	beta-Lactam resistance
CsakCS931_RS08640	b1243	0.059913	0.313432	0.52	peptide ABC transporter substrate-binding protein	beta-Lactam resistance; ABC transporters; Quorum sensing
CsakCS931_RS08810	ECP_0940	0.060381	0.314957	0.75	outer membrane protein F	beta-Lactam resistance; Two-component system
CsakCS931_RS09230	c0923	0.013145	0.113152	0.58	MULTISPECIES: glutathione S-transferase family protein	Glutathione metabolism; Metabolism of

						xenobiotics by cytochrome P450; Drug metabolism - cytochrome P450; Drug metabolism - other enzymes; Platinum drug resistance; Longevity regulating pathway - worm; Pathways in cancer; Chemical carcinogenesis; Hepatocellular carcinoma; Fluid shear stress and atherosclerosis
CsakCS931_RS10325	Z0781	0.024124	0.174279	-0.84	MULTISPECIES: peptidoglycan DD-transpeptidase MrdA	Peptidoglycan biosynthesis; beta-Lactam resistance
CsakCS931_RS10385	crcA	0.762676	0.887103	0.13	MULTISPECIES: phospholipid:lipid A palmitoyltransferase	Lipopolysaccharide biosynthesis; Cationic antimicrobial peptide (CAMP)

						resistance; Pertussis
CsakCS931_RS10760	STM0498	0.000139	0.003187	1.19	copper-exporting P-type ATPase CopA	Platinum drug resistance; MAPK signaling pathway - plant
CsakCS931_RS10890	Z0578	0.051584	0.284964	0.64	MULTISPECIES: multidrug efflux RND transporter periplasmic adaptor subunit AcrA	beta-Lactam resistance; Cationic antimicrobial peptide (CAMP) resistance
CsakCS931_RS11165	b0433	0.000552	0.009933	-1.16	muropeptide MFS transporter AmpG	beta-Lactam resistance
CsakCS931_RS11440	b0381	0.197926	0.541721	-0.38	D-alanine--Amp ligase	D-Alanine metabolism; Peptidoglycan biosynthesis; Vancomycin resistance
CsakCS931_RS12090	b0192	0.30188	0.599574	0.42	envelope stress response activation lipoprotein NlpE	Cationic antimicrobial peptide (CAMP) resistance
CsakCS931_RS12160	ESA_03160	0.045482	0.264258	0.83	MULTISPECIES: acyl-ACP--UDP-N-acetylglucosamine O-acyltransferase	Lipopolysaccharide biosynthesis;

CsakCS931_RS12600	b0084	0.660304	0.830459	0.13	MULTISPECIES: peptidoglycan glycosyltransferase FtsI	Cationic antimicrobial peptide (CAMP) resistance Peptidoglycan biosynthesis; beta-Lactam resistance
CsakCS931_RS12770	atsR	0.015281	0.128381	-0.77	Dihydrofolate reductase	One carbon pool by folate; Folate biosynthesis; Antifolate resistance
CsakCS931_RS14060	W5S_4174	0.342467	0.630402	0.31	two-component system sensor histidine kinase PmrB	Cationic antimicrobial peptide (CAMP) resistance; Two-component system
CsakCS931_RS14515	ESA_03670	0.740424	0.874794	-0.09	MULTISPECIES: bifunctional phosphoribosylaminoimidazolecarboxamide formyltransferase/IMP cyclohydrolase	Purine metabolism; One carbon pool by folate; Antifolate resistance
CsakCS931_RS15245	b3941	0.099149	0.412943	-0.80	methylenetetrahydrofolate reductase	One carbon pool by folate; Carbon fixation pathways in prokaryotes;

CsakCS931_RS16115	SF3809	0.288144	0.597055	0.37	glutamine--fructose-6-phosphate transaminase (isomerizing)	Antifolate resistance Alanine, aspartate and glutamate metabolism; Amino sugar and nucleotide sugar metabolism; Insulin resistance
CsakCS931_RS16680	b3911	0.740507	0.874794	0.15	MULTISPECIES: envelope stress sensor histidine kinase CpxA	Cationic antimicrobial peptide (CAMP) resistance; Two-component system
CsakCS931_RS16685	Z5457	0.720323	0.86063	-0.12	MULTISPECIES: envelope stress response regulator transcription factor CpxR	Cationic antimicrobial peptide (CAMP) resistance; Two-component system
CsakCS931_RS16995	STM3635	0.002686	0.034855	-0.63	kdo(2)-lipid A phosphoethanolamine 7"-transferase	Lipopolysaccharide biosynthesis; Cationic antimicrobial peptide (CAMP) resistance

CsakCS931_RS17470	SF3451	0.000807	0.013176	1.02	MULTISPECIES: glycogen phosphorylase	Starch and sucrose metabolism; Biofilm formation - Escherichia coli; Necroptosis; Insulin signaling pathway; Glucagon signaling pathway; Insulin resistance
CsakCS931_RS17510	b3417	0.570015	0.777598	0.14	maltodextrin phosphorylase	Starch and sucrose metabolism; Biofilm formation - Escherichia coli; Necroptosis; Insulin signaling pathway; Glucagon signaling pathway; Insulin resistance
CsakCS931_RS17615	b3396	0.221935	0.558938	-0.48	MULTISPECIES: peptidoglycan glycosyltransferase/peptidoglycan DD-transpeptidase MrcA	Peptidoglycan biosynthesis; beta-Lactam resistance
CsakCS931_RS18360	STY4443	0.598842	0.793413	0.30	alanine racemase	D-Alanine metabolism;

	CsakCS931_RS18700	b4169	0.796483	0.904934	0.07	N-acetylmuramoyl-L-alanine amidase AmiB	Vancomycin resistance Cationic antimicrobial peptide (CAMP) resistance
	CsakCS931_RS20055	ESA_00482	0.3569	0.6326	0.30	thymidylate synthase	Pyrimidine metabolism; One carbon pool by folate; Antifolate resistance
	CsakCS931_RS20105	c3411	0.002215	0.030353	-0.72	N-acetylmuramoyl-L-alanine amidase	Cationic antimicrobial peptide (CAMP) resistance
	CsakCS931_RS20610	ML0773	0.09434	0.402856	-0.38	MULTISPECIES: response regulator transcription factor	beta-Lactam resistance
Genes associated with bacterial virulence	CsakCS931_RS06360	/	0.230963	0.569972	0.35	virulence factor SrfB	/
	CsakCS931_RS06365	/	0.57065	0.7825	-0.42	virulence factor	/
	CsakCS931_RS07610	b0877	0.076818	0.369419	0.93	Virulence factor VirK	/
	CsakCS931_RS15765	PA0086	0.314644	0.613434	-0.51	protein of avirulence locus ImpE	/
	CsakCS931_RS16410	ESA_04062	0.561279	0.775036	-0.19	virulence factor BrkB family protein	/

Genes associated with Biofilms	CsakCS931_RS08375	c1327	7.29E-05	0.00193	-2.10	MULTISPECIES: biofilm formation regulator BssS	
	CsakCS931_RS00150	STM2924	0.000653	0.011795	-1.47	MULTISPECIES: RNA polymerase sigma factor RpoS	Biofilm formation - Escherichia coli; Biofilm formation - Vibrio cholerae
	CsakCS931_RS00250	ECDH10B_2864	0.004452	0.05368	-1.36	MULTISPECIES: carbon storage regulator CsrA	Two-component system; Biofilm formation - Pseudomonas aeruginosa; Biofilm formation - Escherichia coli; Biofilm formation - Vibrio cholerae
	CsakCS931_RS00300	ESA_00581	1.33E-06	6.83E-05	-1.84	MULTISPECIES: S-ribosylhomocysteine lyase	Cysteine and methionine metabolism; Quorum sensing; Biofilm formation - Escherichia coli; Biofilm formation - Vibrio cholerae
	CsakCS931_RS01175	b2479	0.092593	0.4077	-0.46	MULTISPECIES: glycine cleavage system transcriptional repressor	Biofilm formation - Escherichia coli

CsakCS931_RS01455	STM2433	0.00163	0.024438	-1.45	MULTISPECIES: PTS glucose transporter subunit IIA	Glycolysis / Gluconeogenesis; Starch and sucrose metabolism; Amino sugar and nucleotide sugar metabolism; Biofilm formation - Escherichia coli; Phosphotransferas e system (PTS); Biofilm formation - Vibrio cholerae
CsakCS931_RS02410	STY2496	0.073243	0.359672	0.39	Sensor kinase protein RcsC	Two-component system; Biofilm formation - Escherichia coli
CsakCS931_RS02415	SF2301	0.4491	0.697842	-0.28	MULTISPECIES: transcriptional regulator RcsB	Two-component system; Biofilm formation - Escherichia coli
CsakCS931_RS02420	b2216	0.468021	0.708919	-0.27	Sensor-like histidine kinase RcsD	Two-component system; Biofilm formation - Escherichia coli

CsakCS931_RS02805	b2127	0.066653	0.343412	-0.41	MULTISPECIES: MerR family transcriptional regulator	Biofilm formation - Escherichia coli
CsakCS931_RS02975	b2067	0.753199	0.885953	-0.07	diguanylate cyclase	Biofilm formation - Escherichia coli
CsakCS931_RS03000	STM2118	0.388976	0.656164	0.36	MULTISPECIES: polysaccharide export protein	Two-component system; Biofilm formation - Escherichia coli
CsakCS931_RS03075	b2047	0.388132	0.655552	-0.61	MULTISPECIES: undecaprenyl-phosphate glucose phosphotransferase	Biofilm formation - Vibrio cholerae
CsakCS931_RS03470	SSON_2013	0.622172	0.811777	0.12	cellulose biosynthesis regulator YedQ	Biofilm formation - Escherichia coli
CsakCS931_RS03490	rcsA	0.334051	0.627357	0.30	MULTISPECIES: transcriptional regulator RcsA	Two-component system; Quorum sensing; Biofilm formation - Escherichia coli
CsakCS931_RS03700	STM1956	0.373306	0.647993	0.22	MULTISPECIES: RNA polymerase sigma factor FliA	Two-component system; Biofilm formation - Pseudomonas aeruginosa; Biofilm formation - Escherichia coli; Flagellar assembly; Biofilm

						formation - Vibrio cholerae
CsakCS931_RS03705	STY2163	0.32919	0.623959	0.45	MULTISPECIES: flagella biosynthesis regulatory protein FliZ	Biofilm formation - Escherichia coli
CsakCS931_RS03730	b1916	0.938333	0.977217	-0.04	MULTISPECIES: transcriptional regulator SdiA	Two-component system; Quorum sensing; Biofilm formation - Escherichia coli
CsakCS931_RS03740	SF1957	0.480294	0.718964	-0.36	MULTISPECIES: UvrY/SirA/GacA family response regulator transcription factor	Two-component system; Biofilm formation - Pseudomonas aeruginosa; Biofilm formation - Escherichia coli; Biofilm formation - Vibrio cholerae
CsakCS931_RS03870	ESA_01337	0.084537	0.388976	-0.70	Flagellar transcriptional activator FlhD	Two-component system; Quorum sensing; Biofilm formation - Escherichia coli; Flagellar assembly
CsakCS931_RS03875	STM1924	0.192828	0.541952	-0.55	MULTISPECIES: flagellar transcriptional regulator FlhC	Two-component system; Quorum

						sensing; Biofilm formation - Escherichia coli; Flagellar assembly
CsakCS931_RS04310	b1815	0.709244	0.858489	-0.10	EAL domain-containing protein	Biofilm formation - Escherichia coli
CsakCS931_RS04570	STM1798	0.254128	0.579795	-0.28	MULTISPECIES: flagellar brake protein YcgR	Biofilm formation - Escherichia coli
CsakCS931_RS05000	STM1723	0.703974	0.855819	0.15	anthranilate synthase subunit I	Phenylalanine, tyrosine and tryptophan biosynthesis; Phenazine biosynthesis; Quorum sensing; Biofilm formation - Pseudomonas aeruginosa
CsakCS931_RS05130	b1285	8.73E-05	0.002288	-1.12	cyclic di-GMP phosphodiesterase	Quorum sensing; Biofilm formation - Escherichia coli
CsakCS931_RS05965	c0492	0.046211	0.274734	0.80	sensor domain-containing diguanylate cyclase	Biofilm formation - Escherichia coli
CsakCS931_RS06940	PA0083	0.046513	0.275519	1.32	MULTISPECIES: type VI secretion system contractile sheath small subunit	Biofilm formation - Pseudomonas aeruginosa

CsakCS931_RS08160	b1101	0.038855	0.248593	-0.89	MULTISPECIES: PTS glucose transporter subunit IIBC	Glycolysis / Gluconeogenesis; Amino sugar and nucleotide sugar metabolism; Phosphotransferase system (PTS); Biofilm formation - Vibrio cholerae
CsakCS931_RS08320	STM1172	0.70063	0.853912	0.16	MULTISPECIES: anti-sigma-28 factor FlgM	Two-component system; Biofilm formation - Pseudomonas aeruginosa; Biofilm formation - Escherichia coli; Flagellar assembly
CsakCS931_RS11590	VC_0137	0.408642	0.669759	-0.21	EAL domain-containing protein	Biofilm formation - Vibrio cholerae
CsakCS931_RS12325	SF0137	0.018723	0.153384	-1.00	DnaK suppressor protein	Biofilm formation - Escherichia coli
CsakCS931_RS13000	Z6004	0.140584	0.480392	-0.59	MULTISPECIES: two-component system response regulator ArcA	Two-component system; Biofilm formation - Escherichia coli

CsakCS931_RS14160	ntrA	0.209847	0.556398	-0.45	MULTISPECIES: RNA polymerase factor sigma-54	Two-component system; Biofilm formation - Vibrio cholerae
CsakCS931_RS14195	SF3250	0.359617	0.638542	-0.20	aerobic respiration two-component sensor histidine kinase ArcB	Two-component system; Biofilm formation - Escherichia coli
CsakCS931_RS14435	b3261	0.247601	0.576632	0.61	MULTISPECIES: DNA-binding transcriptional regulator Fis	Biofilm formation - Vibrio cholerae
CsakCS931_RS14925	STY3620	0.104529	0.428164	-0.67	class I adenylate cyclase	Purine metabolism; Biofilm formation - Escherichia coli; Biofilm formation - Vibrio cholerae
CsakCS931_RS15010	b3787	0.841584	0.932014	0.06	MULTISPECIES: UDP-N-acetyl-D-mannosamine dehydrogenase	Amino sugar and nucleotide sugar metabolism; Two-component system; Biofilm formation - Vibrio cholerae
CsakCS931_RS15015	b3786	0.886571	0.949938	-0.06	MULTISPECIES: UDP-N-acetylglucosamine 2-epimerase (non-hydrolyzing)	Amino sugar and nucleotide sugar metabolism; Two-

						component system; Biofilm formation - Vibrio cholerae
CsakCS931_RS15180	b3961	0.262377	0.580262	-0.51	MULTISPECIES: DNA-binding transcriptional regulator OxyR	Biofilm formation - Escherichia coli
CsakCS931_RS15740	PA0090	0.338188	0.631416	0.45	type VI secretion system ATPase TssH	Biofilm formation - Pseudomonas aeruginosa; Bacterial secretion system
CsakCS931_RS15750		0.322538	0.620136	0.43	MULTISPECIES: type VI secretion system baseplate subunit TssG	Biofilm formation - Pseudomonas aeruginosa
CsakCS931_RS15795	PA0085	0.000172	0.004057	-2.31	MULTISPECIES: type VI secretion system tube protein Hcp	Biofilm formation - Pseudomonas aeruginosa; Bacterial secretion system
CsakCS931_RS15815	PA0084	0.002538	0.035259	-1.15	MULTISPECIES: type VI secretion system contractile sheath large subunit	Biofilm formation - Pseudomonas aeruginosa
CsakCS931_RS15825	PA0082	0.000184	0.004251	-1.37	type VI secretion system protein TssA	Biofilm formation - Pseudomonas aeruginosa

CsakCS931_RS15830		0.000318	0.006584	-1.23	MULTISPECIES: type VI secretion system-associated protein TagF	Biofilm formation - Pseudomonas aeruginosa
CsakCS931_RS15835	PA0077	0.010986	0.104035	-0.82	MULTISPECIES: type VI secretion system membrane subunit TssM	Biofilm formation - Pseudomonas aeruginosa; Bacterial secretion system
CsakCS931_RS15845	PA0079	0.488494	0.723529	0.25	MULTISPECIES: type VI secretion system baseplate subunit TssK	Biofilm formation - Pseudomonas aeruginosa
CsakCS931_RS16670	STM3699	0.954112	0.983351	-0.02	serine acetyltransferase	Cysteine and methionine metabolism; Sulfur metabolism; Biofilm formation - Vibrio cholerae
CsakCS931_RS16805	PA5267	0.237804	0.574403	1.50	type VI secretion system tube protein Hcp	Biofilm formation - Pseudomonas aeruginosa; Bacterial secretion system
CsakCS931_RS17070	b3533	0.148166	0.492154	0.68	UDP-forming cellulose synthase catalytic subunit	Starch and sucrose metabolism;

						Biofilm formation - Escherichia coli
CsakCS931_RS17110	b3525	0.023866	0.180549	1.24	cyclic-guanylate-specific phosphodiesterase	Biofilm formation - Escherichia coli
CsakCS931_RS17460	ESA_04311	0.001114	0.017848	-1.07	MULTISPECIES: glucose-1-phosphate adenylyltransferase	Starch and sucrose metabolism; Amino sugar and nucleotide sugar metabolism; Biofilm formation - Escherichia coli
CsakCS931_RS17465	ESA_04312	0.002472	0.034529	-1.10	MULTISPECIES: glycogen synthase GlgA	Starch and sucrose metabolism; Biofilm formation - Escherichia coli
CsakCS931_RS17470	SF3451	0.000971	0.016058	-1.02	MULTISPECIES: glycogen phosphorylase	Starch and sucrose metabolism; Biofilm formation - Escherichia coli; Necroptosis; Insulin signaling pathway; Glucagon signaling pathway; Insulin resistance

CsakCS931_RS17510	b3417	0.571297	0.782786	-0.14	maltodextrin phosphorylase	Starch and sucrose metabolism; Biofilm formation - Escherichia coli; Necroptosis; Insulin signaling pathway; Glucagon signaling pathway; Insulin resistance
CsakCS931_RS17570	Z4760	4.27E-05	0.001233	-1.08	hypothetical protein ESA_04334	Two-component system; Biofilm formation - Escherichia coli
CsakCS931_RS17575	b3404	0.85264	0.936146	0.04	MULTISPECIES: two-component system sensor histidine kinase EnvZ	Two-component system; Biofilm formation - Escherichia coli
CsakCS931_RS17760	cap	0.085773	0.392546	-0.78	cAMP-activated global transcriptional regulator CRP	Two-component system; Quorum sensing; Biofilm formation - Pseudomonas aeruginosa; Biofilm formation - Escherichia coli;

CsakCS931_RS18715	SNSL254_A 4722	0.082233	0.382855	-0.66	RNA chaperone Hfq	Biofilm formation - Vibrio cholerae Quorum sensing; RNA degradation; Biofilm formation - Vibrio cholerae
CsakCS931_RS19565	Z4389	0.206238	0.553142	-0.58	MULTISPECIES: 3',5'-cyclic-AMP phosphodiesterase	Purine metabolism; Biofilm formation - Pseudomonas aeruginosa
CsakCS931_RS20140	b2808	0.123603	0.460619	-0.72	MULTISPECIES: glycine cleavage system transcriptional regulator GcvA	Biofilm formation - Escherichia coli
CsakCS931_RS20685	Arnit_2199	0.6776	0.842579	-0.29	metallophosphoesterase	Purine metabolism; Biofilm formation - Pseudomonas aeruginosa
CsakCS931_RS20785	PA0085	0.145547	0.487954	0.91	type VI secretion system tube protein Hcp	Biofilm formation - Pseudomonas aeruginosa; Bacterial secretion system
CsakCS931_RS20830	PA0083	0.060264	0.322585	1.20	MULTISPECIES: type VI secretion system contractile sheath small subunit	Biofilm formation - Pseudomonas aeruginosa

CsakCS931_RS20835	PA0084	0.347543	0.632514	0.82	type VI secretion system contractile sheath large subunit	Biofilm formation - Pseudomonas aeruginosa
CsakCS931_RS20845	/	0.416729	0.674715	0.58	type VI secretion protein ImpG	Biofilm formation - Pseudomonas aeruginosa; Bacterial secretion system
CsakCS931_RS20855	/	0.278498	0.593831	0.50	type VI secretion system baseplate subunit TssG	Biofilm formation - Pseudomonas aeruginosa

Table S6. Genes related to antibiotic resistance and virulence in XY001-Tet group.

	Gene id	Gene	p-Value	q-Value	log_2Foldchange	Description	Pathway Description
Genes associated with antibiotic resistance	CsakCS931_RS02170	ramA	5.27E-43	1.29E-39	4.79	MULTISPECIES: RamA family antibiotic efflux transcriptional regulator	/
	CsakCS931_RS05935	b1526	3.41E-05	0.000362	1.33	LysR family transcriptional regulator	/
	CsakCS931_RS02425	SL1344_2237	0.045349	0.134537	0.68	MULTISPECIES: porin OmpC	beta-Lactam resistance; Two-component system
	CsakCS931_RS19550	b3035	0.001748	0.009708	0.95	MULTISPECIES: outer membrane channel protein TolC	beta-Lactam resistance; Cationic antimicrobial peptide (CAMP) resistance; Two-component system; Bacterial secretion system; Plant-pathogen interaction; Pertussis
	CsakCS931_RS00160	b1828	0.355437	0.516291	-0.44	MFS transporter	/
	CsakCS931_RS00310	b2686	0.293623	0.455632	-0.48	MULTISPECIES: multidrug efflux MFS transporter permease subunit EmrB	/

CsakCS931_RS01355	SCH_0624	0.119599	0.260726	-0.79	MFS transporter	/
CsakCS931_RS02745	b2137	0.963897	0.9749	-0.01	MULTISPECIES: SDR family oxidoreductase	/
CsakCS931_RS03425	BSU33190	0.125007	0.267503	-0.74	SDR family oxidoreductase	/
CsakCS931_RS04340	b0868	8.05E-06	0.000108	-1.59	SDR family NAD(P)-dependent oxidoreductase	/
CsakCS931_RS06140	SSP1627	0.760287	0.844611	-0.14	SDR family oxidoreductase	/
CsakCS931_RS00165	PA2258	0.254402	0.417022	-0.43	LysR family transcriptional regulator	/
CsakCS931_RS13100	PA2258	0.314021	0.475155	-0.47	LysR family transcriptional regulator	/
CsakCS931_RS16770	Z0352	0.187061	0.345614	-0.57	aldehyde dehydrogenase iron-sulfur subunit	Purine metabolism
CsakCS931_RS05485	T1E_0241	0.203244	0.36295	0.41	efflux transporter outer membrane subunit	beta-Lactam resistance; Quorum sensing
CsakCS931_RS11165	b0433	0.06318	0.171348	-0.57	muropeptide MFS transporter AmpG	beta-Lactam resistance
CsakCS931_RS04145	b1846	0.000583	0.003856	1.22	tellurite resistance TerB family protein	/
CsakCS931_RS18790	b4189	1.77E-09	9.14E-08	1.81	biofilm peroxide resistance protein BsmA	/
CsakCS931_RS00895	ESA_00711	0.474867	0.625804	0.26	hypothetical protein ESA_00711	Glycine, serine and threonine metabolism;

						Cyanoamino acid metabolism; Glyoxylate and dicarboxylate metabolism; One carbon pool by folate; Methane metabolism; Antifolate resistance
CsakCS931_RS01400	b2435	0.902799	0.936785	0.05	MULTISPECIES: N-acetylmuramoyl-L-alanine amidase AmiA	Cationic antimicrobial peptide (CAMP) resistance
CsakCS931_RS02150	b2301	0.88457	0.924786	0.05	glutathione transferase	Glutathione metabolism; Metabolism of xenobiotics by cytochrome P450; Drug metabolism - cytochrome P450; Drug metabolism - other enzymes; Platinum drug resistance; Longevity

						regulating pathway - worm; Pathways in cancer; Chemical carcinogenesis; Hepatocellular carcinoma; Fluid shear stress and atherosclerosis
CsakCS931_RS04545	Z1953	0.274912	0.435766	0.33	MULTISPECIES: catabolic alanine racemase DadX	D-Alanine metabolism; Vancomycin resistance
CsakCS931_RS04885	STM1746	0.047184	0.138386	0.50	MULTISPECIES: oligopeptide ABC transporter substrate-binding protein OppA	beta-Lactam resistance; ABC transporters; Quorum sensing
CsakCS931_RS04890	Z2020	0.617345	0.741844	-0.15	MULTISPECIES: oligopeptide ABC transporter permease OppB	beta-Lactam resistance; ABC transporters; Quorum sensing
CsakCS931_RS04900	b1246	0.133286	0.279827	0.41	MULTISPECIES: ABC transporter ATP-binding protein	beta-Lactam resistance; ABC transporters; Quorum sensing

CsakCS931_RS04905	STM1742	0.567785	0.704588	-0.22	MULTISPECIES: ABC transporter ATP-binding protein	beta-Lactam resistance; ABC transporters; Quorum sensing
CsakCS931_RS05165	STY1368	0.005236	0.024058	0.92	MULTISPECIES: peptide ABC transporter permease SapB	Cationic antimicrobial peptide (CAMP) resistance; ABC transporters
CsakCS931_RS05170	STM1692	0.019917	0.071153	0.79	MULTISPECIES: peptide ABC transporter substrate-binding protein SapA	Cationic antimicrobial peptide (CAMP) resistance; ABC transporters
CsakCS931_RS05315	b1329	0.627145	0.748661	0.21	peptide ABC transporter substrate-binding protein	beta-Lactam resistance; ABC transporters; Quorum sensing
CsakCS931_RS05905	Z2170	0.403475	0.561108	0.27	MULTISPECIES: MDR efflux pump AcrAB transcriptional activator MarA	Cationic antimicrobial peptide (CAMP) resistance
CsakCS931_RS06210	ampC	0.39709	0.555223	-0.21	class C beta-lactamase CSA-1	beta-Lactam resistance; Two-component system

CsakCS931_RS06735	Z2647	0.037592	0.115948	0.81	MULTISPECIES: glutathione transferase GstA	Glutathione metabolism; Metabolism of xenobiotics by cytochrome P450; Drug metabolism - cytochrome P450; Drug metabolism - other enzymes; Platinum drug resistance; Longevity regulating pathway - worm; Pathways in cancer; Chemical carcinogenesis; Hepatocellular carcinoma; Fluid shear stress and atherosclerosis
CsakCS931_RS06805	STM4293	0.005636	0.025467	0.94	phosphoethanolamine transferase EptA	Lipopolysaccharid e biosynthesis; Cationic antimicrobial

						peptide (CAMP) resistance
CsakCS931_RS08030	SF1149	0.000372	0.002666	1.49	MULTISPECIES: two-component system response regulator PhoP	Cationic antimicrobial peptide (CAMP) resistance; Two-component system
CsakCS931_RS08100	b1113	0.919413	0.94786	0.03	MULTISPECIES: L,D-transpeptidase family protein	Cationic antimicrobial peptide (CAMP) resistance
CsakCS931_RS08130	ESA_02237	0.000621	0.004057	0.97	beta-N-acetylhexosaminidase	Amino sugar and nucleotide sugar metabolism; Glycosaminoglycan degradation; beta-Lactam resistance
CsakCS931_RS08610		0.217053	0.378606	-0.49	HARLDQ motif MBL-fold protein	beta-Lactam resistance
CsakCS931_RS08640	b1243	0.469583	0.621693	0.19	peptide ABC transporter substrate-binding protein	beta-Lactam resistance; ABC transporters; Quorum sensing

CsakCS931_RS08810	ECP_0940	0.019727	0.070579	0.81	outer membrane protein F	beta-Lactam resistance; Two-component system
CsakCS931_RS09230	c0923	0.859535	0.909294	0.04	MULTISPECIES: glutathione S-transferase family protein	Glutathione metabolism; Metabolism of xenobiotics by cytochrome P450; Drug metabolism - cytochrome P450; Drug metabolism - other enzymes; Platinum drug resistance; Longevity regulating pathway - worm; Pathways in cancer; Chemical carcinogenesis; Hepatocellular carcinoma; Fluid shear stress and atherosclerosis

CsakCS931_RS10325	Z0781	0.714539	0.811833	-0.12	MULTISPECIES: peptidoglycan DD-transpeptidase MrdA	Peptidoglycan biosynthesis; beta-Lactam resistance
CsakCS931_RS10385	crcA	0.387956	0.548112	0.31	MULTISPECIES: phospholipid:lipid A palmitoyltransferase	Lipopolysaccharide biosynthesis; Cationic antimicrobial peptide (CAMP) resistance; Pertussis
CsakCS931_RS10760	STM0498	0.03342	0.105948	0.76	copper-exporting P-type ATPase CopA	Platinum drug resistance; MAPK signaling pathway - plant
CsakCS931_RS10890	Z0578	0.001744	0.009696	0.95	MULTISPECIES: multidrug efflux RND transporter periplasmic adaptor subunit AcrA	beta-Lactam resistance; Cationic antimicrobial peptide (CAMP) resistance
CsakCS931_RS10895	b0462	8.97E-06	0.000118	1.05	MULTISPECIES: multidrug efflux RND transporter permease subunit	beta-Lactam resistance; Cationic antimicrobial peptide (CAMP) resistance

CsakCS931_RS11440	b0381	0.367421	0.527905	-0.26	D-alanine--D-alanine ligase	D-Alanine metabolism; Peptidoglycan biosynthesis; Vancomycin resistance
CsakCS931_RS12090	b0192	0.228132	0.39015	0.34	envelope stress response activation lipoprotein NlpE	Cationic antimicrobial peptide (CAMP) resistance
CsakCS931_RS12160	ESA_03160	0.010289	0.041138	0.83	MULTISPECIES: acyl-ACP--UDP-N-acetylglucosamine O-acyltransferase	Lipopolysaccharide biosynthesis; Cationic antimicrobial peptide (CAMP) resistance
CsakCS931_RS12245	Z0173	0.382759	0.542935	0.26	MULTISPECIES: serine endoprotease DegP	Cationic antimicrobial peptide (CAMP) resistance; Two-component system
CsakCS931_RS12590	b0086	0.266542	0.428126	0.35	UDP-N-acetylmuramoyl-tripeptide--D-alanyl-D-alanine ligase	Lysine biosynthesis; Peptidoglycan biosynthesis;

CsakCS931_RS12600	b0084	0.062264	0.169895	0.52	MULTISPECIES: peptidoglycan glycosyltransferase FtsI	Vancomycin resistance Peptidoglycan biosynthesis; beta-Lactam resistance
CsakCS931_RS12770	atsR	0.330174	0.491299	-0.31	Dihydrofolate reductase	One carbon pool by folate; Folate biosynthesis; Antifolate resistance
CsakCS931_RS14055	W5S_4173	0.261637	0.423293	0.31	two-component system response regulator PmrA	Cationic antimicrobial peptide (CAMP) resistance; Two-component system
CsakCS931_RS14060	W5S_4174	0.192403	0.351245	0.39	two-component system sensor histidine kinase PmrB	Cationic antimicrobial peptide (CAMP) resistance; Two-component system
CsakCS931_RS14515	ESA_03670	0.082653	0.205395	0.51	MULTISPECIES: bifunctional phosphoribosylaminoimidazolecarboxamide formyltransferase/IMP cyclohydrolase	Purine metabolism; One carbon pool by folate; Antifolate resistance

CsakCS931_RS15245	b3941	0.025556	0.086505	0.71	methylenetetrahydrofolate reductase	One carbon pool by folate; Carbon fixation pathways in prokaryotes; Antifolate resistance
CsakCS931_RS16115	SF3809	0.233476	0.396109	0.31	glutamine--fructose-6-phosphate transaminase (isomerizing)	Alanine, aspartate and glutamate metabolism; Amino sugar and nucleotide sugar metabolism; Insulin resistance
CsakCS931_RS16295	STM3997	0.744604	0.832285	0.10	MULTISPECIES: thiol:disulfide interchange protein DsbA	Cationic antimicrobial peptide (CAMP) resistance
CsakCS931_RS16680	b3911	0.2017	0.36148	0.45	MULTISPECIES: envelope stress sensor histidine kinase CpxA	Cationic antimicrobial peptide (CAMP) resistance; Two-component system
CsakCS931_RS16685	Z5457	0.148134	0.299323	0.48	MULTISPECIES: envelope stress response regulator transcription factor CpxR	Cationic antimicrobial peptide (CAMP)

						resistance; Two-component system
CsakCS931_RS16995	STM3635	0.195742	0.355484	0.27	kdo(2)-lipid A phosphoethanolamine 7''-transferase	Lipopolysaccharide biosynthesis; Cationic antimicrobial peptide (CAMP) resistance
CsakCS931_RS17470	SF3451	0.000782	0.004908	0.96	MULTISPECIES: glycogen phosphorylase	Starch and sucrose metabolism; Biofilm formation - Escherichia coli; Necroptosis; Insulin signaling pathway; Glucagon signaling pathway; Insulin resistance
CsakCS931_RS17615	b3396	0.968321	0.977337	-0.01	MULTISPECIES: peptidoglycan glycosyltransferase/peptidoglycan DD-transpeptidase MrcA	Peptidoglycan biosynthesis; beta-Lactam resistance
CsakCS931_RS17730	STM3472	0.493173	0.642436	-0.26	MULTISPECIES: peptidylprolyl isomerase A	Cationic antimicrobial peptide (CAMP) resistance; Necroptosis

CsakCS931_RS18360	STY4443	0.304226	0.465359	0.48	alanine racemase	D-Alanine metabolism; Vancomycin resistance
CsakCS931_RS18435	gstA	0.000891	0.00548	0.75	glutathione S-transferase	Glutathione metabolism; Metabolism of xenobiotics by cytochrome P450; Drug metabolism - cytochrome P450; Drug metabolism - other enzymes; Platinum drug resistance; Longevity regulating pathway - worm; Pathways in cancer; Chemical carcinogenesis; Hepatocellular carcinoma; Fluid shear stress and atherosclerosis

	CsakCS931_RS18700	b4169	0.062327	0.169895	-0.52	N-acetylmuramoyl-L-alanine amidase AmiB	Cationic antimicrobial peptide (CAMP) resistance
	CsakCS931_RS20055	ESA_00482	0.00754	0.031756	0.80	thymidylate synthase	Pyrimidine metabolism; One carbon pool by folate; Antifolate resistance
	CsakCS931_RS20105	c3411	0.127335	0.27105	-0.38	N-acetylmuramoyl-L-alanine amidase	Cationic antimicrobial peptide (CAMP) resistance
	CsakCS931_RS20610	ML0773	0.297102	0.458333	0.24	MULTISPECIES: response regulator transcription factor	beta-Lactam resistance
Genes associated with bacterial virulence	CsakCS931_RS06360	/	0.466551	0.622109	0.17	virulence factor SrfB	/
	CsakCS931_RS06365	/	1.39E-08	5.31E-07	-2.19	virulence factor	/
	CsakCS931_RS07610	b0877	0.280712	0.445565	0.42	Virulence factor VirK	/
	CsakCS931_RS15765	PA0086	3.58E-07	8.35E-06	-1.81	protein of avirulence locus ImpE	/
	CsakCS931_RS16410	ESA_04062	0.573927	0.710769	-0.20	virulence factor BrkB family protein	/
Genes associ	CsakCS931_RS00150	STM2924	0.000654	0.004398	-1.11	MULTISPECIES: RNA polymerase sigma factor RpoS	Biofilm formation - Escherichia coli;

ated with Biofilms							Biofilm formation - Vibrio cholerae
	CsakCS931_RS00250	ECDH10B_2864	0.04058	0.125319	-0.72	MULTISPECIES: carbon storage regulator CsrA [Bacteria]	Two-component system; Biofilm formation - Pseudomonas aeruginosa; Biofilm formation - Escherichia coli; Biofilm formation - Vibrio cholerae
	CsakCS931_RS00300	ESA_00581	0.013373	0.052982	-0.73	MULTISPECIES: S-ribosylhomocysteine lyase	Cysteine and methionine metabolism; Quorum sensing; Biofilm formation - Escherichia coli; Biofilm formation - Vibrio cholerae
	CsakCS931_RS01175	b2479	0.666137	0.778734	-0.11	MULTISPECIES: glycine cleavage system transcriptional repressor	Biofilm formation - Escherichia coli
	CsakCS931_RS01455	STM2433	0.012095	0.048753	-0.87	MULTISPECIES: PTS glucose transporter subunit IIA	Glycolysis / Gluconeogenesis; Starch and sucrose metabolism; Amino sugar and

						nucleotide sugar metabolism; Biofilm formation - Escherichia coli; Phosphotransferase system (PTS); Biofilm formation - Vibrio cholerae
CsakCS931_RS02410	STY2496	0.713138	0.81291	-0.08	Sensor kinase protein RcsC	Two-component system; Biofilm formation - Escherichia coli
CsakCS931_RS02415	SF2301	0.134901	0.284972	-0.52	MULTISPECIES: transcriptional regulator RcsB	Two-component system; Biofilm formation - Escherichia coli
CsakCS931_RS02420	b2216	0.026115	0.089941	-0.74	Sensor-like histidine kinase RcsD	Two-component system; Biofilm formation - Escherichia coli
CsakCS931_RS02805	b2127	0.45983	0.616363	-0.20	MULTISPECIES: MerR family transcriptional regulator	Biofilm formation - Escherichia coli
CsakCS931_RS02975	b2067	0.22998	0.395234	-0.31	diguanylate cyclase	Biofilm formation - Escherichia coli
CsakCS931_RS03470	SSON_2013	0.006368	0.028829	-0.74	cellulose biosynthesis regulator YedQ	Biofilm formation - Escherichia coli

CsakCS931_RS03730	b1916	0.192847	0.354703	-0.55	MULTISPECIES: transcriptional regulator SdiA	Two-component system; Quorum sensing; Biofilm formation - Escherichia coli
CsakCS931_RS03740	SF1957	0.052583	0.151836	-0.82	MULTISPECIES: UvrY/SirA/GacA family response regulator transcription factor	Two-component system; Biofilm formation - Pseudomonas aeruginosa; Biofilm formation - Escherichia coli; Biofilm formation - Vibrio cholerae
CsakCS931_RS03870	ESA_01337	0.005841	0.027019	-1.04	Flagellar transcriptional activator FlhD [Cronobacter sakazakii 696]	Two-component system; Quorum sensing; Biofilm formation - Escherichia coli; Flagellar assembly
CsakCS931_RS03875	STM1924	0.007587	0.032916	-0.98	MULTISPECIES: flagellar transcriptional regulator FlhC	Two-component system; Quorum sensing; Biofilm formation - Escherichia coli; Flagellar assembly

CsakCS931_RS04310	b1815	0.584869	0.720131	-0.15	EAL domain-containing protein	Biofilm formation - Escherichia coli
CsakCS931_RS04570	STM1798	0.024701	0.08659	-0.57	MULTISPECIES: flagellar brake protein YcgR	Biofilm formation - Escherichia coli
CsakCS931_RS05000	STM1723	0.715239	0.813257	-0.13	anthranilate synthase subunit I	Phenylalanine, tyrosine and tryptophan biosynthesis; Phenazine biosynthesis; Quorum sensing; Biofilm formation - Pseudomonas aeruginosa
CsakCS931_RS05130	b1285	0.000791	0.005146	-0.90	cyclic di-GMP phosphodiesterase	Quorum sensing; Biofilm formation - Escherichia coli
CsakCS931_RS08160	b1101	0.355945	0.519782	-0.33	MULTISPECIES: PTS glucose transporter subunit IIBC	Glycolysis / Gluconeogenesis; Amino sugar and nucleotide sugar metabolism; Phosphotransferase system (PTS); Biofilm formation - Vibrio cholerae

CsakCS931_RS11590	VC_0137	0.439135	0.596127	-0.17	EAL domain-containing protein	Biofilm formation - Vibrio cholerae
CsakCS931_RS12325	SF0137	0.343203	0.507408	-0.31	DnaK suppressor protein	Biofilm formation - Escherichia coli
CsakCS931_RS13000	Z6004	0.014625	0.057235	-0.88	MULTISPECIES: two-component system response regulator ArcA	Two-component system; Biofilm formation - Escherichia coli
CsakCS931_RS14160	ntrA	0.050545	0.147741	-0.61	MULTISPECIES: RNA polymerase factor sigma-54	Two-component system; Biofilm formation - Vibrio cholerae
CsakCS931_RS14195	SF3250	0.145585	0.29931	-0.30	aerobic respiration two-component sensor histidine kinase ArcB	Two-component system; Biofilm formation - Escherichia coli
CsakCS931_RS14925	STY3620	0.000268	0.002139	-1.24	class I adenylate cyclase	Purine metabolism; Biofilm formation - Escherichia coli; Biofilm formation - Vibrio cholerae
CsakCS931_RS15010	b3787	0.800669	0.873256	-0.06	MULTISPECIES: UDP-N-acetyl-D-mannosamine dehydrogenase	Amino sugar and nucleotide sugar metabolism; Two-component

						system; Biofilm formation - Vibrio cholerae
CsakCS931_RS15015	b3786	0.105703	0.244677	-0.55	MULTISPECIES: UDP-N-acetylglucosamine 2-epimerase (non-hydrolyzing)	Amino sugar and nucleotide sugar metabolism; Two-component system; Biofilm formation - Vibrio cholerae
CsakCS931_RS15180	b3961	0.12956	0.277553	-0.58	MULTISPECIES: DNA-binding transcriptional regulator OxyR	Biofilm formation - Escherichia coli
CsakCS931_RS15825	PA0082	0.642139	0.762031	-0.13	type VI secretion system protein TssA	Biofilm formation - Pseudomonas aeruginosa
CsakCS931_RS17460	ESA_04311	5.45E-06	8.19E-05	-1.34	MULTISPECIES: glucose-1-phosphate adenylyltransferase	Starch and sucrose metabolism; Amino sugar and nucleotide sugar metabolism; Biofilm formation - Escherichia coli
CsakCS931_RS17465	ESA_04312	0.002795	0.014604	-1.01	MULTISPECIES: glycogen synthase GlgA	Starch and sucrose metabolism; Biofilm formation - Escherichia coli

CsakCS931_RS17470	SF3451	0.000871	0.005571	-0.96	MULTISPECIES: glycogen phosphorylase	Starch and sucrose metabolism; Biofilm formation - Escherichia coli; Necroptosis; Insulin signaling pathway; Glucagon signaling pathway; Insulin resistance
CsakCS931_RS17570	Z4760	1.72E-05	0.000209	-1.08	hypothetical protein ESA_04334	Two-component system; Biofilm formation - Escherichia coli
CsakCS931_RS17575	b3404	0.092605	0.224783	-0.38	MULTISPECIES: two-component system sensor histidine kinase EnvZ	Two-component system; Biofilm formation - Escherichia coli
CsakCS931_RS17760	cap	0.193618	0.355621	-0.45	cAMP-activated global transcriptional regulator CRP	Two-component system; Quorum sensing; Biofilm formation - Pseudomonas aeruginosa; Biofilm formation - Escherichia coli;

CsakCS931_RS18715	SNSL254_A4722	0.022096	0.079108	-0.76	RNA chaperone Hfq [Erwinia sp. 198]	Biofilm formation - Vibrio cholerae Quorum sensing; RNA degradation; Biofilm formation - Vibrio cholerae
CsakCS931_RS19565	Z4389	0.064708	0.177713	-0.74	MULTISPECIES: 3',5'-cyclic-AMP phosphodiesterase	Purine metabolism; Biofilm formation - Pseudomonas aeruginosa
CsakCS931_RS20140	b2808	0.019362	0.071969	-0.85	MULTISPECIES: glycine cleavage system transcriptional regulator GcvA	Biofilm formation - Escherichia coli
CsakCS931_RS20785	PA0085	1.73E-09	9.41E-08	-2.59	type VI secretion system tube protein Hcp	Biofilm formation - Pseudomonas aeruginosa; Bacterial secretion system
CsakCS931_RS20845	/	0.433822	0.590651	-0.36	type VI secretion protein ImpG	Biofilm formation - Pseudomonas aeruginosa; Bacterial secretion system
CsakCS931_RS08375	c1327	7.63E-05	0.000749	-1.62	MULTISPECIES: biofilm formation regulator BssS	/

CsakCS931_RS03000	STM2118	0.176908	0.337253	0.72	MULTISPECIES: polysaccharide export protein	Two-component system; Biofilm formation - Escherichia coli
CsakCS931_RS03075	b2047	0.84356	0.900091	0.14	MULTISPECIES: undecaprenyl-phosphate glucose phosphotransferase	Biofilm formation - Vibrio cholerae
CsakCS931_RS03490	rcsA	0.068592	0.184586	0.47	MULTISPECIES: transcriptional regulator RcsA	Two-component system; Quorum sensing; Biofilm formation - Escherichia coli
CsakCS931_RS03700	STM1956	0.869634	0.915382	0.04	MULTISPECIES: RNA polymerase sigma factor FliA	Two-component system; Biofilm formation - Pseudomonas aeruginosa; Biofilm formation - Escherichia coli; Flagellar assembly; Biofilm formation - Vibrio cholerae
CsakCS931_RS03705	STY2163	0.88676	0.927785	0.06	MULTISPECIES: flagella biosynthesis regulatory protein FliZ	Biofilm formation - Escherichia coli
CsakCS931_RS05965	c0492	0.665705	0.778586	0.13	sensor domain-containing diguanylate cyclase	Biofilm formation - Escherichia coli

CsakCS931_RS06940	PA0083	0.218422	0.382335	0.66	MULTISPECIES: type VI secretion system contractile sheath small subunit	Biofilm formation - Pseudomonas aeruginosa
CsakCS931_RS08320	STM1172	0.053216	0.152883	0.67	MULTISPECIES: anti-sigma-28 factor FlgM	Two-component system; Biofilm formation - Pseudomonas aeruginosa; Biofilm formation - Escherichia coli; Flagellar assembly
CsakCS931_RS14435	b3261	0.323722	0.487787	0.42	MULTISPECIES: DNA-binding transcriptional regulator Fis	Biofilm formation - Vibrio cholerae
CsakCS931_RS15740	PA0090	4.01E-05	0.000436	1.45	type VI secretion system ATPase TssH	Biofilm formation - Pseudomonas aeruginosa; Bacterial secretion system
CsakCS931_RS15750	/	8.08E-05	0.000782	1.35	MULTISPECIES: type VI secretion system baseplate subunit TssG	Biofilm formation - Pseudomonas aeruginosa
CsakCS931_RS15795	PA0085	0.796461	0.871242	0.11	MULTISPECIES: type VI secretion system tube protein Hcp	Biofilm formation - Pseudomonas aeruginosa; Bacterial secretion system

CsakCS931_RS15815	PA0084	0.465815	0.621451	0.18	MULTISPECIES: type VI secretion system contractile sheath large subunit	Biofilm formation - Pseudomonas aeruginosa
CsakCS931_RS15830	/	0.189516	0.350864	0.36	MULTISPECIES: type VI secretion system-associated protein TagF	Biofilm formation - Pseudomonas aeruginosa
CsakCS931_RS15835	PA0077	0.241079	0.406518	0.30	MULTISPECIES: type VI secretion system membrane subunit TssM	Biofilm formation - Pseudomonas aeruginosa; Bacterial secretion system
CsakCS931_RS15845	PA0079	6.04E-05	0.000619	1.23	MULTISPECIES: type VI secretion system baseplate subunit TssK	Biofilm formation - Pseudomonas aeruginosa
CsakCS931_RS16670	STM3699	0.068229	0.184286	0.57	serine acetyltransferase	Cysteine and methionine metabolism; Sulfur metabolism; Biofilm formation - Vibrio cholerae
CsakCS931_RS16805	PA5267	0.339953	0.504354	1.48	type VI secretion system tube protein Hcp	Biofilm formation - Pseudomonas aeruginosa; Bacterial secretion system

CsakCS931_RS17070	b3533	0.160569	0.317228	0.38	UDP-forming cellulose synthase catalytic subunit	Starch and sucrose metabolism; Biofilm formation - Escherichia coli
CsakCS931_RS17110	b3525	0.062052	0.172269	0.85	cyclic-guanylate-specific phosphodiesterase	Biofilm formation - Escherichia coli
CsakCS931_RS17510	b3417	0.763731	0.848019	0.08	maltodextrin phosphorylase	Starch and sucrose metabolism; Biofilm formation - Escherichia coli; Necroptosis; Insulin signaling pathway; Glucagon signaling pathway; Insulin resistance
CsakCS931_RS20685	Arnit_2199	0.002641	0.013976	1.13	metallophosphoesterase	Purine metabolism; Biofilm formation - Pseudomonas aeruginosa
CsakCS931_RS20830	PA0083	0.224519	0.388989	0.65	MULTISPECIES: type VI secretion system contractile sheath small subunit	Biofilm formation - Pseudomonas aeruginosa

CsakCS931_RS20835	PA0084	0.336517	0.500888	0.55	type VI secretion system contractile sheath large subunit	Biofilm formation - Pseudomonas aeruginosa
CsakCS931_RS20855	/	0.7441	0.832734	0.13	type VI secretion system baseplate subunit TssG	Biofilm formation - Pseudomonas aeruginosa

Table S7. Genes related to antibiotic resistance and virulence in XY001-Ofl group.

	Gene Id	Gene	p-Value	q-Value	log_2Foldchange	Description	Pathway Description
Genes associated with antibiotic resistance	CsakCS931_RS20685	Arnit_2199	2.36E-18	4.81E-17	-10.73	metallophosphoesterase	Purine metabolism; Biofilm formation - Pseudomonas aeruginosa
	CsakCS931_RS08810	ECP_0940	######	######	-8.27	outer membrane protein F	beta-Lactam resistance; Two-component system
	CsakCS931_RS08895	SCH_0941	0.165686	0.256632	-0.40	MULTISPECIES: lipid A ABC transporter ATP-binding protein/permease MsbA	ABC transporters
	CsakCS931_RS16295	STM3997	3.64E-05	0.000157	-0.99	MULTISPECIES: thiol:disulfide interchange protein DsbA	Cationic antimicrobial peptide (CAMP) resistance
	CsakCS931_RS16700	ESA_04127	0.188207	0.285114	-0.34	MULTISPECIES: 6-phosphofructokinase	Glycolysis / Gluconeogenesis; Pentose phosphate pathway; Fructose and mannose metabolism; Galactose metabolism;

						Methane metabolism; RNA degradation; HIF-1 signaling pathway; AMPK signaling pathway; Glucagon signaling pathway; Central carbon metabolism in cancer
CsakCS931_RS02425	SL1344_2237	0.102283	0.173824	0.46	MULTISPECIES: porin OmpC	beta-Lactam resistance; Two-component system
CsakCS931_RS07550	STM1289	0.000523	0.00177	-1.11	MULTISPECIES: D-hexose-6-phosphate mutarotase	Glycolysis / Gluconeogenesis
CsakCS931_RS08030	SF1149	0.974974	0.98247	-0.01	MULTISPECIES: two-component system response regulator PhoP	Cationic antimicrobial peptide (CAMP) resistance; Two-component system
CsakCS931_RS09500		2.82E-17	4.79E-16	10.33	DUF2724 domain-containing protein	/
CsakCS931_RS05485	T1E_0241	1.83E-08	1.22E-07	1.54	efflux transporter outer membrane subunit	beta-Lactam resistance; Quorum sensing

CsakCS931_RS04145	b1846	8.46E-05	0.000341	1.13	tellurite resistance TerB family protein	/
CsakCS931_RS05510	PA1129	0.300766	0.414456	0.32	FosA family fosfomycin resistance glutathione transferase	/
CsakCS931_RS05615	b1430	0.328442	0.442443	-0.30	tellurite resistance methyltransferase TehB	/
CsakCS931_RS05900		0.043519	0.085301	-0.76	MULTISPECIES: multiple antibiotic resistance protein MarB	/
CsakCS931_RS06505	b0543	0.384224	0.498466	0.24	Ethidium bromide-methyl viologen resistance protein EmrE	/
CsakCS931_RS12435	traT	0.003891	0.010695	-1.09	MULTISPECIES: complement resistance protein TraT	/
CsakCS931_RS13270	ACIAD3023	0.301006	0.414539	0.31	MULTISPECIES: organic hydroperoxide resistance protein	/
CsakCS931_RS15350	b3910	0.121758	0.200108	-0.39	6-N-hydroxylaminopurine resistance protein	/
CsakCS931_RS15870	b3673	0.224294	0.327724	-0.33	multidrug resistance protein D	/
CsakCS931_RS16990	ACIAD3023	1.12E-06	6.06E-06	1.23	organic hydroperoxide resistance protein	/
CsakCS931_RS17805	BSU00260	0.550995	0.652395	0.17	toxic anion resistance protein	/
CsakCS931_RS18790	b4189	0.502343	0.61066	-0.18	biofilm peroxide resistance protein BsmA	/
CsakCS931_RS20370		1.58E-09	1.16E-08	7.65	MULTISPECIES: tellurite resistance TerB family protein	/

CsakCS931_RS00895	ESA_00711	0.682717	0.76603	0.12	hypothetical protein ESA_00711	Glycine, serine and threonine metabolism; Cyanoamino acid metabolism; Glyoxylate and dicarboxylate metabolism; One carbon pool by folate; Methane metabolism; Antifolate resistance
CsakCS931_RS01400	b2435	0.812079	0.870613	0.08	MULTISPECIES: N-acetylmuramoyl-L-alanine amidase AmiA	Cationic antimicrobial peptide (CAMP) resistance
CsakCS931_RS02150	b2301	0.003228	0.009053	0.85	glutathione transferase	Glutathione metabolism; Metabolism of xenobiotics by cytochrome P450; Drug metabolism - cytochrome P450; Drug metabolism - other enzymes;

						Platinum drug resistance; Longevity regulating pathway - worm; Pathways in cancer; Chemical carcinogenesis; Hepatocellular carcinoma; Fluid shear stress and atherosclerosis
CsakCS931_RS04545	Z1953	0.000366	0.001281	-0.88	MULTISPECIES: catabolic alanine racemase DadX	D-Alanine metabolism; Vancomycin resistance
CsakCS931_RS04885	STM1746	3.90E-07	2.23E-06	1.05	MULTISPECIES: oligopeptide ABC transporter substrate-binding protein OppA	beta-Lactam resistance; ABC transporters; Quorum sensing
CsakCS931_RS04890	Z2020	0.035233	0.071786	0.51	MULTISPECIES: oligopeptide ABC transporter permease OppB	beta-Lactam resistance; ABC transporters; Quorum sensing
CsakCS931_RS04895	Z2021	0.538314	0.642042	-0.17	MULTISPECIES: oligopeptide ABC transporter permease OppC	beta-Lactam resistance; ABC

						transporters; Quorum sensing
CsakCS931_RS04900	b1246	0.171698	0.264236	0.33	MULTISPECIES: ABC transporter ATP-binding protein	beta-Lactam resistance; ABC transporters; Quorum sensing
CsakCS931_RS04905	STM1742	0.009812	0.024226	-0.69	MULTISPECIES: ABC transporter ATP-binding protein	beta-Lactam resistance; ABC transporters; Quorum sensing
CsakCS931_RS05150	STM1696	0.032952	0.067658	0.56	MULTISPECIES: peptide ABC transporter ATP-binding protein SapF	Cationic antimicrobial peptide (CAMP) resistance; ABC transporters
CsakCS931_RS05155	STM1695	0.000174	0.000656	0.92	MULTISPECIES: peptide ABC transporter ATP-binding protein SapD	Cationic antimicrobial peptide (CAMP) resistance; ABC transporters
CsakCS931_RS05160	SF1297	3.07E-05	0.000134	-1.22	peptide ABC transporter permease SapC	Cationic antimicrobial peptide (CAMP) resistance; ABC transporters

CsakCS931_RS05165	STY1368	0.049851	0.095491	0.52	MULTISPECIES: peptide ABC transporter permease SapB	Cationic antimicrobial peptide (CAMP) resistance; ABC transporters
CsakCS931_RS05170	STM1692	0.077909	0.138532	0.51	MULTISPECIES: peptide ABC transporter substrate-binding protein SapA	Cationic antimicrobial peptide (CAMP) resistance; ABC transporters
CsakCS931_RS05315	b1329	0.000228	0.000839	1.22	peptide ABC transporter substrate-binding protein	beta-Lactam resistance; ABC transporters; Quorum sensing
CsakCS931_RS05905	Z2170	0.918567	0.945976	-0.03	MULTISPECIES: MDR efflux pump AcrAB transcriptional activator MarA	Cationic antimicrobial peptide (CAMP) resistance
CsakCS931_RS06210	ampC	0.529188	0.635352	-0.14	class C beta-lactamase CSA-1	beta-Lactam resistance; Two-component system
CsakCS931_RS06735	Z2647	0.000491	0.001672	1.10	MULTISPECIES: glutathione transferase GstA	Glutathione metabolism; Metabolism of xenobiotics by cytochrome P450;

						Drug metabolism - cytochrome P450; Drug metabolism - other enzymes; Platinum drug resistance; Longevity regulating pathway - worm; Pathways in cancer; Chemical carcinogenesis; Hepatocellular carcinoma; Fluid shear stress and atherosclerosis
CsakCS931_RS06805	STM4293	0.105034	0.177705	0.44	phosphoethanolamine transferase EptA	Lipopolysaccharide biosynthesis; Cationic antimicrobial peptide (CAMP) resistance
CsakCS931_RS08035	STY1270	0.154645	0.242945	0.42	two-component system sensor histidine kinase PhoQ	Cationic antimicrobial peptide (CAMP)

						resistance; Two-component system
CsakCS931_RS08100	b1113	9.46E-06	4.49E-05	1.11	MULTISPECIES: L,D-transpeptidase family protein	Cationic antimicrobial peptide (CAMP) resistance
CsakCS931_RS08130	ESA_02237	0.267675	0.376789	0.23	beta-N-acetylhexosaminidase	Amino sugar and nucleotide sugar metabolism; Glycosaminoglycan degradation; beta-Lactam resistance
CsakCS931_RS08610		0.879097	0.920222	-0.05	HARLDQ motif MBL-fold protein	beta-Lactam resistance
CsakCS931_RS08640	b1243	4.01E-09	2.82E-08	1.46	peptide ABC transporter substrate-binding protein	beta-Lactam resistance; ABC transporters; Quorum sensing
CsakCS931_RS09230	c0923	8.50E-08	5.28E-07	1.19	MULTISPECIES: glutathione S-transferase family protein	Glutathione metabolism; Metabolism of xenobiotics by cytochrome P450; Drug metabolism - cytochrome P450;

						Drug metabolism - other enzymes; Platinum drug resistance; Longevity regulating pathway - worm; Pathways in cancer; Chemical carcinogenesis; Hepatocellular carcinoma; Fluid shear stress and atherosclerosis
CsakCS931_RS10325	Z0781	0.449746	0.561937	-0.23	MULTISPECIES: peptidoglycan DD-transpeptidase MrdA	Peptidoglycan biosynthesis; beta-Lactam resistance
CsakCS931_RS10385	crcA	0.000435	0.001492	-0.99	MULTISPECIES: phospholipid:lipid A palmitoyltransferase	Lipopolysaccharide biosynthesis; Cationic antimicrobial peptide (CAMP) resistance; Pertussis
CsakCS931_RS10760	STM0498	0.042706	0.083869	0.44	copper-exporting P-type ATPase CopA	Platinum drug resistance; MAPK

						signaling pathway - plant
CsakCS931_RS10890	Z0578	0.000764	0.002508	0.86	MULTISPECIES: multidrug efflux RND transporter periplasmic adaptor subunit AcrA	beta-Lactam resistance; Cationic antimicrobial peptide (CAMP) resistance
CsakCS931_RS10895	b0462	0.000365	0.001276	0.77	MULTISPECIES: multidrug efflux RND transporter permease subunit	beta-Lactam resistance; Cationic antimicrobial peptide (CAMP) resistance
CsakCS931_RS11165	b0433	0.115012	0.191888	0.38	muropeptide MFS transporter AmpG	beta-Lactam resistance
CsakCS931_RS11440	b0381	0.682696	0.76603	-0.11	D-alanine--D-alanine ligase	D-Alanine metabolism; Peptidoglycan biosynthesis; Vancomycin resistance
CsakCS931_RS12090	b0192	0.106406	0.179305	-0.37	envelope stress response activation lipoprotein NlpE	Cationic antimicrobial peptide (CAMP) resistance

CsakCS931_RS12160	ESA_03160	0.39305	0.506926	0.25	MULTISPECIES: acyl-ACP--UDP-N-acetylglucosamine O-acyltransferase	Lipopolysaccharide biosynthesis; Cationic antimicrobial peptide (CAMP) resistance
CsakCS931_RS12245	Z0173	2.31E-07	1.36E-06	1.37	MULTISPECIES: serine endoprotease DegP	Cationic antimicrobial peptide (CAMP) resistance; Two-component system
CsakCS931_RS12560	b0092	0.611695	0.703672	-0.12	D-alanine--D-alanine ligase	D-Alanine metabolism; Peptidoglycan biosynthesis; Vancomycin resistance
CsakCS931_RS12570	ESA_03248	0.774178	0.842554	0.06	MULTISPECIES: undecaprenyldiphospho-muramoylpentapeptide beta-N-acetylglucosaminyltransferase	Peptidoglycan biosynthesis; Vancomycin resistance; Cell cycle - Caulobacter
CsakCS931_RS12585	ESA_03251	0.601441	0.695515	0.11	MULTISPECIES: phospho-N-acetylmuramoyl-pentapeptide-transferase	Peptidoglycan biosynthesis;

						Vancomycin resistance
CsakCS931_RS12590	b0086	0.253855	0.36274	-0.32	UDP-N-acetylmuramoyl-tripeptide--D-alanyl-D-alanine ligase	Lysine biosynthesis; Peptidoglycan biosynthesis; Vancomycin resistance
CsakCS931_RS12600	b0084	0.197747	0.296914	0.32	MULTISPECIES: peptidoglycan glycosyltransferase FtsI	Peptidoglycan biosynthesis; beta-Lactam resistance
CsakCS931_RS12770	atsR	0.096835	0.165824	0.45	Dihydrofolate reductase	One carbon pool by folate; Folate biosynthesis; Antifolate resistance
CsakCS931_RS14055	W5S_4173	0.449925	0.561937	-0.18	two-component system response regulator PmrA	Cationic antimicrobial peptide (CAMP) resistance; Two-component system
CsakCS931_RS14060	W5S_4174	3.43E-11	2.92E-10	1.63	two-component system sensor histidine kinase PmrB	Cationic antimicrobial peptide (CAMP) resistance; Two-component system

CsakCS931_RS14515	ESA_03670	0.482729	0.593523	0.16	MULTISPECIES: bifunctional phosphoribosylaminoimidazolecarboxamide formyltransferase/IMP cyclohydrolase	Purine metabolism; One carbon pool by folate; Antifolate resistance
CsakCS931_RS15245	b3941	0.018644	0.04196	-0.70	methylenetetrahydrofolate reductase	One carbon pool by folate; Carbon fixation pathways in prokaryotes; Antifolate resistance
CsakCS931_RS16115	SF3809	0.09258	0.160178	0.40	glutamine--fructose-6-phosphate transaminase (isomerizing)	Alanine, aspartate and glutamate metabolism; Amino sugar and nucleotide sugar metabolism; Insulin resistance
CsakCS931_RS16680	b3911	0.122192	0.20056	0.44	MULTISPECIES: envelope stress sensor histidine kinase CpxA	Cationic antimicrobial peptide (CAMP) resistance; Two-component system
CsakCS931_RS16685	Z5457	0.006541	0.01699	0.72	MULTISPECIES: envelope stress response regulator transcription factor CpxR	Cationic antimicrobial peptide (CAMP)

						resistance; Two-component system
CsakCS931_RS16995	STM3635	0.08434	0.148247	0.35	kdo(2)-lipid A phosphoethanolamine 7''-transferase	Lipopolysaccharide biosynthesis; Cationic antimicrobial peptide (CAMP) resistance
CsakCS931_RS17470	SF3451	8.56E-07	4.70E-06	1.16	MULTISPECIES: glycogen phosphorylas	Starch and sucrose metabolism; Biofilm formation - Escherichia coli; Necroptosis; Insulin signaling pathway; Glucagon signaling pathway; Insulin resistance
CsakCS931_RS17510	b3417	0.405251	0.517906	-0.18	maltodextrin phosphorylase	Starch and sucrose metabolism; Biofilm formation - Escherichia coli; Necroptosis; Insulin signaling pathway; Glucagon

						signaling pathway; Insulin resistance
CsakCS931_RS17615	b3396	0.384555	0.498605	0.24	MULTISPECIES: peptidoglycan glycosyltransferase/peptidoglycan DD-transpeptidase MrcA	Peptidoglycan biosynthesis; beta-Lactam resistance
CsakCS931_RS17730	STM3472	0.073919	0.133265	-0.57	MULTISPECIES: peptidylprolyl isomerase A	Cationic antimicrobial peptide (CAMP) resistance; Necroptosis
CsakCS931_RS18360	STY4443	0.575472	0.673493	0.21	alanine racemase	D-Alanine metabolism; Vancomycin resistance
CsakCS931_RS18435	gstA	7.97E-05	0.000323	0.80	glutathione S-transferase	Glutathione metabolism; Metabolism of xenobiotics by cytochrome P450; Drug metabolism - cytochrome P450; Drug metabolism - other enzymes; Platinum drug resistance; Longevity

						regulating pathway - worm; Pathways in cancer; Chemical carcinogenesis; Hepatocellular carcinoma; Fluid shear stress and atherosclerosis
CsakCS931_RS18700	b4169	0.306811	0.420814	0.23	N-acetylmuramoyl-L-alanine amidase AmiB	Cationic antimicrobial peptide (CAMP) resistance
CsakCS931_RS19550	b3035	0.000175	0.000659	0.97	MULTISPECIES: outer membrane channel protein TolC	beta-Lactam resistance; Cationic antimicrobial peptide (CAMP) resistance; Two-component system; Bacterial secretion system; Plant-pathogen interaction; Pertussis

	CsakCS931_RS20055	ESA_00482	0.005922	0.015581	0.73	thymidylate synthase	Pyrimidine metabolism; One carbon pool by folate; Antifolate resistance
	CsakCS931_RS20105	c3411	0.022486	0.048979	0.48	N-acetylmuramoyl-L-alanine amidase	Cationic antimicrobial peptide (CAMP) resistance
	CsakCS931_RS20610	ML0773	2.78E-20	7.60E-19	-11.13	MULTISPECIES: response regulator transcription factor	beta-Lactam resistance
Genes associated with bacterial virulence	CsakCS931_RS06360	/	0.439285	0.549444	0.17	virulence factor SrfB	/
	CsakCS931_RS06365	/	0.135874	0.216496	-0.49	virulence factor	/
	CsakCS931_RS07610	b0877	0.769738	0.836137	0.10	Virulence factor VirK	/
	CsakCS931_RS15765	PA0086	8.41E-05	0.000327	-1.25	protein of avirulence locus ImpE	/
	CsakCS931_RS16410	ESA_04062	0.003535	0.009623	-0.76	virulence factor BrkB family protein	/
Genes associated with Biofilms	CsakCS931_RS08375	c1327	0.701842	0.781106	0.13	MULTISPECIES: biofilm formation regulator BssS	
	CsakCS931_RS00150	STM2924	0.257468	0.363039	0.32	MULTISPECIES: RNA polymerase sigma factor RpoS	Biofilm formation - Escherichia coli; Biofilm formation - Vibrio cholerae

CsakCS931_RS00250	ECDH10B_2864	0.008299	0.02036	-0.76	MULTISPECIES: carbon storage regulator CsrA	Two-component system; Biofilm formation - Pseudomonas aeruginosa; Biofilm formation - Escherichia coli; Biofilm formation - Vibrio cholerae
CsakCS931_RS00300	ESA_00581	0.018375	0.04085	-0.58	MULTISPECIES: S-ribosylhomocysteine lyase	Cysteine and methionine metabolism; Quorum sensing; Biofilm formation - Escherichia coli; Biofilm formation - Vibrio cholerae
CsakCS931_RS01175	b2479	0.001805	0.005285	-0.67	MULTISPECIES: glycine cleavage system transcriptional repressor	Biofilm formation - Escherichia coli
CsakCS931_RS01455	STM2433	0.564503	0.661132	-0.17	MULTISPECIES: PTS glucose transporter subunit IIA	Glycolysis / Gluconeogenesis; Starch and sucrose metabolism; Amino sugar and nucleotide sugar metabolism;

						Biofilm formation - Escherichia coli; Phosphotransferase system (PTS); Biofilm formation - Vibrio cholerae
CsakCS931_RS02410	STY2496	0.130539	0.209386	-0.31	Sensor kinase protein RcsC	Two-component system; Biofilm formation - Escherichia coli
CsakCS931_RS02415	SF2301	0.006089	0.015666	-0.78	MULTISPECIES: transcriptional regulator RcsB	Two-component system; Biofilm formation - Escherichia coli
CsakCS931_RS02420	b2216	0.007604	0.019	-0.70	Sensor-like histidine kinase RcsD	Two-component system; Biofilm formation - Escherichia coli
CsakCS931_RS02805	b2127	1.28E-07	7.55E-07	-1.16	MULTISPECIES: MerR family transcriptional regulator	Biofilm formation - Escherichia coli
CsakCS931_RS02975	b2067	0.00494	0.012995	-0.59	diguanylate cyclase	Biofilm formation - Escherichia coli
CsakCS931_RS03000	STM2118	0.13763	0.218898	0.47	MULTISPECIES: polysaccharide export protein	Two-component system; Biofilm formation - Escherichia coli

CsakCS931_RS03075	b2047	2.05E-26	1.39E-24	-4.08	MULTISPECIES: undecaprenyl-phosphate glucose phosphotransferase	Biofilm formation - Vibrio cholerae
CsakCS931_RS03470	SSON_2013	0.000452	0.001518	0.77	cellulose biosynthesis regulator YedQ	Biofilm formation - Escherichia coli
CsakCS931_RS03490	rcsA	1.17E-08	7.72E-08	1.60	MULTISPECIES: transcriptional regulator RcsA	Two-component system; Quorum sensing; Biofilm formation - Escherichia coli
CsakCS931_RS03700	STM1956	0.461335	0.569442	0.18	MULTISPECIES: RNA polymerase sigma factor FliA	Two-component system; Biofilm formation - Pseudomonas aeruginosa; Biofilm formation - Escherichia coli; Flagellar assembly; Biofilm formation - Vibrio cholerae
CsakCS931_RS03705	STY2163	0.73563	0.807648	0.12	MULTISPECIES: flagella biosynthesis regulatory protein FliZ	Biofilm formation - Escherichia coli
CsakCS931_RS03730	b1916	0.750103	0.81979	0.10	MULTISPECIES: transcriptional regulator SdiA	Two-component system; Quorum sensing; Biofilm

						formation - Escherichia coli
CsakCS931_RS03740	SF1957	0.447909	0.557721	-0.24	MULTISPECIES: UvrY/SirA/GacA family response regulator transcription factor	Two-component system; Biofilm formation - Pseudomonas aeruginosa; Biofilm formation - Escherichia coli; Biofilm formation - Vibrio cholerae
CsakCS931_RS03870	ESA_01337	0.000533	0.001765	-0.96	Flagellar transcriptional activator FlhD	Two-component system; Quorum sensing; Biofilm formation - Escherichia coli; Flagellar assembly
CsakCS931_RS03875	STM1924	0.000567	0.001863	-1.00	MULTISPECIES: flagellar transcriptional regulator FlhC	Two-component system; Quorum sensing; Biofilm formation - Escherichia coli; Flagellar assembly
CsakCS931_RS04310	b1815	0.973933	0.983805	-0.01	EAL domain-containing protein	Biofilm formation - Escherichia coli

CsakCS931_RS04570	STM1798	0.853995	0.902341	0.04	MULTISPECIES: flagellar brake protein YcgR	Biofilm formation - Escherichia coli
CsakCS931_RS05000	STM1723	0.20193	0.299269	-0.40	anthranilate synthase subunit I	Phenylalanine, tyrosine and tryptophan biosynthesis; Phenazine biosynthesis; Quorum sensing; Biofilm formation - Pseudomonas aeruginosa
CsakCS931_RS05130	b1285	0.002481	0.007042	-0.60	cyclic di-GMP phosphodiesterase	Quorum sensing; Biofilm formation - Escherichia coli
CsakCS931_RS05965	c0492	0.318473	0.427708	0.28	sensor domain-containing diguanylate cyclase	Biofilm formation - Escherichia coli
CsakCS931_RS06940	PA0083	0.170134	0.261007	0.53	MULTISPECIES: type VI secretion system contractile sheath small subunit	Biofilm formation - Pseudomonas aeruginosa
CsakCS931_RS08160	b1101	0.93912	0.960765	0.02	MULTISPECIES: PTS glucose transporter subunit IIBC	Glycolysis / Gluconeogenesis; Amino sugar and nucleotide sugar metabolism; Phosphotransferas

						e system (PTS); Biofilm formation - Vibrio cholerae
CsakCS931_RS08320	STM1172	0.023234	0.049464	0.68	MULTISPECIES: anti-sigma-28 factor FlgM	Two-component system; Biofilm formation - Pseudomonas aeruginosa; Biofilm formation - Escherichia coli; Flagellar assembly
CsakCS931_RS11590	VC_0137	0.360213	0.471535	-0.20	EAL domain-containing protein	Biofilm formation - Vibrio cholerae
CsakCS931_RS12325	SF0137	0.01662	0.037436	-0.63	DnaK suppressor protein	Biofilm formation - Escherichia coli
CsakCS931_RS13000	Z6004	0.000298	0.001034	-1.04	MULTISPECIES: two-component system response regulator ArcA	Two-component system; Biofilm formation - Escherichia coli
CsakCS931_RS14160	ntrA	0.027721	0.057737	-0.54	MULTISPECIES: RNA polymerase factor sigma-54	Two-component system; Biofilm formation - Vibrio cholerae
CsakCS931_RS14195	SF3250	0.022789	0.048654	-0.46	aerobic respiration two-component sensor histidine kinase ArcB	Two-component system; Biofilm

						formation - Escherichia coli
CsakCS931_RS14435	b3261	0.019548	0.042933	0.88	MULTISPECIES: DNA-binding transcriptional regulator Fis [Bacteria]	Biofilm formation - Vibrio cholerae
CsakCS931_RS14440	PA1727	2.64E-23	1.13E-21	-11.97	MULTISPECIES: bifunctional diguanylate cyclase/phosphodiesterase	Biofilm formation - Pseudomonas aeruginosa
CsakCS931_RS14925	STY3620	3.47E-05	0.000145	-1.15	class I adenylate cyclase	Purine metabolism; Biofilm formation - Escherichia coli; Biofilm formation - Vibrio cholerae
CsakCS931_RS15010	b3787	0.000278	0.00097	-0.83	MULTISPECIES: UDP-N-acetyl-D-mannosamine dehydrogenase	Amino sugar and nucleotide sugar metabolism; Two-component system; Biofilm formation - Vibrio cholerae
CsakCS931_RS15015	b3786	0.672741	0.755564	0.12	MULTISPECIES: UDP-N-acetylglucosamine 2-epimerase (non-hydrolyzing)	Amino sugar and nucleotide sugar metabolism; Two-component system; Biofilm

						formation - Vibrio cholerae
CsakCS931_RS15180	b3961	0.248672	0.353753	-0.34	MULTISPECIES: DNA-binding transcriptional regulator OxyR	Biofilm formation - Escherichia coli
CsakCS931_RS15740	PA0090	0.192656	0.287867	0.40	type VI secretion system ATPase TssH	Biofilm formation - Pseudomonas aeruginosa; Bacterial secretion system
CsakCS931_RS15750		0.105844	0.176307	0.47	MULTISPECIES: type VI secretion system baseplate subunit TssG	Biofilm formation - Pseudomonas aeruginosa
CsakCS931_RS15795	PA0085	0.00013	0.000487	1.35	MULTISPECIES: type VI secretion system tube protein Hcp	Biofilm formation - Pseudomonas aeruginosa; Bacterial secretion system
CsakCS931_RS15815	PA0084	0.020657	0.044853	0.53	MULTISPECIES: type VI secretion system contractile sheath large subunit	Biofilm formation - Pseudomonas aeruginosa
CsakCS931_RS15825	PA0082	0.189565	0.283966	0.34	type VI secretion system protein TssA	Biofilm formation - Pseudomonas aeruginosa
CsakCS931_RS15830		2.71E-06	1.34E-05	1.30	MULTISPECIES: type VI secretion system-associated protein TagF	Biofilm formation - Pseudomonas aeruginosa

CsakCS931_RS15835	PA0077	0.537761	0.637301	0.14	MULTISPECIES: type VI secretion system membrane subunit TssM	Biofilm formation - Pseudomonas aeruginosa; Bacterial secretion system
CsakCS931_RS15845	PA0079	0.179207	0.272238	0.33	MULTISPECIES: type VI secretion system baseplate subunit TssK	Biofilm formation - Pseudomonas aeruginosa
CsakCS931_RS16670	STM3699	0.866167	0.910989	0.05	serine acetyltransferase	Cysteine and methionine metabolism; Sulfur metabolism; Biofilm formation - Vibrio cholerae
CsakCS931_RS16805	PA5267	2.64E-17	4.43E-16	-4.15	type VI secretion system tube protein Hcp	Biofilm formation - Pseudomonas aeruginosa; Bacterial secretion system
CsakCS931_RS17070	b3533	0.035775	0.071549	-0.52	UDP-forming cellulose synthase catalytic subunit	Starch and sucrose metabolism; Biofilm formation - Escherichia coli
CsakCS931_RS17110	b3525	0.008206	0.02018	0.91	cyclic-guanylate-specific phosphodiesterase	Biofilm formation - Escherichia coli

CsakCS931_RS17460	ESA_04311	3.93E-08	2.45E-07	-1.30	MULTISPECIES: glucose-1-phosphate adenylyltransferase	Starch and sucrose metabolism; Amino sugar and nucleotide sugar metabolism; Biofilm formation - Escherichia coli
CsakCS931_RS17465	ESA_04312	9.51E-08	5.68E-07	-1.40	MULTISPECIES: glycogen synthase GlgA	Starch and sucrose metabolism; Biofilm formation - Escherichia coli
CsakCS931_RS17470	SF3451	1.35E-06	6.99E-06	-1.13	MULTISPECIES: glycogen phosphorylase	Starch and sucrose metabolism; Biofilm formation - Escherichia coli; Necroptosis; Insulin signaling pathway; Glucagon signaling pathway; Insulin resistance
CsakCS931_RS17510	b3417	0.331577	0.441601	0.21	maltodextrin phosphorylase	Starch and sucrose metabolism; Biofilm formation - Escherichia coli; Necroptosis;

						Insulin signaling pathway; Glucagon signaling pathway; Insulin resistance
CsakCS931_RS17570	Z4760	3.39E-18	6.37E-17	-1.79	hypothetical protein ESA_04334	Two-component system; Biofilm formation - Escherichia coli
CsakCS931_RS17575	b3404	0.114762	0.188663	-0.33	MULTISPECIES: two-component system sensor histidine kinase EnvZ	Two-component system; Biofilm formation - Escherichia coli
CsakCS931_RS17760	cap	0.351136	0.461943	-0.26	cAMP-activated global transcriptional regulator CRP	Two-component system; Quorum sensing; Biofilm formation - Pseudomonas aeruginosa; Biofilm formation - Escherichia coli; Biofilm formation - Vibrio cholerae
CsakCS931_RS18715	SNSL254_A 4722	0.005898	0.015219	-0.73	RNA chaperone Hfq	Quorum sensing; RNA degradation;

CsakCS931_RS19565	Z4389	0.016342	0.036984	-0.74	MULTISPECIES: 3',5'-cyclic-AMP phosphodiesterase	Biofilm formation - Vibrio cholerae Purine metabolism; Biofilm formation - Pseudomonas aeruginosa
CsakCS931_RS20140	b2808	0.177491	0.270486	-0.40	MULTISPECIES: glycine cleavage system transcriptional regulator GcvA	Biofilm formation - Escherichia coli
CsakCS931_RS20685	Arnit_2199	2.00E-18	4.03E-17	10.76	metallophosphoesterase	Purine metabolism; Biofilm formation - Pseudomonas aeruginosa
CsakCS931_RS20785	PA0085	9.78E-15	1.18E-13	9.64	type VI secretion system tube protein Hcp	Biofilm formation - Pseudomonas aeruginosa; Bacterial secretion system
CsakCS931_RS20830	PA0083	0.006092	0.015667	1.11	MULTISPECIES: type VI secretion system contractile sheath small subunit	Biofilm formation - Pseudomonas aeruginosa
CsakCS931_RS20835	PA0084	7.25E-05	0.000287	1.70	type VI secretion system contractile sheath large subunit	Biofilm formation - Pseudomonas aeruginosa

CsakCS931_RS20845	1.91E-09	1.35E-08	7.67	type VI secretion protein ImpG	Biofilm formation - Pseudomonas aeruginosa; Bacterial secretion system
CsakCS931_RS20855	1.50E-14	1.77E-13	8.38	type VI secretion system baseplate subunit TssG	Biofilm formation - Pseudomonas aeruginosa

Table S8. Genes related to antibiotic resistance and virulence in XY001-Ceo group.

	Gene id	Gene	p-Value	q-Value	log_2Foldchange	Description	Pathway Description
Genes associated with antibiotic resistance	CsakCS931_RS16295	STM3997	0.074116	0.165933	-0.73	MULTISPECIES: thiol:disulfide interchange protein DsbA	Cationic antimicrobial peptide (CAMP) resistance
	CsakCS931_RS12245	Z0173	0.001207	0.010455	-1.07	MULTISPECIES: serine endoprotease DegP	Cationic antimicrobial peptide (CAMP) resistance; Two-component system
	CsakCS931_RS06210	ampC	2.86E-54	2.34E-51	4.66	class C beta-lactamase CSA-1	beta-Lactam resistance; Two-component system
	CsakCS931_RS13395	BSU34160	0.015341	0.059054	-0.68	MULTISPECIES: extracellular solute-binding protein	ABC transporters
	CsakCS931_RS08100	b1113	0.038727	0.108751	-0.71	MULTISPECIES: L,D-transpeptidase family protein	Cationic antimicrobial peptide (CAMP) resistance
	CsakCS931_RS00895	ESA_00711	1.16E-07	7.61E-06	-2.48	hypothetical protein ESA_00711	Glycine, serine and threonine metabolism; Cyanoamino acid metabolism; Glyoxylate and

						dicarboxylate metabolism; One carbon pool by folate; Methane metabolism; Antifolate resistance
CsakCS931_RS01400	b2435	3.43E-06	0.000113	-2.12	MULTISPECIES: N-acetylmuramoyl-L-alanine amidase AmiA	Cationic antimicrobial peptide (CAMP) resistance
CsakCS931_RS02150	b2301	0.411541	0.533523	0.24	glutathione transferase	Glutathione metabolism; Metabolism of xenobiotics by cytochrome P450; Drug metabolism - cytochrome P450; Drug metabolism - other enzymes; Platinum drug resistance; Longevity regulating pathway - worm; Pathways in cancer; Chemical carcinogenesis;

						Hepatocellular carcinoma; Fluid shear stress and atherosclerosis
CsakCS931_RS02425	SL1344_2237	0.000234	0.003138	-2.42	MULTISPECIES: porin OmpC	beta-Lactam resistance; Two-component system
CsakCS931_RS04545	Z1953	0.957087	0.970775	0.03	MULTISPECIES: catabolic alanine racemase DadX	D-Alanine metabolism; Vancomycin resistance
CsakCS931_RS04890	Z2020	0.129232	0.239346	0.35	MULTISPECIES: oligopeptide ABC transporter permease OppB	beta-Lactam resistance; ABC transporters; Quorum sensing
CsakCS931_RS04895	Z2021	0.229893	0.355898	0.31	MULTISPECIES: oligopeptide ABC transporter permease OppC	beta-Lactam resistance; ABC transporters; Quorum sensing
CsakCS931_RS04905	STM1742	0.078115	0.171747	0.46	MULTISPECIES: ABC transporter ATP-binding protein	beta-Lactam resistance; ABC transporters; Quorum sensing
CsakCS931_RS05160	SF1297	0.733556	0.795713	0.19	peptide ABC transporter permease SapC	Cationic antimicrobial peptide (CAMP)

						resistance; ABC transporters
CsakCS931_RS05485	T1E_0241	0.322974	0.448359	0.32	efflux transporter outer membrane subunit	beta-Lactam resistance; Quorum sensing
CsakCS931_RS05905	Z2170	7.99E-05	0.001384	-1.56	MULTISPECIES: MDR efflux pump AcrAB transcriptional activator MarA	Cationic antimicrobial peptide (CAMP) resistance
CsakCS931_RS06735	Z2647	0.050989	0.129333	-1.24	MULTISPECIES: glutathione transferase GstA	Glutathione metabolism; Metabolism of xenobiotics by cytochrome P450; Drug metabolism - cytochrome P450; Drug metabolism - other enzymes; Platinum drug resistance; Longevity regulating pathway - worm; Pathways in cancer; Chemical carcinogenesis; Hepatocellular

						carcinoma; Fluid shear stress and atherosclerosis
CsakCS931_RS08610		0.00576	0.031661	0.82	HARLDQ motif MBL-fold protein	beta-Lactam resistance
CsakCS931_RS08640	b1243	0.032284	0.09636	0.46	peptide ABC transporter substrate-binding protein	beta-Lactam resistance; ABC transporters; Quorum sensing
CsakCS931_RS09230	c0923	0.079805	0.173981	0.76	MULTISPECIES: glutathione S-transferase family protein	Glutathione metabolism; Metabolism of xenobiotics by cytochrome P450; Drug metabolism - cytochrome P450; Drug metabolism - other enzymes; Platinum drug resistance; Longevity regulating pathway - worm; Pathways in cancer; Chemical carcinogenesis; Hepatocellular

						carcinoma; Fluid shear stress and atherosclerosis
CsakCS931_RS10890	Z0578	0.004328	0.025845	-1.27	MULTISPECIES: multidrug efflux RND transporter periplasmic adaptor subunit AcrA	beta-Lactam resistance; Cationic antimicrobial peptide (CAMP) resistance
CsakCS931_RS10895	b0462	0.067397	0.155651	-0.53	MULTISPECIES: multidrug efflux RND transporter permease subunit	beta-Lactam resistance; Cationic antimicrobial peptide (CAMP) resistance
CsakCS931_RS11440	b0381	0.521483	0.627751	0.15	D-alanine--D-alanine ligase	D-Alanine metabolism; Peptidoglycan biosynthesis; Vancomycin resistance
CsakCS931_RS12090	b0192	0.002215	0.01633	-1.04	envelope stress response activation lipoprotein NlpE	Cationic antimicrobial peptide (CAMP) resistance
CsakCS931_RS12160	ESA_03160	0.014505	0.057774	-1.03	MULTISPECIES: acyl-ACP--UDP-N-acetylglucosamine O-acyltransferase	Lipopolysaccharide biosynthesis; Cationic

						antimicrobial peptide (CAMP) resistance
CsakCS931_RS12570	ESA_03248	0.606253	0.694144	0.16	MULTISPECIES: undecaprenyldiphospho-muramoylpentapeptide beta-N-acetylglucosaminyltransferase	Peptidoglycan biosynthesis; Vancomycin resistance; Cell cycle - Caulobacter
CsakCS931_RS12585	ESA_03251	0.565314	0.661196	0.12	MULTISPECIES: phospho-N-acetylmuramoyl-pentapeptide-transferase	Peptidoglycan biosynthesis; Vancomycin resistance
CsakCS931_RS14060	W5S_4174	0.572764	0.66768	0.16	two-component system sensor histidine kinase PmrB	Cationic antimicrobial peptide (CAMP) resistance; Two-component system
CsakCS931_RS15245	b3941	0.02174	0.074383	0.58	methylenetetrahydrofolate reductase	One carbon pool by folate; Carbon fixation pathways in prokaryotes; Antifolate resistance
CsakCS931_RS16685	Z5457	0.06621	0.154148	-1.04	MULTISPECIES: envelope stress response regulator transcription factor CpxR	Cationic antimicrobial peptide (CAMP)

						resistance; Two-component system
CsakCS931_RS17510	b3417	0.226261	0.352513	0.23	maltodextrin phosphorylase	Starch and sucrose metabolism; Biofilm formation - Escherichia coli; Necroptosis; Insulin signaling pathway; Glucagon signaling pathway; Insulin resistance
CsakCS931_RS17615	b3396	0.002255	0.016559	-1.14	MULTISPECIES: peptidoglycan glycosyltransferase/peptidoglycan DD-transpeptidase MrcA	Peptidoglycan biosynthesis; beta-Lactam resistance
CsakCS931_RS17730	STM3472	9.21E-08	6.45E-06	-2.50	MULTISPECIES: peptidylprolyl isomerase A	Cationic antimicrobial peptide (CAMP) resistance; Necroptosis
CsakCS931_RS18360	STY4443	0.015165	0.05893	-1.22	alanine racemase	D-Alanine metabolism; Vancomycin resistance
CsakCS931_RS18435	gstA	0.202468	0.328165	-0.34	glutathione S-transferase	Glutathione metabolism; Metabolism of

						xenobiotics by cytochrome P450; Drug metabolism - cytochrome P450; Drug metabolism - other enzymes; Platinum drug resistance; Longevity regulating pathway - worm; Pathways in cancer; Chemical carcinogenesis; Hepatocellular carcinoma; Fluid shear stress and atherosclerosis
CsakCS931_RS18700	b4169	0.941327	0.959129	-0.04	N-acetylmuramoyl-L-alanine amidase AmiB	Cationic antimicrobial peptide (CAMP) resistance
CsakCS931_RS19550	b3035	7.86E-05	0.001371	-1.98	MULTISPECIES: outer membrane channel protein TolC	beta-Lactam resistance; Cationic antimicrobial peptide (CAMP) resistance; Two-

							component system; Bacterial secretion system; Plant-pathogen interaction; Pertussis
	CsakCS931_RS20055	ESA_00482	7.19E-05	0.001289	-1.52	thymidylate synthase	Pyrimidine metabolism; One carbon pool by folate; Antifolate resistance
	CsakCS931_RS20105	c3411	0.463253	0.578407	-0.16	N-acetylmuramoyl-L-alanine amidase	Cationic antimicrobial peptide (CAMP) resistance
	CsakCS931_RS20610	ML0773	0.128419	0.238049	-0.36	MULTISPECIES: response regulator transcription factor	beta-Lactam resistance
Genes associated with bacterial virulence	CsakCS931_RS06360	/	0.014349	0.058305	1.03(up)	virulence factor SrfB	/
	CsakCS931_RS06365	/	0.467985	0.58501	0.67(up)	virulence factor	/
	CsakCS931_RS07610	b0877	0.001846	0.01469	1.52(down)	Virulence factor VirK	/
	CsakCS931_RS15765	PA0086	0.818718	0.862659	0.18(up)	protein of avirulence locus ImpE	/
	CsakCS931_RS16410	ESA_04062	0.137973	0.252041	0.34(up)	virulence factor BrkB family protein	/

Genes associated with Biofilms	CsakCS931_RS00150	STM2924	0.000196	0.002836	2.17	MULTISPECIES: RNA polymerase sigma factor RpoS	Biofilm formation - Escherichia coli; Biofilm formation - Vibrio cholerae
	CsakCS931_RS00250	ECDH10B_2864	0.194868	0.322931	0.79	MULTISPECIES: carbon storage regulator CsrA	Two-component system; Biofilm formation - Pseudomonas aeruginosa; Biofilm formation - Escherichia coli; Biofilm formation - Vibrio cholerae
	CsakCS931_RS00300	ESA_00581	0.059875	0.146146	1.05	MULTISPECIES: S-ribosylhomocysteine lyase	Cysteine and methionine metabolism; Quorum sensing; Biofilm formation - Escherichia coli; Biofilm formation - Vibrio cholerae
	CsakCS931_RS01175	b2479	0.076675	0.172392	0.63	MULTISPECIES: glycine cleavage system transcriptional repressor	Biofilm formation - Escherichia coli
	CsakCS931_RS01455	STM2433	0.055473	0.139154	0.92	MULTISPECIES: PTS glucose transporter subunit IIA	Glycolysis / Gluconeogenesis; Starch and sucrose

						metabolism; Amino sugar and nucleotide sugar metabolism; Biofilm formation - Escherichia coli; Phosphotransferase system (PTS); Biofilm formation - Vibrio cholerae
CsakCS931_RS02410	STY2496	0.821473	0.864495	0.04	Sensor kinase protein RcsC	Two-component system; Biofilm formation - Escherichia coli
CsakCS931_RS02415	SF2301	0.202464	0.330496	0.81	MULTISPECIES: transcriptional regulator RcsB	Two-component system; Biofilm formation - Escherichia coli
CsakCS931_RS02420	b2216	0.118225	0.229423	0.89	Sensor-like histidine kinase RcsD	Two-component system; Biofilm formation - Escherichia coli
CsakCS931_RS02805	b2127	0.098352	0.202341	0.41	MULTISPECIES: MerR family transcriptional regulator	Biofilm formation - Escherichia coli
CsakCS931_RS02975	b2067	0.696999	0.768875	0.14	diguanylate cyclase	Biofilm formation - Escherichia coli

CsakCS931_RS03000	STM2118	0.135304	0.249535	-0.48	MULTISPECIES: polysaccharide export protein	Two-component system; Biofilm formation - Escherichia coli
CsakCS931_RS03075	b2047	0.127756	0.240812	-0.88	MULTISPECIES: undecaprenyl-phosphate glucose phosphotransferase	Biofilm formation - Vibrio cholerae
CsakCS931_RS03470	SSON_2013	0.010116	0.046867	0.79	cellulose biosynthesis regulator YedQ	Biofilm formation - Escherichia coli
CsakCS931_RS03490	rcsA	0.267961	0.39798	0.29	MULTISPECIES: transcriptional regulator RcsA	Two-component system; Quorum sensing; Biofilm formation - Escherichia coli
CsakCS931_RS03700	STM1956	0.438643	0.558885	0.18	MULTISPECIES: RNA polymerase sigma factor FliA	Two-component system; Biofilm formation - Pseudomonas aeruginosa; Biofilm formation - Escherichia coli; Flagellar assembly; Biofilm formation - Vibrio cholerae
CsakCS931_RS03705	STY2163	0.825177	0.866969	0.08	MULTISPECIES: flagella biosynthesis regulatory protein FliZ	Biofilm formation - Escherichia coli

CsakCS931_RS03730	b1916	0.027427	0.088316	1.20	MULTISPECIES: transcriptional regulator SdiA	Two-component system; Quorum sensing; Biofilm formation - Escherichia coli
CsakCS931_RS03740	SF1957	0.001589	0.0133	1.86	MULTISPECIES: UvrY/SirA/GacA family response regulator transcription factor	Two-component system; Biofilm formation - Pseudomonas aeruginosa; Biofilm formation - Escherichia coli; Biofilm formation - Vibrio cholerae
CsakCS931_RS03870	ESA_01337	0.000452	0.005318	2.14	Flagellar transcriptional activator FlhD	Two-component system; Quorum sensing; Biofilm formation - Escherichia coli; Flagellar assembly
CsakCS931_RS03875	STM1924	0.000241	0.003323	1.88	MULTISPECIES: flagellar transcriptional regulator FlhC	Two-component system; Quorum sensing; Biofilm formation - Escherichia coli; Flagellar assembly

CsakCS931_RS04310	b1815	0.322939	0.451868	-0.22	EAL domain-containing protein	Biofilm formation - Escherichia coli
CsakCS931_RS04570	STM1798	0.696707	0.768718	0.09	MULTISPECIES: flagellar brake protein YcgR	Biofilm formation - Escherichia coli
CsakCS931_RS05000	STM1723	0.054326	0.137235	-0.56	anthranilate synthase subunit I	Phenylalanine, tyrosine and tryptophan biosynthesis; Phenazine biosynthesis; Quorum sensing; Biofilm formation - Pseudomonas aeruginosa
CsakCS931_RS05130	b1285	0.491561	0.604744	0.18	cyclic di-GMP phosphodiesterase	Quorum sensing; Biofilm formation - Escherichia coli
CsakCS931_RS05965	c0492	0.632216	0.717507	-0.23	sensor domain-containing diguanylate cyclase	Biofilm formation - Escherichia coli
CsakCS931_RS06940	PA0083	0.055564	0.139154	-0.76	MULTISPECIES: type VI secretion system contractile sheath small subunit	Biofilm formation - Pseudomonas aeruginosa
CsakCS931_RS08160	b1101	0.122285	0.234523	0.70	MULTISPECIES: PTS glucose transporter subunit IIBC	Glycolysis / Gluconeogenesis; Amino sugar and nucleotide sugar

						metabolism; Phosphotransferase system (PTS); Biofilm formation - Vibrio cholerae
CsakCS931_RS08320	STM1172	0.00064	0.006804	1.69	MULTISPECIES: anti-sigma-28 factor FlgM	Two-component system; Biofilm formation - Pseudomonas aeruginosa; Biofilm formation - Escherichia coli; Flagellar assembly
CsakCS931_RS11590	VC_0137	0.137074	0.250934	-0.27	EAL domain-containing protein	Biofilm formation - Vibrio cholerae
CsakCS931_RS12325	SF0137	0.022572	0.077314	1.31	DnaK suppressor protein	Biofilm formation - Escherichia coli
CsakCS931_RS13000	Z6004	0.009914	0.046309	1.51	MULTISPECIES: two-component system response regulator ArcA	Two-component system; Biofilm formation - Escherichia coli
CsakCS931_RS14160	ntrA	0.05722	0.142052	1.08	MULTISPECIES: RNA polymerase factor sigma-54	Two-component system; Biofilm formation - Vibrio cholerae

CsakCS931_RS14195	SF3250	0.588908	0.685376	0.15	aerobic respiration two-component sensor histidine kinase ArcB	Two-component system; Biofilm formation - Escherichia coli
CsakCS931_RS14435	b3261	3.74E-08	3.17E-06	3.32	MULTISPECIES: DNA-binding transcriptional regulator Fis	Biofilm formation - Vibrio cholerae
CsakCS931_RS14925	STY3620	0.404365	0.528952	0.56	class I adenylate cyclase	Purine metabolism; Biofilm formation - Escherichia coli; Biofilm formation - Vibrio cholerae
CsakCS931_RS15010	b3787	0.127682	0.240812	0.37	MULTISPECIES: UDP-N-acetyl-D-mannosamine dehydrogenase	Amino sugar and nucleotide sugar metabolism; Two-component system; Biofilm formation - Vibrio cholerae
CsakCS931_RS15015	b3786	0.001439	0.01227	1.52	MULTISPECIES: UDP-N-acetylglucosamine 2-epimerase (non-hydrolyzing)	Amino sugar and nucleotide sugar metabolism; Two-component system; Biofilm formation - Vibrio cholerae
CsakCS931_RS15180	b3961	0.000809	0.008001	1.27	MULTISPECIES: DNA-binding transcriptional regulator OxyR	Biofilm formation - Escherichia coli

CsakCS931_RS15740	PA0090	0.694198	0.767432	-0.23	type VI secretion system ATPase TssH	Biofilm formation - Pseudomonas aeruginosa; Bacterial secretion system
CsakCS931_RS15750	/	0.691542	0.765657	-0.22	MULTISPECIES: type VI secretion system baseplate subunit TssG	Biofilm formation - Pseudomonas aeruginosa
CsakCS931_RS15795	PA0085	0.005251	0.030752	1.33	MULTISPECIES: type VI secretion system tube protein Hcp	Biofilm formation - Pseudomonas aeruginosa; Bacterial secretion system
CsakCS931_RS15815	PA0084	0.00084	0.00814	1.04	MULTISPECIES: type VI secretion system contractile sheath large subunit	Biofilm formation - Pseudomonas aeruginosa
CsakCS931_RS15825	PA0082	0.16535	0.28701	0.56	type VI secretion system protein TssA	Biofilm formation - Pseudomonas aeruginosa
CsakCS931_RS15830	/	0.586064	0.68343	-0.28	MULTISPECIES: type VI secretion system-associated protein TagF	Biofilm formation - Pseudomonas aeruginosa
CsakCS931_RS15835	PA0077	0.535239	0.641784	-0.23	MULTISPECIES: type VI secretion system membrane subunit TssM	Biofilm formation - Pseudomonas aeruginosa; Bacterial secretion system

CsakCS931_RS15845	PA0079	0.725161	0.790748	-0.16	MULTISPECIES: type VI secretion system baseplate subunit TssK	Biofilm formation - Pseudomonas aeruginosa
CsakCS931_RS16670	STM3699	0.7769	0.830899	-0.18	serine acetyltransferase	Cysteine and methionine metabolism; Sulfur metabolism; Biofilm formation - Vibrio cholerae
CsakCS931_RS16805	PA5267	0.171653	0.294558	-0.87	type VI secretion system tube protein Hcp	Biofilm formation - Pseudomonas aeruginosa; Bacterial secretion system
CsakCS931_RS17070	b3533	0.458826	0.57676	-0.31	UDP-forming cellulose synthase catalytic subunit	Starch and sucrose metabolism; Biofilm formation - Escherichia coli
CsakCS931_RS17110	b3525	0.139088	0.253535	-0.53	cyclic-guanylate-specific phosphodiesterase	Biofilm formation - Escherichia coli
CsakCS931_RS17460	ESA_04311	0.149411	0.266588	0.63	MULTISPECIES: glucose-1-phosphate adenylyltransferase	Starch and sucrose metabolism; Amino sugar and nucleotide sugar metabolism; Biofilm formation - Escherichia coli

CsakCS931_RS17465	ESA_04312	0.084855	0.184705	0.83	MULTISPECIES: glycogen synthase GlgA	Starch and sucrose metabolism; Biofilm formation - Escherichia coli
CsakCS931_RS17470	SF3451	0.180371	0.305676	0.54	MULTISPECIES: glycogen phosphorylase	Starch and sucrose metabolism; Biofilm formation - Escherichia coli; Necroptosis; Insulin signaling pathway; Glucagon signaling pathway; Insulin resistance
CsakCS931_RS17510	b3417	0.260357	0.388946	-0.22	maltodextrin phosphorylase	Starch and sucrose metabolism; Biofilm formation - Escherichia coli; Necroptosis; Insulin signaling pathway; Glucagon signaling pathway; Insulin resistance
CsakCS931_RS17570	Z4760	0.558033	0.660818	0.22	hypothetical protein ESA_04334	Two-component system; Biofilm formation - Escherichia coli

CsakCS931_RS17575	b3404	0.805139	0.851855	-0.04	MULTISPECIES: two-component system sensor histidine kinase EnvZ	Two-component system; Biofilm formation - Escherichia coli
CsakCS931_RS17760	cap	0.03315	0.099449	1.23	cAMP-activated global transcriptional regulator CRP	Two-component system; Quorum sensing; Biofilm formation - Pseudomonas aeruginosa; Biofilm formation - Escherichia coli; Biofilm formation - Vibrio cholerae
CsakCS931_RS18715	SNSL254_A4722	0.06842	0.160011	1.11	RNA chaperone Hfq	Quorum sensing; RNA degradation; Biofilm formation - Vibrio cholerae
CsakCS931_RS19565	Z4389	0.000187	0.002728	2.21	MULTISPECIES: 3',5'-cyclic-AMP phosphodiesterase	Purine metabolism; Biofilm formation - Pseudomonas aeruginosa
CsakCS931_RS20140	b2808	0.108218	0.215426	0.80	MULTISPECIES: glycine cleavage system transcriptional regulator GcvA	Biofilm formation - Escherichia coli

CsakCS931_RS20685	Arnit_2199	0.163207	0.284446	-0.99	metallophosphoesterase	Purine metabolism; Biofilm formation - Pseudomonas aeruginosa
CsakCS931_RS20785	PA0085	0.031375	0.095978	-0.89	type VI secretion system tube protein Hcp	Biofilm formation - Pseudomonas aeruginosa; Bacterial secretion system
CsakCS931_RS20830	PA0083	0.073201	0.166927	-0.73	MULTISPECIES: type VI secretion system contractile sheath small subunit	Biofilm formation - Pseudomonas aeruginosa
CsakCS931_RS20835	PA0084	0.130002	0.24338	-0.74	type VI secretion system contractile sheath large subunit	Biofilm formation - Pseudomonas aeruginosa
CsakCS931_RS20845	/	0.250731	0.380103	-0.47	type VI secretion protein ImpG	Biofilm formation - Pseudomonas aeruginosa; Bacterial secretion system
CsakCS931_RS20855	/	0.116615	0.227373	-0.55	type VI secretion system baseplate subunit TssG	Biofilm formation - Pseudomonas aeruginosa
CsakCS931_RS08375	c1327	0.482635	0.596628	-0.55	MULTISPECIES: biofilm formation regulator BssS	/

Table S9. Gene names and information used for qRT-PCR detection.

Number	Gene Symbol	Forward Primer (5->3)	Reverse Primer (5->3)	Product Length(bp)	Tm (℃)
1	16srRNA	CCTACGGCTACCTTGTTACGACTTC	TCGGAATCGCTAGTAATCGTGAATCAG		60
2	ampC	GCATGACGCACACCTTTATC	CGGTTTGTCGTCCTTGTTATAG	80	60
3	b1256	GCATCAAATCTCGCCGTGTC	TAGCGCCTGTTTACTGGCAA	71	60
4	z2647	ACGCTCGTCAGTGTGGATTT	TAATCGCTACGCCTTCCGTC	133	60
5	ACIAD3023	AAGAAATGGGCGGAATGGGT	TTCCGACGTCACCTTCAACA	150	60
6	MarB	GACGCGCTGGATATCAGTCA	GAGGTTGTAATACGGCACGC	75	60
7	BsmA	GCAGGAGATCCAGCGCTATC	CCGGTGACCCATACTGTAGC	77	60
8	b1113	TGACCCAGAACAAGGTGTCG	GCACTTCTTGCCCTGAGACT	94	60
9	b1846	GGGGTCCGCAATGAAGAAGA	AATGCCGTCACGAACATCCT	141	60
10	BSU00260	CACGGGCAAATGAAAGAGGC	CGGCGTTTGATTCGTCCTTC	118	60
11	ramA	TCAGATGGTTTCTCCGGTGC	TTCTTCTGCCGCTTCGACAT	112	60
12	b1256	TTCTGCAACCGTACGTCCAA	GGCCCAGTTGCGTGTTATTG	89	60
13	b4189	GACCAGACGCAAGGGTTACA	TCGTCCACCATGACGACAAC	137	60
14	DUF2724	CTGGATCGCAGGTAAGGACG	TCGCGGAAACAGCTTCGATA	118	60
15	TIE_0241	CAAACGCACTGGACACTTGG	CGGAAGAGAGATCGGAGTGC	148	60
16	b1243	TGATGTGATGGCGTGGAACA	CCGCTGCTCTAGATACGCAA	97	60
17	Arnit_2199	TAGGAAGAGAACGCTTAGCAG	TGAGAAGGTGAAGCAGCAGTTA	133	60
18	ML0773	TACTCGTGATGTGCTGCTCG	CACTCCTCGAACGGTCAACA	145	60
19	ECP_0940	TAACAACTCTGAGGGCGCTG	TCGGTGTAACCCAGTGCATC	138	60
20	ESA_00711	ACGAGGATATGGCGAAGCAG	CACGCATTTTTGCCCAGTCA	99	60
21	b2435	GAACAAGCGGCATTTGTGGT	GCTGTGGCGATTTTCTGACG	131	60
22	SL1344_2237	GGTGAAAACACCAACGGTCG	GCCTTCGCCGATTGCATAAG	84	60
23	STM3472	CTGGAGCTGAACAGCCAGAA	CGCGGTGAAACGTAGTGTTG	94	60

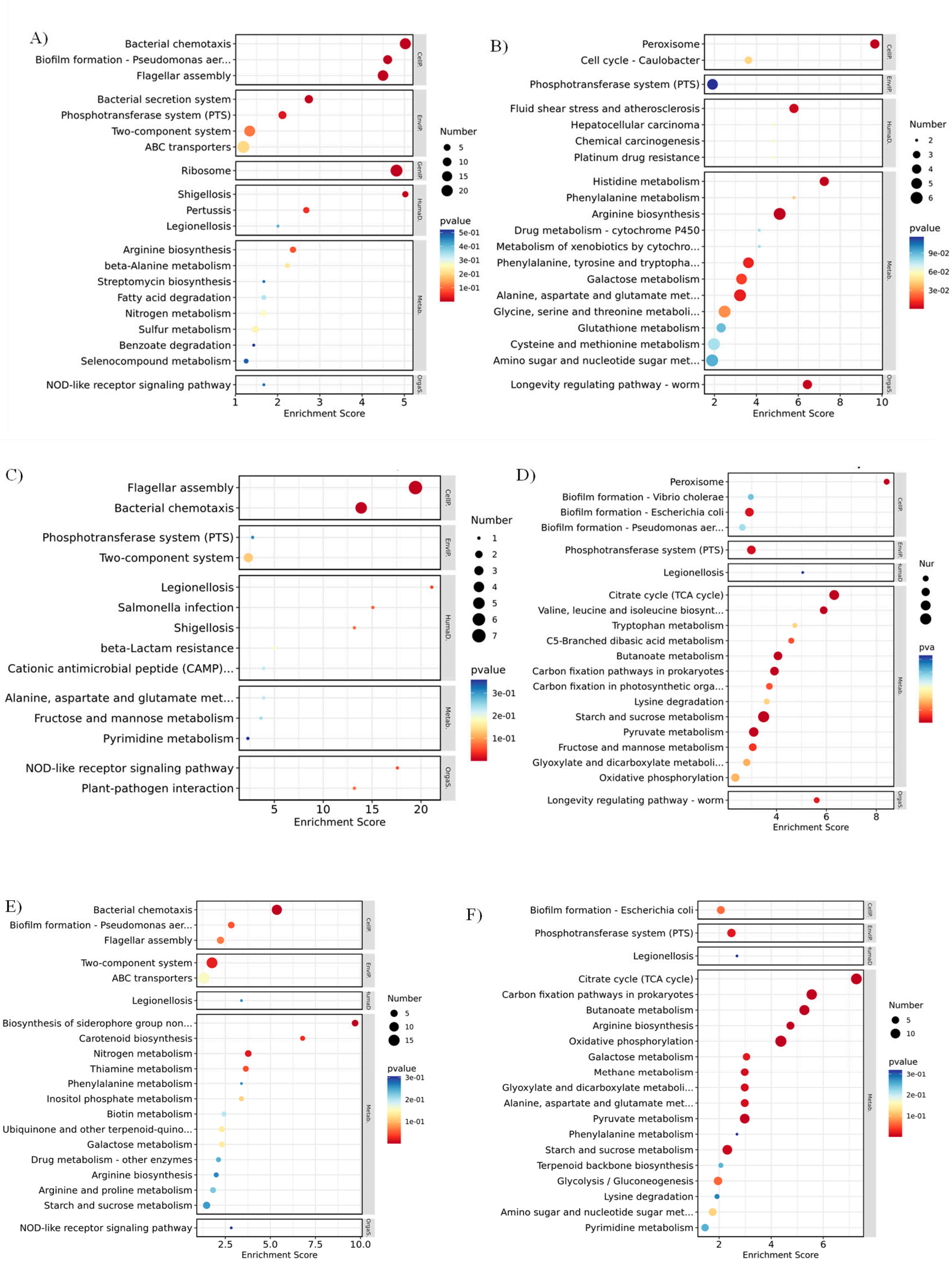
A)
Bacterial chemotaxis
Biofilm formation - Pseudomonas aer...
Flagellar assembly
Bacterial secretion system
Phosphotransferase system (PTS)
Two-component system
ABC transporters
Ribosome
Shigellosis
Pertussis
Legionellosis
Arginine biosynthesis
beta-Alanine metabolism
Streptomycin biosynthesis
Fatty acid degradation
Nitrogen metabolism
Sulfur metabolism
Benzoate degradation
Selenocompound metabolism
NOD-like receptor signaling pathway
Enrichment Score
Number
pvalue
B)
Peroxisome
Cell cycle - Caulobacter
Phosphotransferase system (PTS)
Fluid shear stress and atherosclerosis
Hepatocellular carcinoma
Chemical carcinogenesis
Platinum drug resistance
Histidine metabolism
Phenylalanine metabolism
Arginine biosynthesis
Drug metabolism - cytochrome P450
Metabolism of xenobiotics by cytochro...
Phenylalanine, tyrosine and tryptopha...
Galactose metabolism
Alanine, aspartate and glutamate met...
Glycine, serine and threonine metaboli...
Glutathione metabolism
Cysteine and methionine metabolism
Amino sugar and nucleotide sugar met...
Longevity regulating pathway - worm
Enrichment Score
Number
pvalue
C)
Flagellar assembly
Bacterial chemotaxis
Phosphotransferase system (PTS)
Two-component system
Legionellosis
Salmonella infection
Shigellosis
beta-Lactam resistance
Cationic antimicrobial peptide (CAMP)...
Alanine, aspartate and glutamate met...
Fructose and mannose metabolism
Pyrimidine metabolism
NOD-like receptor signaling pathway
Plant-pathogen interaction
Enrichment Score
Number
pvalue
D)
Peroxisome
Biofilm formation - Vibrio cholerae
Biofilm formation - Escherichia coli
Biofilm formation - Pseudomonas aer...
Phosphotransferase system (PTS)
Legionellosis
Citrate cycle (TCA cycle)
Valine, leucine and isoleucine biosynt...
Tryptophan metabolism
C5-Branched dibasic acid metabolism
Butanoate metabolism
Carbon fixation pathways in prokaryotes
Carbon fixation in photosynthetic orga...
Lysine degradation
Starch and sucrose metabolism
Pyruvate metabolism
Fructose and mannose metabolism
Glyoxylate and dicarboxylate metaboli...
Oxidative phosphorylation
Longevity regulating pathway - worm
Enrichment Score
E)
Bacterial chemotaxis
Biofilm formation - Pseudomonas aer...
Flagellar assembly
Two-component system
ABC transporters
Legionellosis
Biosynthesis of siderophore group non...
Carotenoid biosynthesis
Nitrogen metabolism
Thiamine metabolism
Phenylalanine metabolism
Inositol phosphate metabolism
Biotin metabolism
Ubiquinone and other terpenoid-quino...
Galactose metabolism
Drug metabolism - other enzymes
Arginine biosynthesis
Arginine and proline metabolism
Starch and sucrose metabolism
NOD-like receptor signaling pathway
Enrichment Score
Number
pvalue
F)
Biofilm formation - Escherichia coli
Phosphotransferase system (PTS)
Legionellosis
Citrate cycle (TCA cycle)
Carbon fixation pathways in prokaryotes
Butanoate metabolism
Arginine biosynthesis
Oxidative phosphorylation
Galactose metabolism
Methane metabolism
Glyoxylate and dicarboxylate metaboli...
Alanine, aspartate and glutamate met...
Pyruvate metabolism
Phenylalanine metabolism
Starch and sucrose metabolism
Terpenoid backbone biosynthesis
Glycolysis / Gluconeogenesis
Lysine degradation
Amino sugar and nucleotide sugar met...
Pyrimidine metabolism
Enrichment Score
Number
pvalue

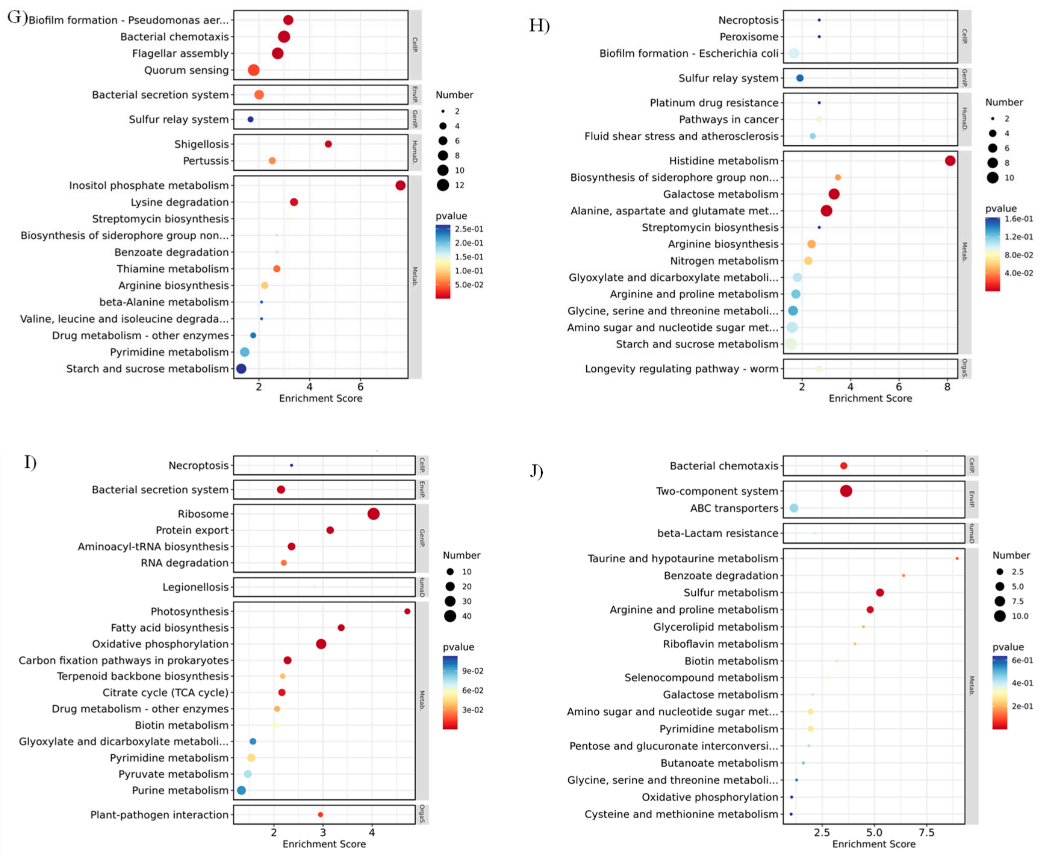

Figure S1. Scatter diagram of KEGG annotation enrichment pathways of DEGs in *C. sakazakii* strains induced with five antibiotics (**A**) XY001-Amp down-regulated gene; (**B**) XY001-Amp up-regulated genes; (**C**) XY001-Ami down-regulated gene; (**D**) XY001-Ami up-regulated genes; (**E**) XY001-Tet down-regulated genes; (**F**) XY001-Tet up-regulated genes; (**G**) XY001-Ofl down-regulated genes; (**H**) XY001-Ofl up-regulated genes; (**I**) XY001-Ceo down-regulated genes; (**J**) XY001-Ceo up-regulated genes.

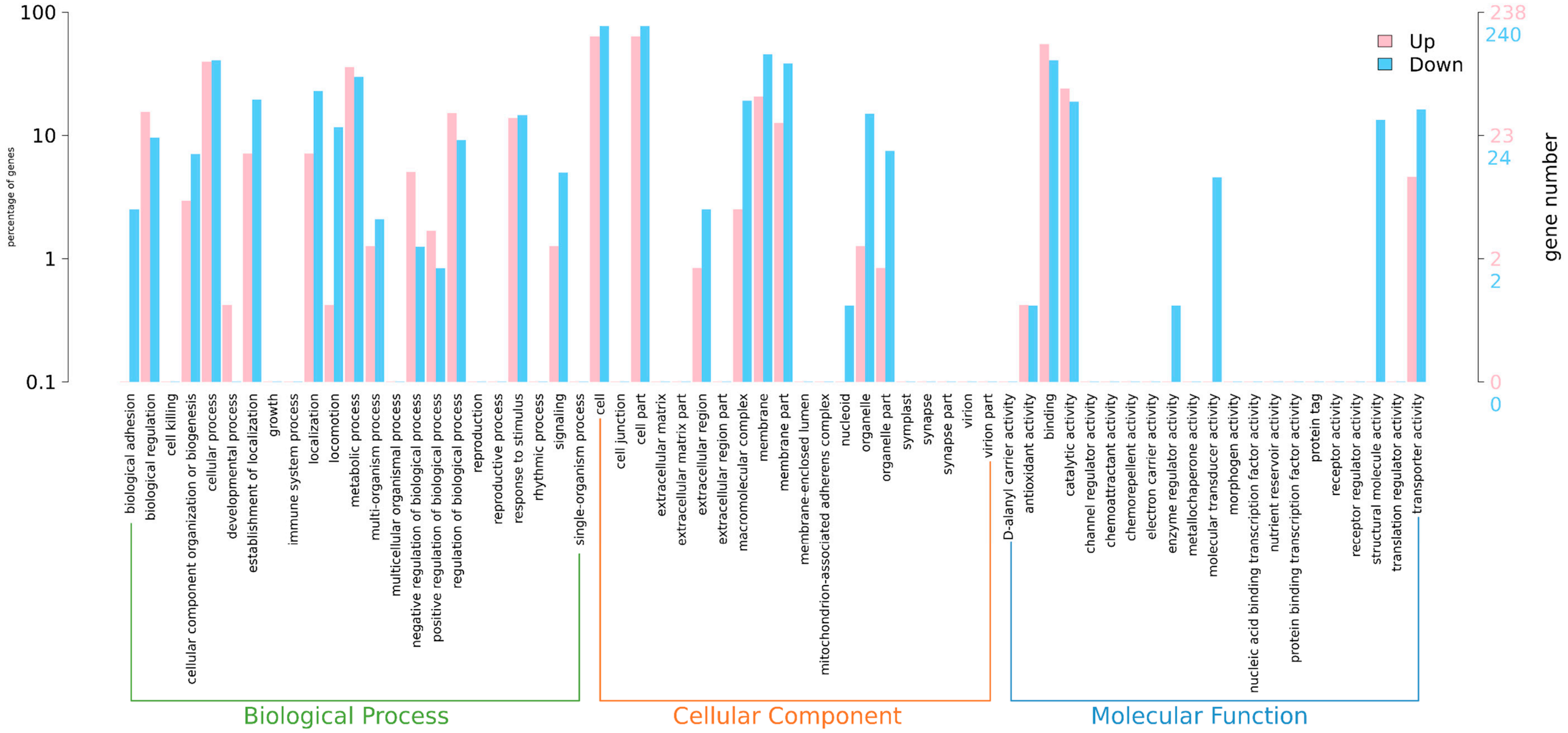

Figure S2. Histogram of GO-annotated enrichment terms of *C. sakazakii* DEGs after the induction with ampicillin.

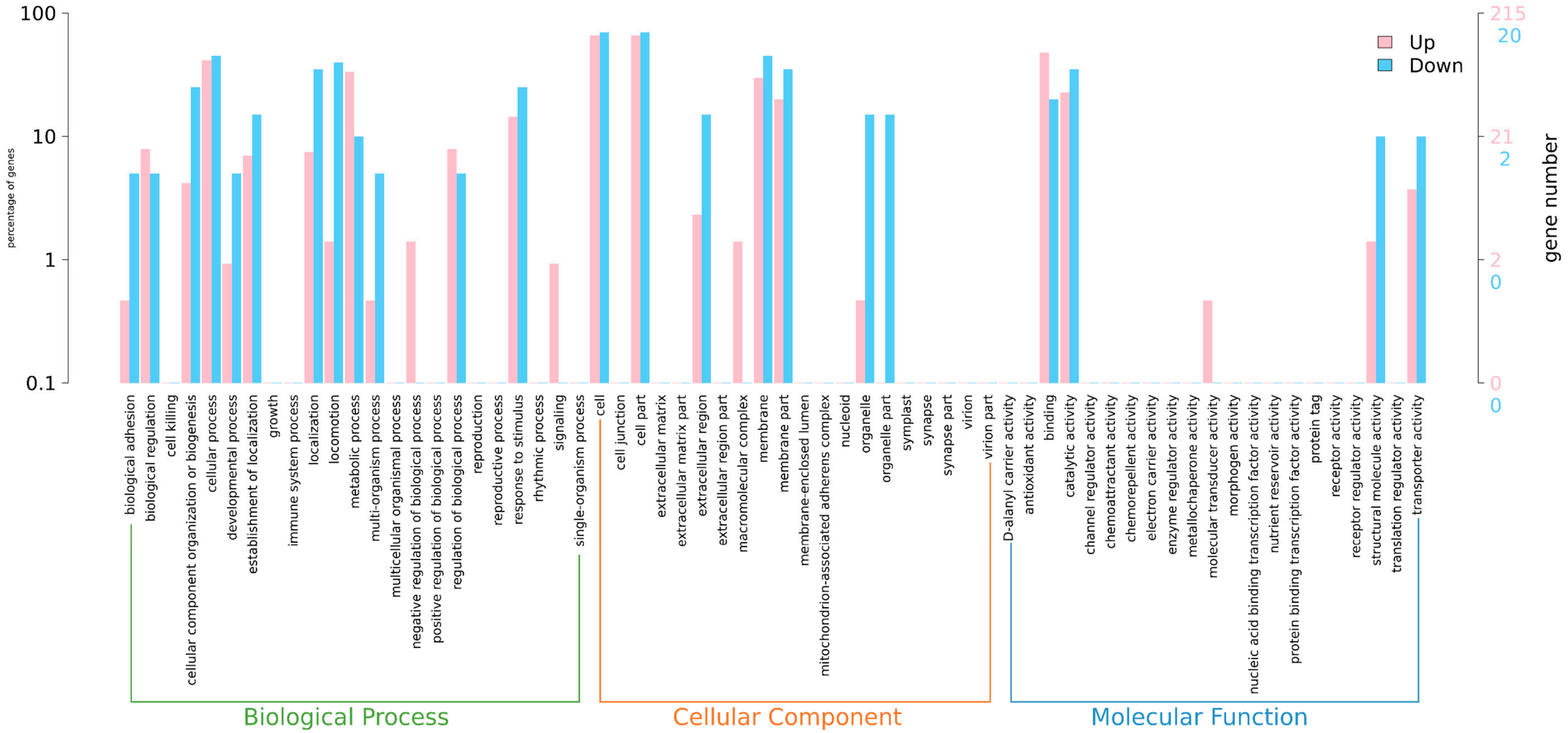

Figure S3. Histogram of GO-annotated enrichment terms of *C. sakazakii* DEGs after the induction with amikacin.

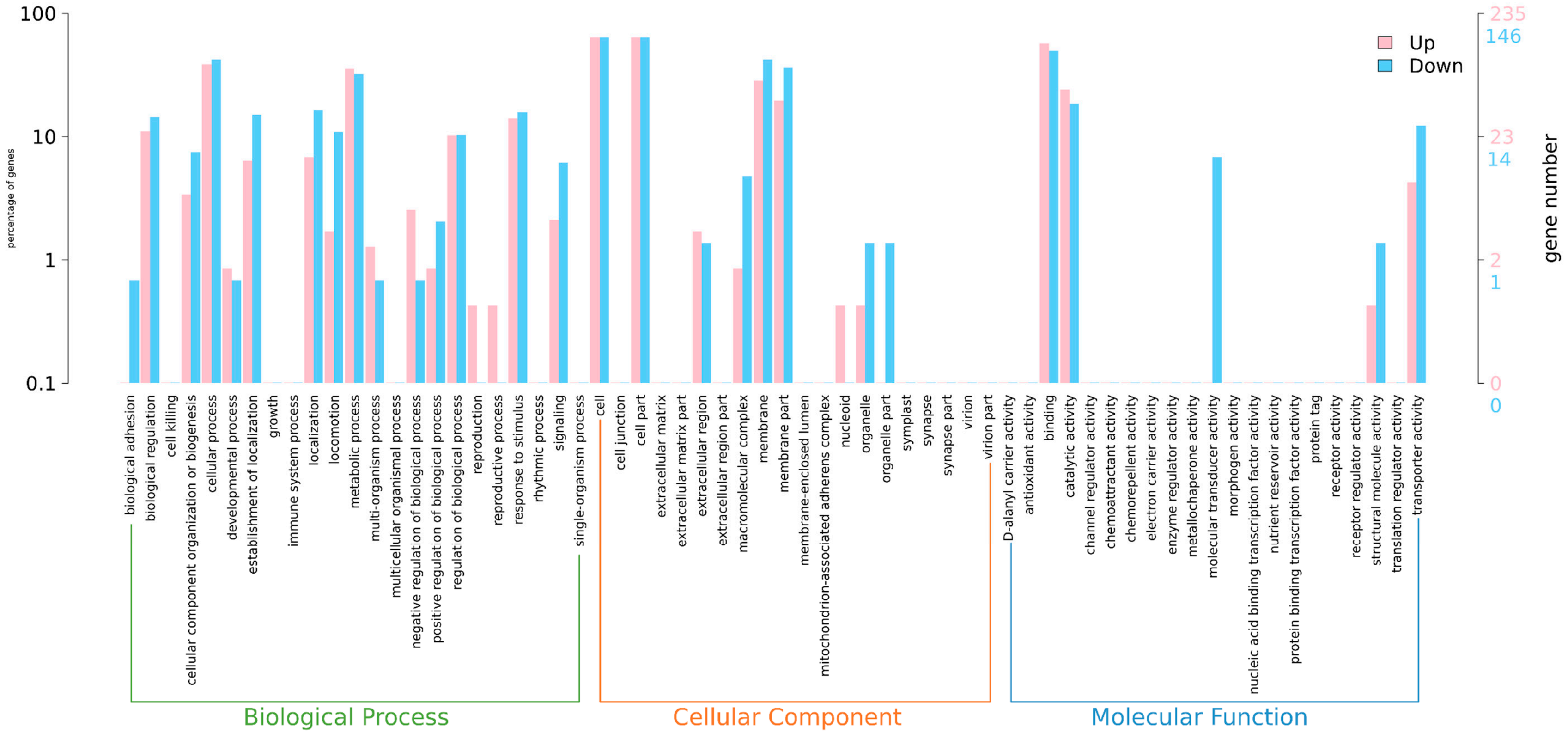

Figure S4. Histogram of GO-annotated enrichment terms of *C. sakazakii* DEGs after the induction with tetracycline.

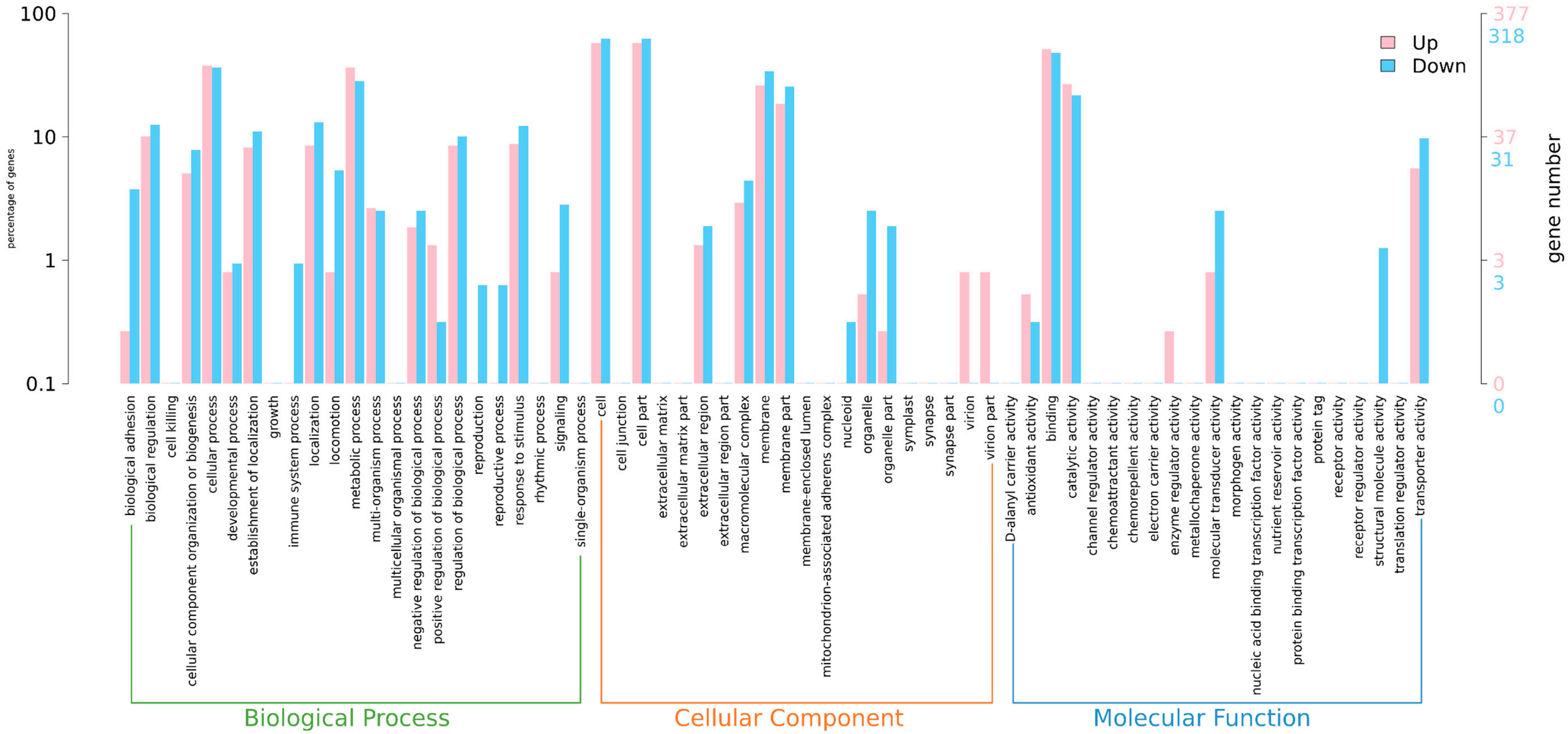

Figure S5. Histogram of GO-annotated enrichment terms of *C. sakazakii* DEGs after the induction with ofloxacin.

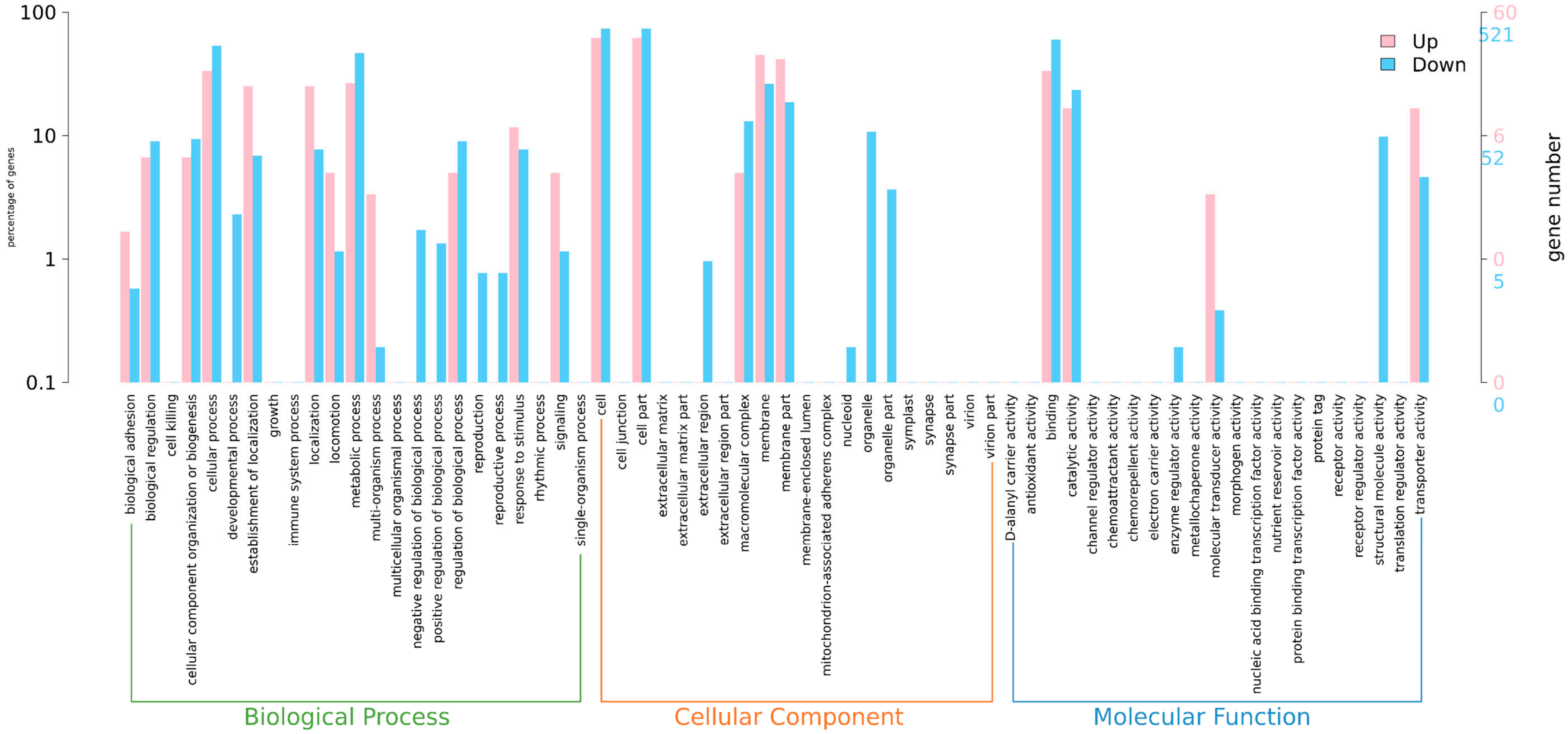

Figure S6. Histogram of GO-annotated enrichment terms of *C. sakazakii* DEGs after the induction with ceftriaxone.
